# STAT1-Dependent Signal Integration between IFNγ and TLR4 in Vascular Cells Reflect Pro-Atherogenic Responses in Human Atherosclerosis

**DOI:** 10.1371/journal.pone.0113318

**Published:** 2014-12-05

**Authors:** Stefan Chmielewski, Adam Olejnik, Krzysztof Sikorski, Jaroslav Pelisek, Katarzyna Błaszczyk, Cristiane Aoqui, Hanna Nowicka, Alma Zernecke, Uwe Heemann, Joanna Wesoly, Marcus Baumann, Hans A. R. Bluyssen

**Affiliations:** 1 Department of Nephrology, Klinikum rechts der Isar, Technical University Munich, Munich, Germany; 2 Department of Vascular and Endovascular Surgery, Klinikum rechts der Isar, Technical University Munich, Munich, Germany; 3 Department of Human Molecular Genetics, Institute of Molecular Biology and Biotechnology, Faculty of Biology, Adam Mickiewicz University Poznan, Poznan, Poland; 4 Laboratory of High-throughput Technologies, Institute of Molecular Biology and Biotechnology, Faculty of Biology, Adam Mickiewicz University Poznan, Poznan, Poland; 5 German Centre for Cardiovascular Research, partner site Munich Heart Alliance, Munich, Germany; University of Kansas Medical Center, United States of America

## Abstract

Signal integration between IFNγ and TLRs in immune cells has been associated with the host defense against pathogens and injury, with a predominant role of STAT1. We hypothesize that STAT1-dependent transcriptional changes in vascular cells involved in cross-talk between IFNγ and TLR4, reflect pro-atherogenic responses in human atherosclerosis. Genome-wide investigation identified a set of STAT1-dependent genes that were synergistically affected by interactions between IFNγ and TLR4 in VSMCs. These included the chemokines *Cxcl9*, *Ccl12*, *Ccl8*, *Ccrl2*, *Cxcl10* and *Ccl5*, adhesion molecules *Cd40*, *Cd74*, and antiviral and antibacterial genes *Rsad2*, *Mx1*, *Oasl1*, *Gbp5*, *Nos2*, *Batf2* and *Tnfrsf11a*. Among the amplified genes was also *Irf8*, of which *Ccl5* was subsequently identified as a new pro-inflammatory target in VSMCs and ECs. Promoter analysis predicted transcriptional cooperation between STAT1, IRF1, IRF8 and NFκB, with the novel role of IRF8 providing an additional layer to the overall complexity. The synergistic interactions between IFNγ and TLR4 also resulted in increased T-cell migration and impaired aortic contractility in a STAT1-dependent manner. Expression of the chemokines CXCL9 and CXCL10 correlated with STAT1 phosphorylation in vascular cells in plaques from human carotid arteries. Moreover, using data mining of human plaque transcriptomes, expression of a selection of these STAT1-dependent pro-atherogenic genes was found to be increased in coronary artery disease (CAD) and carotid atherosclerosis. Our study provides evidence to suggest that in ECs and VSMCs STAT1 orchestrates a platform for cross-talk between IFNγ and TLR4, and identifies a STAT1-dependent gene signature that reflects a pro-atherogenic state in human atherosclerosis.

## Introduction

Inflammation participates importantly in host defenses against infectious agents and injury, but it also contributes to the pathophysiology of many diseases including atherosclerosis. Atherosclerosis is characterized by early endothelial cell (EC) dysfunction and altered contractility of vascular smooth muscle cells (VSMCs) [1]. Recruitment of blood leukocytes to the injured vascular endothelium characterizes the initiation and progression of atherosclerosis and involves many inflammatory mediators, modulated by cells of both innate and adaptive immunity [2].

The pro-inflammatory cytokine interferon (IFN)-γ, derived from T-cells, is vital for both innate and adaptive immunity and is also expressed at high levels in atherosclerotic lesions. Evidence that IFNγ is necessary and sufficient to cause vascular remodeling is supported by mouse models of atheroma formation, as the serological neutralization or genetic absence of IFNγ markedly reduces the extent of atherosclerosis [3], [4], [5], [6]. The signal transduction pathway initiated by binding of IFNγ to its receptor leads to intracellular phosphorylation of signal transducer and activator of transcription (STAT)1. Subsequently, STAT1 homodimerizes and translocates into the nucleus where it binds to IFNγ-activated sequences (GAS elements) in the promoters of IFNγ-inducible genes or at other sites by further interaction with other transcription factors [7], including members of the Interferon Regulatory Factor (IRF) family [8], [9]. Thus, STAT1 plays a major role in mediating immune and pro-inflammatory responses. As such, IFNγ is considered to participate in promoting atherogenic responses through STAT1-mediated “damaging” signals, regulating the functions and properties of all cell types present in the vessel wall. Indeed, Agrawal et al. revealed that STAT1 positively influences lesion formation in experimental atherosclerosis *in vivo* and is required for optimal progression of foam cell formation in macrophages *in vitro* and *in vivo* [10]. However, the specific role for STAT1 in human atherosclerosis has not been previously reported.

STAT1 has also been identified as an important mediator in the biological response to different Toll like receptors (TLRs), which are innate immune pattern recognition receptors (PRR) expressed on a variety of cells, and initiate and sustain the inflammatory response in atherosclerosis [11]. Activation of TLR4 through lipopolysaccharide (LPS), which is mediated by both NFκB and IRF3, leads to the induction of various target genes including type I IFNs, pro-inflammatory cytokines, chemokines and cell surface molecules [12]. Some of these genes are regulated secondary to LPS-induced IFNβ, which after secretion binds to the type I IFN receptor to activate gene expression in a STAT1-dependent manner [7]. Cross-talk between IFNγ and TLRs has been associated with the host defense against pathogens and injury. IFNγ produced by T-cells and other cells is considered to enhance TLR signaling in dendritic cells and macrophages for the efficient induction of inflammatory mediators to eliminate pathogens [13], [14]. STAT1 has been identified as a critical mediator in this cross-talk between IFNγ and TLR signaling pathways [15], [16]. Consequently, the cooperation of STAT1 with other transcription factors, including IRFs and NFκB, coordinate the antimicrobial and inflammatory synergism between IFNγ and TLRs in immune cells. Recently, we showed that also in ECs and VSMCs cross-talk between IFNγ and TLR4 resulted in augmented STAT1 phosphorylation and increased expression of the chemokine CXCL10 and the adhesion molecule ICAM-1 as well as adhesion of U937 leukemia cells to ECs, in a STAT1- and TLR4-dependent manner [17]. We hypothesize that STAT1-dependent transcriptional changes in vascular cells involved in cross-talk between IFNγ and TLR4, reflect pro-atherogenic responses in human atherosclerosis.

Our study indeed provides evidence that in ECs and VSMCs STAT1 coordinates a platform for cross-talk between IFNγ and TLR4, and identifies a STAT1-dependent gene signature that reflects a pro-atherogenic state in coronary artery disease (CAD) and carotid atherosclerosis.

## Materials and Methods

### Cell culture experiments

This investigation conforms with the principles of the NIH Guide for the Care and Use of Laboratory Animals (NIH Publication, 8th Edition, 2011) and the German Law on the Protection of Animals was followed. Researchers in charge of the experiment, at Klinikum rechts der Isar were authorized to breed, house, and sacrifice animals. WT mice (strain background *C57BL/6*) were obtained from Charles River Laboratories. *STAT1^−/−^* and *IRF8^−/−^* mice (both *C57BL/6* background) were kindly provided by Thomas Decker and Carol Stocking, respectively [18]. Before any manipulations, animals were euthanized by cervical dislocation under isoflurane anesthesia. Primary murine Vascular Smooth Muscle cells (VSMCs) were isolated from *C57BL/6* or *STAT1^−/−^* or *IRF8^−/−^* aortas by enzymatic digestion [19]. Human Microvascular Endothelial Cells (ECs) [20] obtained from Centers for disease control and prevention that were used in current study, were cultivated in MCDB-131 (Life Technologies) medium containing 10% FBS (PAA), 100 U/ml penicillin, 100 µg/ml streptomycin, 0.01 µg/ml EGF, 0.05 µM hydrocortisone (Sigma), 2 mM L-glutamine (PAA). On the day before the experiment for both cell types full medium was exchanged into medium containing 2% serum. Afterwards, cells were treated with 10 ng/ml of IFNγ (Life Technologies, PMC4031) and/or 1 µg/ml of LPS (Sigma, L4391).

### RNA isolation and real-time PCR

**Figure 1:**
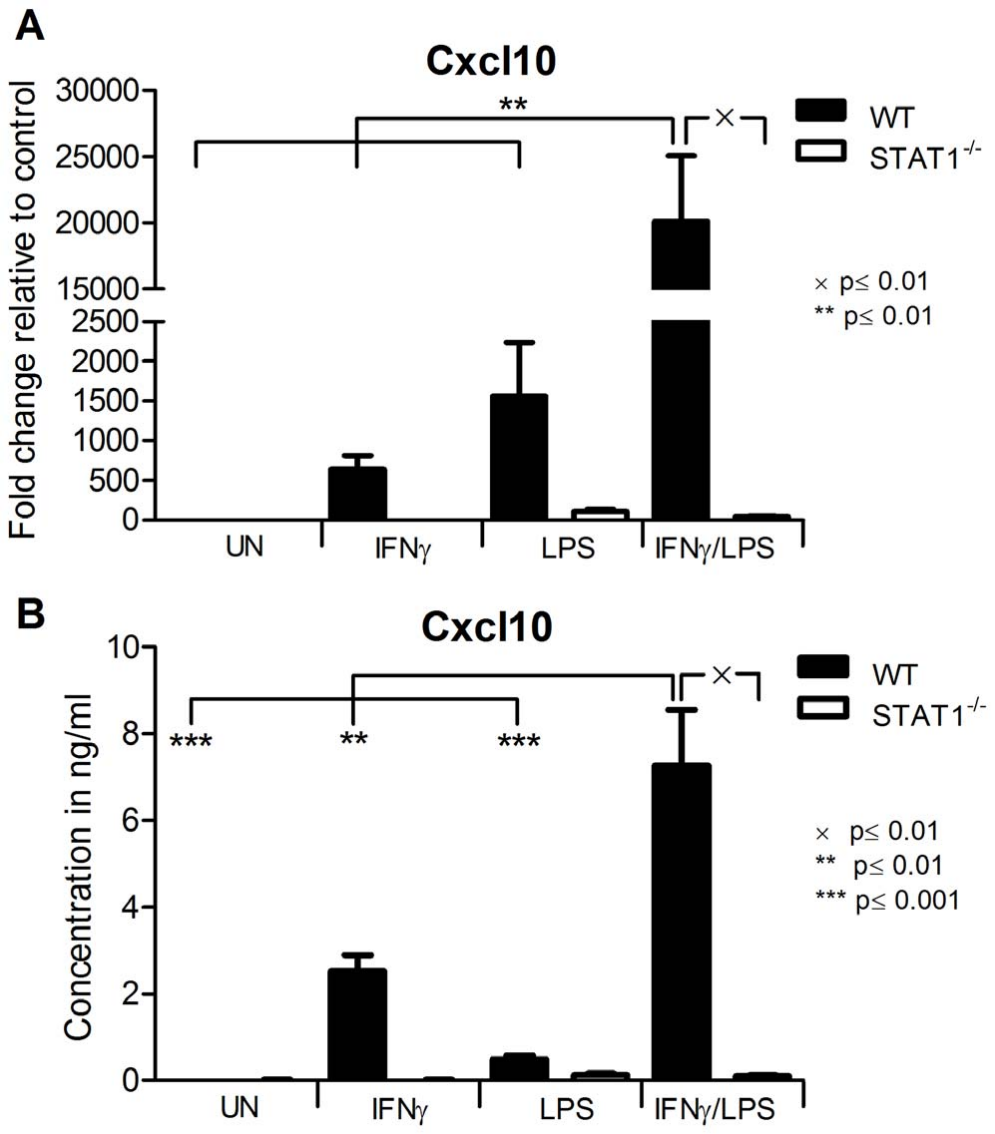
CXCL10 amplified by IFNγ and LPS in VSMCs is STAT1 dependent. A, *WT* and *STAT1^−/−^* VSMCs were treated with 10 ng/ml IFNγ for 8 h or with 1 ug/ml of LPS for 4 h or with IFNγ for 4 h followed by LPS for additional 4 h. RNA was isolated and qRT-PCR for *Cxcl10* using *Gapdh* as internal control was performed. B, Cells were treated as in A. On the medium remained after treatment ELISA for CXCL10 was performed. Data represent means of at least 3 independent biological experiments ±SEM and p<0.05 was considered as significant. Data were tested for significance by one-way ANOVA followed by post-hoc Tukey or unpaired two-tailed student T-test when appropriate.

Total RNA was isolated from VSMCs and ECs using RNAeasy Mini Kit (Qiagen, 74104) together with DNAse digestion step according to the manufacture's protocol. Isolated aortas were cleaned from perivascular fat and incubated as depicted in Fig. 1. After stimulation aortas were snap frozen on liquid nitrogen, ground up with a pestle and resuspended in 1 ml of Trizol. Total RNA was isolated using Trizol method followed by PureLink RNA kit (Life Technologies, 12183018A). Complementary DNA was synthesized using iScript cDNA Synthesis Kit (BioRad, 170-881), according to manufacturer's protocol. Quantitative reverse transcriptase PCR (qRT-PCR) was performed using SSoFast Evagreen (MyiQ ICycler, Bio-Rad, 172-5201). Forward and reverse primers are depicted in Table S4. The 2^−ddCt^ method was applied for quantification [21]. Fold change in the target gene were normalized to *GAPDH* and relative to the mean expression at untreated sample. The results are expressed as fold of control from at least 3 independent assays.

### Microarray analysis

VSMCs from *WT* and *STAT1^−/−^* were treated as described in Fig. 1. RNA from control and treated samples was isolated and labeled according to Illumina TotalPrep RNA Amplification Kit (Life Technologies, AMIL1791). Standard Illumina Expression BeadChip MouseRef-8v2 (Illumina) hybridization protocol was used to obtain the raw data. Chips were scanned using HiScanSQ system. The complete data of the Illumina Expresion BeadChip analysis can be found at the NCBI GEO, with the accession number GSE49519. The average signals from 3 independent biological experiments were taken for statistical testing. Genes from treated samples with detection p-value <0.05 were selected for background subtraction and quantile normalization. Up-regulated genes were considered with p-value <0.05 and at least 2-fold difference. Genes which expression after co-treatment reached higher level than additive expression after IFNγ or LPS were considered as amplified. Regulated genes in WT cells which expression was lowered at least by 50% or fold induction was smaller than 2 in STAT1^−/−^ was considered as a STAT1 target. For comparison of up-regulated genes Venn diagram tool was used (http://bioinfogp.cnb.csic.es/tools/venny/index.html) [22]. Gene names from data sets were used for identifying overlapping genes. Promoters for amplified STAT1 dependent genes were screened using GENOMATIX software (http://www.genomatix.de/) [23]. The promoter regions from −1000 to +100 bp were searched for binding sites (V$IRF1.01 V$ISGF3G.01 V$ISRE.01 V$ISRE.02V$CREL.01 V$NFKAPPAB.01 V$NFKAPPAB.02 V$NFKAPPAB65.01 V$STAT.01 V$STAT1.01 V$STAT1.02) or models with core similarity at least 0.85. Enrichment in gene ontology processes categories was performed using Gorilla software (http://cbl-gorilla.cs.technion.ac.il/) [24]. P-value of 10^−3^ was used as a threshold and Illumina gene lists from HumanHT-12 v4 or MouseRef-8 v2 were taken as a background model. Next, all the statistically significant and enriched gene ontology categories were analyzed by Revigo software (http://revigo.irb.hr/) [25]. To remove redundant GO terms the allowed similarity value of 0.5 was used.

### *In silico* gene expression analysis

Human atherosclerotic plaque datasets were downloaded from NCBI Gene Expression Omnibus repository. Carotid dataset (accession no. GSE21545 [26] contained 223 microarrays (124 samples were used for the analysis) and coronary dataset (accession no. GSE40231 [27] contained 278 samples (80 arrays were used for the analysis)). As GSE21545 did not contain any healthy artery controls, these samples were compared against controls from GSE40231. For this purpose, batch effects between the combined datasets were removed using ComBat tool, a widely used method for removing variations between batches of arrays [28]. In both cases the authors isolated RNA from whole plaques obtained from patients during surgery.

Raw.cel files downloaded from GEO were normalized using RMA algorithm, signals were log-transformed and probes were combined to genes using “Combine probes to genes” tool (Chipster software [29]). Fold change and corresponding p-values were calculated using “calculate fold change” tool (Chipster [29]). Genes up-regulated at least 1.5 times in both datasets were compared with a list of 30 genes amplified by IFNγ and LPS treatment in VSMCs.

The list of STAT1 target genes up-regulated by IFNγ and LPS in murine VSMCs was used as the starting point for promoter analysis. First, that list was fed to pSCAN online promoter analysis tool in order to look for GAS, interferon stimulated response element ISRE (recognized by IRFs) and NFκB binding sites. The software was set to analyse 950 bp upstream and 50 bp downstream of the transcription start site. PSCAN produced a list of over-represented transcription factor binding sites together with occurrences of each site and a matrix similarity score. Occurrences having the score of at least 0.8 were fed into MatDefine (Genomatix software package) to create a highly conserved matrix for each transcription factor binding site. The settings were as follows: tuple size – 8; no. of sequences containing tuple – 60%; matrix similarity score for sequence inclusion – 0.9. Matrices for GAS, ISRE and NFκB binding sites were then used in pSCAN as «user supplied matrices» to search for occurrences in genes two-fold upregulated in the atherosclerotic plaque datasets.

### ChIP-qPCR

ChIP was performed as previously described [30] with minor modifications. Briefly, VSMCs were stimulated as depicted in Fig. 1 and next crosslinked with 1% formaldehyde for 10 minutes. After fixation chromatin was sonicated with a Diagenode Bioraptor to generate 200–1000 bp fragments. Chromatin was immunoprecipitated with pre-immune IgG (Millipore, 12–371B), or with an antibody against STAT1 (Santa Cruz, sc-346) or IRF1 (Santa Cruz, sc-13041X) or NFκB (Santa Cruz, sc-398442X). Chromatin-antibody complexes were precipitated with agarose beads according to the EZ ChIP protocol (Millipore, 17-371). After DNA fragments were column purified DNA concentration was measured with a Qubit fluorometer. Immunoprecipitated DNA was quantified by qPCR (primers for *Cxcl10*: 5′-CCTGTAAACCGAGGGCATTG-3′, 5′-CACGCTTTGGAAAGTGAAAC-3′) and normalized to values obtained after amplification of unprecipitated (input) DNA.

### Western blot analysis

Total IRF8, STAT1 (Santa Cruz, sc6058, sc346), GAPDH and phosphorylated STAT1 (Cell Signaling, 5174s, 9171l) were determined by western blotting in VSMCs and HMECs. After treatment cells were homogenized in a Ripa lysis buffer (Sigma) containing phosphatases and proteases inhibitors (Roche). Protein concentration was determined using a bicinchoninic acid protein assay kit (Thermo Fisher Scientific). 40 µg of protein per lane was loaded and resolved by SDS-poly-acrylamide gel electrophoresis (PAGE) under reducing conditions. Proteins were transferred onto PVDV (Millipore) membrane. After incubation with primary and horseradish peroxidase-conjugated secondary antibodies (Santa Cruz), immunoreactivity was detected by adding Luminata Forte Western Substrate (EMD Millipore) and measured by INTAS imaging system (Intas, Germany).

### Cytokine detection ELISA

Expression of murine Cxcl10, Ccl5 (Peprotech) as well as Cxcl9 (Sigma) was performed on medium remained after treatment of VSMCs using sandwich ELISA tests according to the manufacturer's instructions.

### Measurement of nitric oxide (NO)

VSMCs were treated as depicted in cell experiment section. After treatment medium was refreshed and cells were cultivated for further 24 h. Subsequently medium was collected and 100 ul was used to measure amount of NO by Griess diazotization reaction [31]. Medium was incubated with freshly prepared solution containing 1% sulfanilamide 5% HCl, 0.1% aqueous solution of 2-(1-Naphthylamino)ethylamine dihydrochloride (Sigma). After 10 min incubation OD at 560 mm was measured and compared to the standard curve.

### Migration assay

Migration assay was performed according to Guo et al [32]. Briefly, 10^6^ of isolated red blood cells depleted splenocytes isolated from WT mice, were loaded into the upper chamber of Transwell 24-well plates (Corning, 3421). The bottom chamber was filled with 600 ul of the medium collected after treatment of VSMCs with LPS, IFNγ or IFNγ and LPS. After incubation for 3 h at 37°C, migrated cells were stained with CD45FITC and CD3APC antibody (Miltenyi Biotec 130091609, 130092977) and analyzed by flow cytometer (Miltenyi Biotec).

### *Ex vivo* contractility studies

Isolated aortas were cleaned from perivascular fat, cut into 2 mm long rings (for myograph) and placed in a DMEM medium containing 2% FBS (Sigma). Next, aortas were treated with 10 ng/ml of IFNγ and/or 1 µg/ml of LPS. Vascular contractility studies were performed according to the technique described by Mulvany et al. [33]. After treatment, 2 mm long rings were mounted in a 4-channel myograph (620M, Danish Myo Technology, Aarhus, Denmark) in the organ chamber filled with physiological saline solution (PSS) containing 118.99 mM NaCl, 4,69 mM KCl, 1.17 mM MgSO_4_*7H_2_O, 1.18 mM KH_2_PO_4_, 2.5 mM CaCl_2_*2H_2_O, 25 mM NaHCO_3_, 0.03 mM EDTA, 5.5 mM Glucose. During the experiment PSS buffer was aerated with carbogen (95% O_2_+5%CO_2_). After calibration, vessels were pre-streched to obtain optimal passive tension. Next, vascular functions were analyzed. Contractility was evaluated by substitution of PSS buffer for high potassium physiological saline solution (KPSS; 74.7 mM NaCl, 60 mM KCl, 1.17 mM MgSO_4_*7H_2_O, 1.18 mM KH_2_PO_4_, 1.6 mM CaCl_2_, 14.9 mM NaHCO_3_, 0.026 mM EDTA, 5.5 mM Glucose). For testing viability, vessels were subjected to noradrenaline-induced constriction followed by acetylcholine dilation (Sigma). After washing out with PSS buffer and resting for 15 minutes, noradrenaline dose-response curves was performed. Noradrenaline was used in stepwise increased, cumulative concentration ranging from 10^−11^ to 10^−6^ mol/L. To study vasodilatation, sodium nitroprusside (Sigma) was used in concentrations from 10^−10^ to 10^−5^ mol/L.

### Histology and immunohistochemistry

Histological analyses and immunohistochemistry were performed on representative sections (2–3 µm) of formalin fixed in paraffin embedded tissue samples from six human carotid atherosclerotic lesions and four healthy controls. The human tissue samples used in our study were procured from Biobank of Department of Vascular and Endovascular Surgery (Klinikum rechts der Isar der Technischen Universitaet Muenchen). Collecting of specimens for the mentioned Biobank was approved by the local ethics committee (Ethikkommission der Fakultaet fuer Medizin der Technischen Universitaet Muenchen) and written informed consent was given by all patients. Haemalaun-Eosin (HE) and Elastica-van-Gieson (EvG) staining were performed in order to assess sample morphology. For characterisation of the cells within atherosclerotic plaques, specimens were treated with antibodies against vascular smooth muscle cells (smooth muscle myosin heavy chain 1 and 2 (SM-M10), rabbit polyclonal, dilution 1∶4.000 (Abcam, ab81031) and endothelial cells (anti-CD31, mouse monoclonal, clone JC70A, dilution 1∶100; Dako).

For the detection of specific cytokines, CXCL9 (MIG) and CXCL10 (IP10), as well as the phosphorylated transcription factor STAT1, following primary antibodies were used: rabbit polyclonal anti-MIG (Abcam, ab9720; dilution 1∶500), rabbit polyclonal anti-IP10 (Abcam, ab47045; dilution 1∶200), and rabbit monoclonal phospho-Stat1 (Cell Signaling, 9171l; dilution 1∶400). All antibodies were first optimised on tonsil using different dilutions, staining conditions and with or without blocking. Optimal results were achieved by blocking anti-MIG and anti-phospho-Stat1 with goat serum, anti-IP10 without the blocking procedure.

Following incubation with primary antibody visualisation was performed by peroxidase/DAB ChemMate Detection Kit according to the manufacturer's instruction (biotinylated goat anti-mouse/anti-rabbit secondary Ab; Dako).

### Statistical Analysis

Data are presented as mean ± SEM. For comparisons between more than two groups one-way ANOVA with Tukey post-hoc test was used. In all other experiments comparing two groups, Student's t-test was used. A probability value <0.05 was considered statistically significant (GraphPad Prism 5.0). In contractility studies, two-way ANOVA test with Bonferroni post hoc test was used.

## Results

### IFNγ and LPS synergistically induce CXCL10 expression in VSMCs, depending on STAT1

Recently, we showed that in ECs cross-talk between IFNγ and TLR4 resulted in augmented STAT1 phosphorylation and increased expression of the chemokine CXCL10 [17]. To study if a similar mechanism affected the expression of *Cxcl10* in VSMCs, these cells were isolated from *WT* and *STAT1^−/−^* mice and treated as depicted in Fig. 1. In *WT-VSMCs*, treatment with IFNγ or LPS alone induced expression of *Cxcl10* at the mRNA (Fig. 1A) as well as at the protein level (Fig. 1B). Furthermore, pre-treatment with IFNγ for 4 h followed by LPS for another 4 h led to synergistic amplification of *Cxcl10* expression compared with both factors alone (Fig. 1A and 1B). In contrast, this IFNγ and LPS-induced synergistic amplification in *Cxcl10* gene expression was dramatically abrogated in *STAT1^−/−^-VSMCs* (Fig. 1A), which coincided with Cxcl10 protein levels in the medium (Fig. 1B) and correlated with a predominant STAT1-dependent mechanism.

### Transcriptional responses in IFNγ and LPS treated VSMCs predict dependence on STAT1, NFκB and IRF

**Figure 2:**
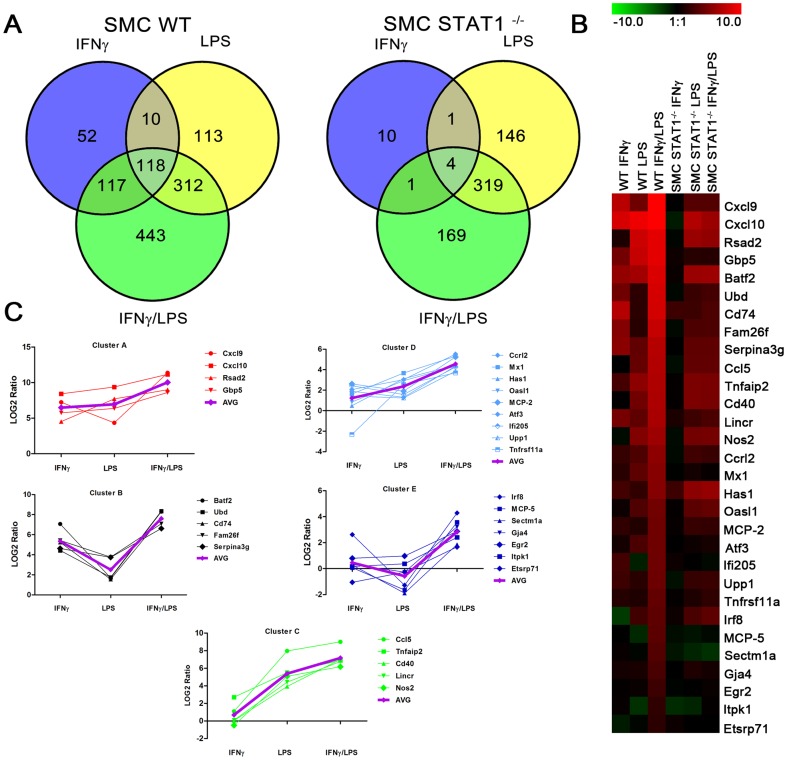
Identification of genes prone to synergistic amplification upon treatment with IFNγ and LPS. *WT* and *STAT1^−/−^* VSMCs were treated as described in Fig. 1. On RNA isolated from untreated or IFNγ, LPS or IFNγ+LPS treated VSMCs genome-wide expression profiling was performed. A, Venn diagrams revealing number of differentially expressed genes upon stimulation. B, Heat map of the expression of synergistically amplified genes in *WT* and *STAT1^−/−^VSMCs*. C, Clustering of the synergistically upregulated genes according to their expression profile. AVG, average expression in the group. For details see text.

Next, we compared genome-wide transcriptional responses of *WT-VSMCs* to LPS (4 h) or IFNγ (8 h) alone, or after combined treatment (IFNγ 8 h, LPS 4 h). IFNγ changed the expression of 297 and LPS of 553 genes under these conditions (Fig. 2A). The interactions between IFNγ and LPS (Fig. 2A) increased the number of up-regulated genes to 990. While 128 of the IFNγ-regulated genes were modulated by LPS (Fig. 2A), 118 were also commonly regulated by IFNγ+LPS. Likewise, we compared transcriptional responses of *STAT1^−/−^-VSMCs* to LPS or IFNγ alone, or after combined treatment. Only 16 genes were found to be up-regulated by IFNγ in *STAT1^−/−^-VSMCs*, highlighting the importance of STAT1 in this response pathway. In contrast, LPS treatment of *STAT1^−/−^-VSMCs* was similar to *WT-VSMCs*, with a total of 470 genes being modulated. However, in general the potency of the response was lower as compared to *WT-VSMCs*. Consequently, the additive or synergistic effect of IFNγ and LPS as seen in *WT-VSMCs*, was no longer present in *STAT1^−/−^-VSMCs*. Only 493 genes were upregulated by IFNγ+LPS, of which 323 were in common with LPS alone. The complete list of up and down-regulated genes in response to IFNγ or LPS alone, or after combined treatment is shown in Table S1, S2 and S3, respectively.

Subsequently, we aimed at identifying the genes, that similar to *Cxcl10* were synergistically affected by the interactions between IFNγ and LPS, and their dependency on STAT1. Table 1 shows the top 30 synergistically amplified genes of which the expression was at least 2-fold higher upon stimulation with IFNγ+LPS as compared to the sum of the treatments with both factors alone. For example, expression of *Cxcl9* was >15-fold higher after combined treatment [2643.5-fold increased by IFNγ+LPS, divided by 171 times as the sum of IFNγ (150.73) and LPS (20.25) alone] as compared to the single treatments.

**Table 1:** Genes prone to synergistic amplification upon stimulation with IFNγ and/or LPS and their promoter analysis.

SYMBOL	WT IFNγ	WT LPS	Signal integration	WT IFNγ+LPS	STAT1^−/−^IFNγ	STAT1^−/−^LPS	STAT1^−/−^IFNγ+LPS	STAT_NFκB	IRF_NFkB	ISRE	STAT	NFκB	Cluster
Cxcl9	150.73	20.25	15.46	2643.50	0.93	9.87	9.52	x	-	-	-	-	A
Cxcl10	343.62	665.68	2.25	2273.44	0.47	119.63	66.53	x	x	-	-	-	
Rsad2	22.91	209.45	2.19	509.05	1.53	2.93	2.71	-	-	X	x	x	
Gbp5	53.48	82.66	2.85	388.28	1.28	65.64	72.68	x	-	X	x	x	
Batf2	134.37	3.23	2.29	314.46	5.35	5.10	8.31	x	-	X	x	x	B
Ubd	21.54	3.38	13.11	326.57	0.80	4.71	6.44	x	-	X	-	-	
Cd74	37.66	2.93	5.09	206.50	0.78	8.01	8.24	x	x	-	-	-	
Fam26f	43.02	13.60	2.42	137.16	0.95	13.25	13.07	-	-	X	x	-	
Serpina3g	24.76	13.36	2.59	98.56	2.12	5.12	7.79	x	-	X	-	-	
Ccl5	2.14	249.59	2.04	512.54	0.83	64.48	52.22	x	x	-	-	-	C
Tnfaip2	6.52	45.55	2.27	118.00	1.14	28.83	27.04	x	-	-	-	-	
Cd40	1.03	15.21	7.83	127.14	0.70	14.09	14.14	-	-	X	x	x	
Lincr	1.07	21.38	4.84	108.75	1.16	29.33	25.78	-	-	X	x	x	
Nos2	0.71	33.99	2.08	72.15	0.73	22.52	24.13	x	x	-	-	-	
Ccrl2	4.18	8.16	3.72	45.93	0.76	5.87	4.65	x	-	X	-	-	D
Mx1	3.10	12.73	2.49	39.41	0.91	1.68	1.18	x	-	X	-	-	
Has1	1.40	8.71	2.16	21.84	1.17	11.64	14.92	-	-	-	x	x	
Oasl1	1.80	7.61	2.13	20.10	1.03	1.45	1.83	-	-	X	-	x	
MCP-2	6.12	3.75	3.83	37.77	5.21	44.53	58.31	-	-	-	x	-	
Atf3	3.73	2.47	3.27	20.29	1.21	4.34	3.96	-	-	-	x	x	
Ifi205	5.39	2.99	2.28	19.10	0.64	4.24	4.81	-	-	X	x	-	
Upp1	2.67	2.37	2.87	14.45	1.17	2.25	3.53	x	-	X	-	-	
Tnfrsf11a	0.20	5.87	2.12	12.85	1.46	7.44	12.33	x	-	-	-	-	
Irf8	6.13	0.41	2.98	19.51	1.40	1.03	0.77	x	-	-	-	-	E
MCP-5	1.05	0.32	8.65	11.86	0.69	0.59	0.78	-	-	x	x	x	
Sectm1a	1.21	0.27	2.45	3.62	0.30	0.37	1.16	x	-	x	-	-	
Gja4	1.25	0.83	4.50	9.35	0.55	0.37	0.27	-	-	-	-	x	
Egr2	1.74	1.96	1.98	7.32	0.98	2.31	1.73	x	-	-	-	-	
Itpk1	1.12	1.29	2.19	5.29	0.86	0.97	1.30	-	-	-	x	x	
Etsrp71	0.48	0.83	2.43	3.18	1.39	0.96	0.96	-	-	-	x	x	

The table introduces genes that expression is at least 2-fold higher upon stimulation with IFNγ+LPS as compared to the sum of the treatments with both factors alone (see column “Signal integration”). Other numbers represent fold changes compared to control. Cross indicates presence of specific sequence in the promoter regions.

Subsequently, by grouping these 30 genes based on their response pattern in *WT-VSMCs* to IFNγ or LPS (Fig. 2B), we could distinguish five groups of genes (Fig. 2C). The first group contained genes with high response to both IFNγ and LPS that were highly amplified after combined treatment (Cluster A in Table 1 and Fig. 2C). These include *Cxcl9, Cxcl10, Rsad2 and Gbp5*. The expression of a second group of genes, including *Batf2*, *Ubd*, *Cd74*, *Fam26f* and *Serpina3g*, showed high response to IFNγ, mild response to LPS, and high amplification after combined treatment (Cluster B in Table 1 and Fig. 2C). In contrast, a third group of genes showed a mild or no response to IFNγ, high response to LPS, and again high amplification after combined treatment (Cluster C in Table 1 and Fig. 2C). This group was exemplified by *Ccl5*, *Tnfaip2*, *Cd40*, *Lincr* and *Nos2 (iNOS)*. The fourth group of genes consisted of C*crl2*, *Mx1*, *Has1*, *Oasl1*, *MCP-2*, *Atf3*, *Ifi205*, *Upp1* and *Tnfrsf11a* and displayed mild or no response to IFNγ, mild response to LPS, and mild amplification after combined treatment (Cluster D in Table 1 and Fig. 2C). Finally, we could also identify genes which showed minor or no response to IFNγ and LPS alone, but were highly amplified in expression after combined treatment [e.g., *Irf8, MCP-5, Sectm1a, Gja4, Egr2, Itpk1* and *Etsrp71*] (Cluster E in Table 1 and Fig. 2C).

In general, the absence of STAT1 severely abrogated the IFNγ-induced expression of all of these 30 genes. On the other hand, the LPS response of 50% of genes listed in Table 1, was decreased in the absence of STAT1. Consequently, the synergistic effect of IFNγ and LPS as seen in *WT-VSMCs*, was no longer present in *STAT1^−/−^-VSMCs*. As the only exception, the IFNγ-induced expression of *MCP-2* appeared STAT1-independent, with a similar fold induction in WT and STAT1^−/−^ VSMCs (Table 1, 6.12 vs. 5.12). In contrast, absence of STAT1 increased its response to LPS (Table 1, 3.75 vs. 44.53).

Successive promoter analysis of the genes listed in Table 1, predicted the presence of STAT-NFκB and IRF-NFκB modules or combinations of separate ISRE, STAT or NFκB binding sites, strongly implicating the cooperative involvement of NFκB, STAT1 and/or IRFs in the transcriptional regulation of all of these genes in response to IFNγ and LPS.

### Transcriptional responses in IFNγ and LPS treated VSMCs and ECs predict a pro-atherogenic phenotype

Gene ontology (GO) functional analysis of the top 30 genes listed in Table 1, revealed significant enrichment in biological functions involved in host defense, immune response, inflammatory response, cytokine response, response to stress and to wound healing (Table 2). All these categories generally recognize a similar group of genes, including the chemokines *Cxcl9, Ccl12, Ccl8, Ccl5, Cxcl10* and *Ccrl2*, adhesion molecules (*Cd40, Cd74*), and the antiviral and antibacterial response genes *Irf8*, *Rsad2*, *Mx1*, *Oasl1*, *Gbp5*, *Nos2*, *Batf2* and *Tnfrsf11a*. Together, these genes reflect an enhanced pro-inflammatory and pro-atherogenic profile that is mediated by interactions between IFNγ and LPS in VSMCs and strongly depends on STAT1.

**Table 2:** Gene ontology classification of synergistically amplified genes.

Term ID	Description	frequency	log_10_ p-value	Uniqueness	Dispensability
GO:0051707	response to other organism	0.01	−10.10	0.56	0.00
GO:0009607	response to biotic stimulus	0.01	−9.62	0.66	0.40
GO:0006952	defense response	0.01	−8.91	0.63	0.41
GO:0002376	immune system process	0.01	−7.61	0.97	0.00
GO:0071345	cellular response to cytokine stimulus	0.00	−6.87	0.52	0.32
GO:0006950	response to stress	0.04	−6.59	0.61	0.50
GO:0006955	immune response	0.01	−6.26	0.41	0.39
GO:0006954	inflammatory response	0.00	−5.91	0.68	0.49
GO:0045071	negative regulation of viral genome replication	0.00	−5.04	0.76	0.47
GO:0009611	response to wounding	0.00	−4.93	0.68	0.53

**Figure 3:**
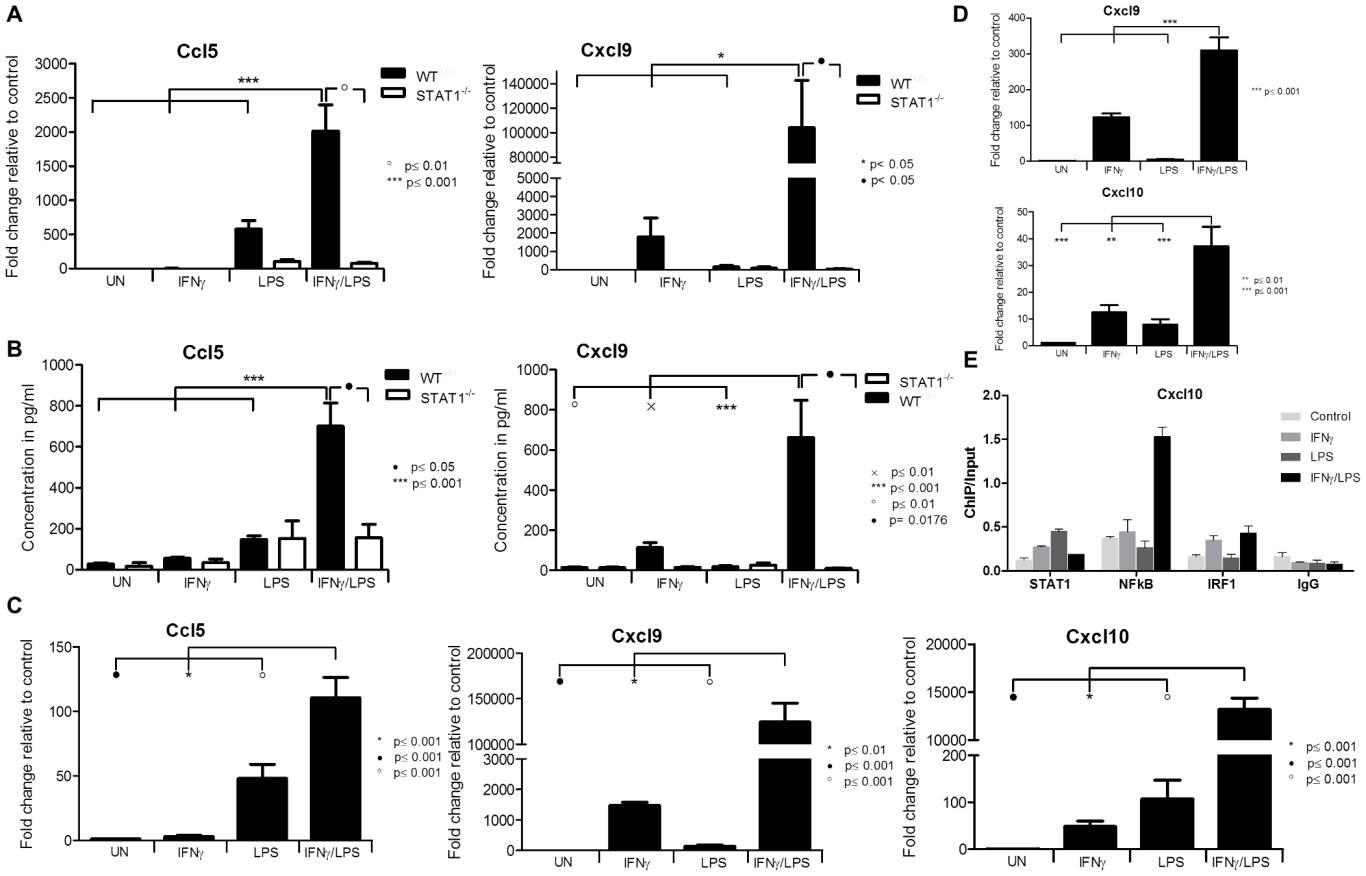
Effect of STAT1 dependent signal integration on chemokine expression. *WT* and *STAT1^−/−^ VSMCs*, HMECs or *WT* aortic ring segments were treated as described in Fig. 1. A, RNA from VSMCs was isolated and qRT-PCR for *Ccl5*, *Cxcl9* using *Gapdh* as internal control was performed. B, On the medium remained after treatment of VSMCs ELISA for Ccl5 and Cxcl9 was performed. C, Expression of *CXCL10, CXCL9 and CCL5* upon stimulation in ECs. D, RNA from incubated aortic rings was isolated and qRT-PCR for *Cxcl10*, *Cxcl9* using *Gapdh* as internal control was performed. Data represent means of at least 3 independent biological experiments ±SEM and p<0.05 was considered as significant. Data were tested for significance by one-way ANOVA followed by post-hoc Tukey or unpaired two-tailed student T-test when appropriate. E, ChIP-qPCR analysis of the *Cxcl10* promoter region containing NFκB and ISRE binding sites show the enrichment with STAT1, NFκB and IRF1 antibodies compared with IgG control in an IFNγ, LPS or IFNy+LPS-dependent manner in *WT VSMCs*. Immunoprecipitated DNA was quantified by qPCR and normalized to values obtained after amplification of unprecipitated (input) DNA. A representative experiment is shown.

The expression of the chemokines *Ccl5*, *Cxcl9*, *Ccl12* and chemokine receptor *Ccrl2* (not shown) was additionally examined by qPCR and ELISA (Fig. 3), and confirmed the microarray data. In agreement with Table 1, the response of this selected group of genes was severely abolished in *STAT1^−/−^-VSMCs*, confirming the importance of STAT1 in the signal integration between IFNγ and LPS. Because we were not able to isolate a homogeneous population of mouse aortic endothelial cells (data not shown), we instead used the human microvascular endothelial cell-line, HMEC [20]. Pre-treatment of HMECs with IFNγ for 4 h followed by LPS for another 4 h resulted in a similar amplification pattern of *Ccl5*, *Cxcl9* and *Cxcl10* (Fig. 3C) as in *WT-VSMCs*, providing evidence for a universal STAT1-dependent mechanism in vascular cells triggered by IFNγ and LPS. Similarly, we were able to observe a synergistic amplification after IFNγ and LPS treatment of *Cxcl9* and *Cxcl10* in *ex vivo* treated aortic rings of WT animals as compared to IFNγ or LPS alone (Fig. 3D)

Finally, Chromatin-immunoprecipitation (ChIP)-qPCR of untreated *WT-VSMCs* or treated with IFNγ, LPS or IFNγ+LPS and using antibodies against STAT1 NFκB, IRF1 or IgG, clearly showed enhanced binding of these different transcription factors to the ISRE and NFκB binding elements of the *Cxcl10* gene, as compared to IgG controls (Fig. 3E). In a representative experiment, STAT1 binding to the ISRE increased after IFNγ as well as LPS treatment, but not after IFNγ+LPS stimulation. IRF1 binding was enriched upon treatment with IFNγ alone and after subsequent stimulation with LPS. LPS alone, on the other hand did not affect IRF1 binding. Finally, NFκB binding dramatically increased when cells were first treated with IFNγ and then by LPS, but not in the presence of IFNγ or LPS alone (Fig. 3E). This confirms the cooperative involvement of STAT1, NFκB and IRF1 in the transcriptional regulation of *Cxcl10* in response to IFNγ and LPS as predicted in Table 1.

### IRF8 mediates IFNγ and LPS induced *Ccl5* expression in vascular cells

**Figure 4:**
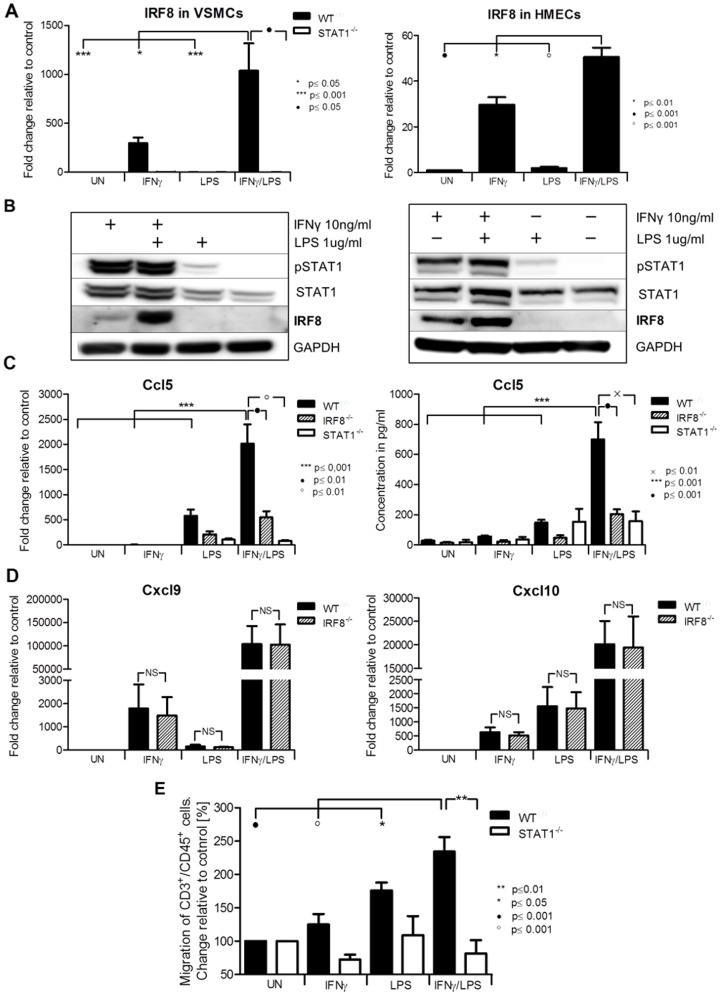
IRF8 mediated cross-talk and functional activity of synergistically amplified chemokines. *WT, STAT1^−/−^ and IRF8^−/−^* VSMCs and HMECs were treated as described in Fig. 1. A, RNA was isolated and qRT-PCR for *IRF8* using *GAPDH* as internal control was performed in VSMCs (left panel) and ECs (right panel). B, Protein extracts were analyzed for IRF8, tyrosine-phosphorylated STAT1, total STAT1 and GAPDH. C, *CCL5* mRNA expression (left panel) and protein presence in the medium (right panel) was measured. D, Expression profiles of *Cxcl9* (left panel) and *Cxcl10* (right panel) between VSMCs *WT*, and *IRF8^−/−^* were compared. E, Migration assay of CD45^+^/CD3^+^ performed on conditioned medium remained after treatment of VSMCs *WT* and *STAT1^−/^* ^−^. Data represent means of at least 3 independent biological experiments ±SEM and p<0.05 was considered as significant. Data were tested for significance by one-way ANOVA followed by post-hoc Tukey or unpaired two-tailed student T-test when appropriate.

Notably, the transcription factor IRF8, which was thought to be restricted to lymphoid-cell lineages such as B-, T-, dendritic cells and macrophages, was identified among the amplified genes. Indeed, gene (Fig. 4A left panel) and protein expression (Fig. 4B left panel) of IRF8 in *WT* and *STAT1^−/−^* VSMCs in response to IFNγ, LPS or IFNγ+LPS, confirmed the microarray data. Interestingly, pre-treatment of ECs with IFNγ for 4 h followed by LPS for another 4 h resulted in a similar amplification pattern of *IRF8* RNA (Fig. 4A right panel) and protein expression (Fig. 4B right panel) as in *WT-VSMCs*. These results provide evidence for STAT1-dependent expression of IRF8 in VSMCs and ECs upon treatment with IFNγ, and confirm amplification of IRF8 upon stimulation with IFNγ and LPS in vascular cells.

Next the IRF8 dependent regulation of *Ccl5* (a known IRF8 target in immune cells [34]) was examined. The amplified expression of *Ccl5* RNA (Fig. 4C, left panel) and protein (Fig. 4C, right panel) in response to IFNγ and LPS, as seen in *WT* VSMCs, was highly attenuated in *IRF8^−/−^* and *STAT1^−/−^ -VSMC*. In contrast, the expression of *Cxcl10* and *Cxcl9* in response to IFNγ and LPS in *WT VSMCs* was similar to that in *IRF8^−/−^-VSMCs* (Fig. 4D).

### Signal integration between IFNγ and LPS in VSMCs leads to increased migration of T-lymphocytes

Since many of the chemokines characterized above are involved in chemotaxis of T-lymphocytes [35], we examined the effect of IFNγ and LPS cross-talk on T-cell migration towards conditioned medium from treated VSMCs. Amplification of chemokines in *WT-VSMCs* upon stimulation with both stimuli indeed led to an increased migration of CD3^+^/CD45^+^ spleenocytes (Fig. 4E). Migration of CD3^+^/CD45^+^ cells towards medium of *WT-VSMCs* treated with both IFNγ and LPS was significantly higher (234%) as compared to both factors alone (125% and 175%, respectively). As expected, the chemotactic response of splenocytes towards the conditioned medium obtained after treatment of *STAT1^−/−^-VSMCs* was highly attenuated (Fig. 4E).

### Signal integration between IFNγ and LPS in aortic rings leads to abolished response to norepinephrine and sodium nitroprusside

**Figure 5:**
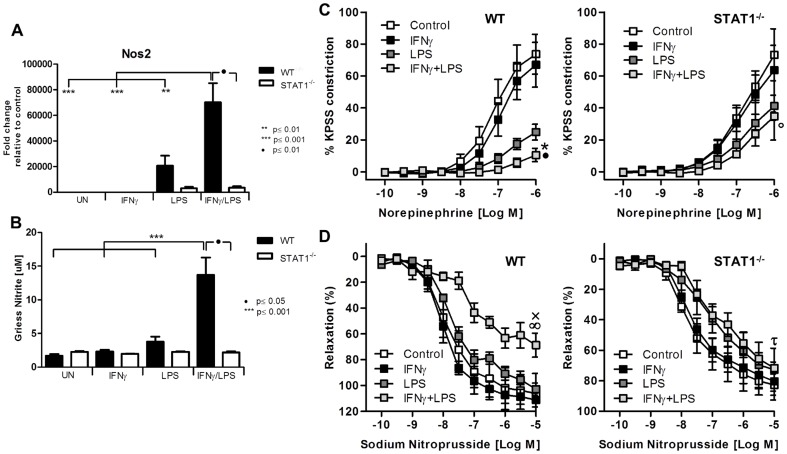
STAT1-mediated abolished response to norepinephrine and sodium nitroprusside is associated with disturbed NO production. A, *WT* and *STAT1^−/−^ VSMCs* were treated as described in Fig. 1. RNA was isolated and qRT-PCR for *Nos2* using *Gapdh* as internal control was performed (upper panel) B, After stimulation as described in Fig. 1, medium was refreshed and left for 24 h. Next, 100 µl of the medium was taken and the product of Nos2- nitrite was measured. Data represent means of at least 3 independent biological experiments ±SEM and p<0.05 was considered as significant. Data were tested for significance by one-way ANOVA followed by post-hoc Tukey or unpaired two-tailed student T-test when appropriate. C, D Isolated aortic rings from WT and STAT^−/−^ mice were incubated with 10 ng/ml IFNγ for 8 h or with 1 ug/ml of LPS for 4 h or with IFNγ for 4 h followed by LPS for additional 4 h. Next, response to norepinephrine and sodium nitroprusside was tested on the wire myograph. C, Response to noradrenaline in WT and STAT1-deficient aortic rings presented as a percentage of maximal constriction to KPSS.*p<0.001 vs. WT control; •p<0.001 vs. WT LPS; ○p<0.001 vs. STAT1^−/−^ control. D, Response to stepwise increased concentration of sodium nitroprusside. xp<0.05 vs. WT control; ∞p<0.01 vs. WT LPS; □p<0.05 STAT1^−/−^control. Aortas isolated from 3–4 animals per group were taken. Two-way ANOVA test with Bonferroni post hoc test was used. Statistical significance for the highest concentration is given.

Among the genes that were highly amplified upon treatment with IFNγ and LPS was inducible nitric oxide synthase (*iNOS, Nos2*). Indeed, treatment of *WT-VSMCs* but not *STAT1^−/−^* with IFNγ and LPS caused amplified expression of *Nos2* as compared to stimulation with both factors alone (Fig. 5A). The RNA levels reflected nitrite accumulation in the medium (Fig. 5B). Since dysregulation of *Nos2* expression and its activity affects vessel function, we evaluated the physiological ramifications of these experimental conditions using a wire myograph/organ chamber setting. Stimulation of the aortic rings isolated from WT animals with IFNγ and LPS resulted in drastic impairment of contractility after subjection to norepinephrine treatment (Fig. 5C, left panel). WT vessels treated with both IFNγ and LPS manifested also high loss of the sensitivity to sodium nitroprusside (Fig. 5D, left panel). In contrast to WT, aortic rings from STAT1-deficient mice did not reveal ameliorated response to noradrenaline and sodium nitroprusside as compared to LPS stimulated vessel (Fig. 5C, Fig. 5D, right panel).

### STAT1 activation and CXCL9 and CXCL10 expression in ECs and VSMCs from human carotid atherosclerotic plaques

**Figure 6:**
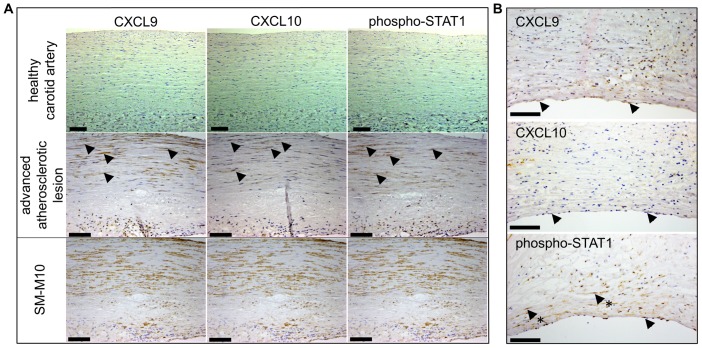
Expression of pSTAT1, CXCL9, CXCL10 in human atherosclerotic lesions *in situ*. Staining of the sections prepared from normal human artery exhibited no presence of pSTAT1, CXCL9, CXCL10 (A, upper panel). In contrast, all three proteins could be detected in SM-M10 positive cells in atherosclerotic plaques (A, middle panel) as well as in the endothelial cells at the lumen side (B). A representative analysis is shown of 6 human carotid atherosclerotic lesions and 4 healthy controls. Arrows represent examples of positive staining. In B arrows with asterix indicate examples of positively stained VSMCs. Scale bar = 100 µm.

We performed immunohistochemistry staining for phosphorylated STAT1, CXCL9 and CXCL10 in human advanced atherosclerotic plaques of carotid arteries in comparison to healthy vessels. As can clearly be observed in Figure 6A, VSMCs in the lesion highly expressed phosphorylated STAT1 and both chemokines CXCL9 and CXCL10. In contrast, healthy vessels were negative for all three markers (Figure 6A). Moreover, ECs covering the plaque likewise showed predominant staining for phosphorylated STAT1 and CXCL9, and to a lesser extent for CXCL10 (Figure 6B). Again, healthy endothelium was negative. Staining for IRF8 was more difficult to interpret, but seemed present at low levels in SMCs (not shown).

### STAT1-dependent pro-atherogenic target gene expression in human atherosclerotic plaques

**Figure 7:**
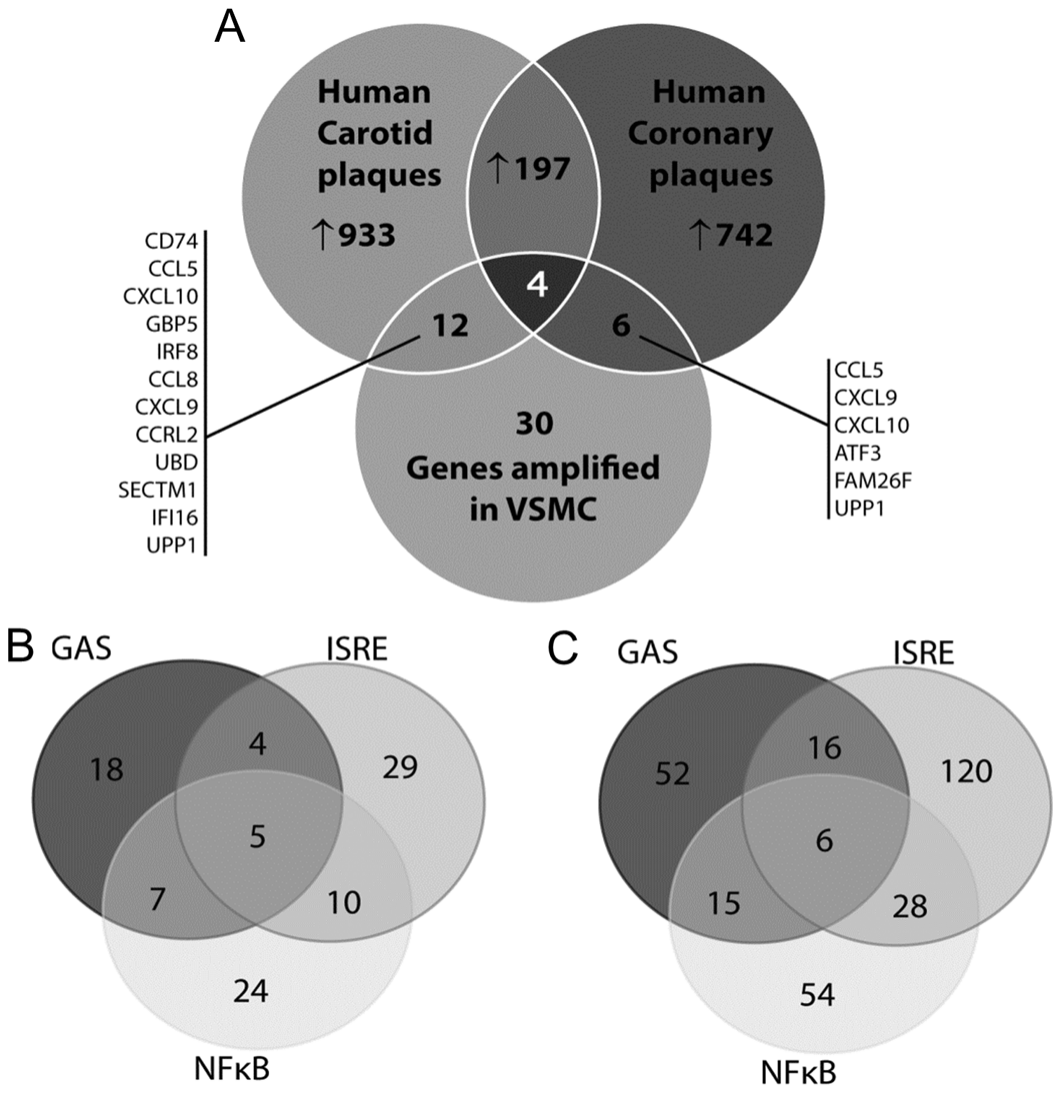
Expression of synergistically amplified genes in atherosclerotic vessels. A, Venn diagram with analysis of microarray datasets obtained from human coronary plaques and human carotid plaques. B, Promoter analysis of the differentially expressed genes in carotid (left panel) and coronary plaques (right panel). For details see text.

To obtain potential evidence for STAT1-mediated target gene expression in the human atherosclerotic plaque, we performed different types of experiments. First, we analyzed two independent microarray datasets obtained from human coronary plaques and human carotid plaques. These datasets are available in GEO NCBI database (acc. no. GSE40231 and GSE21545, respectively) [26], [27]. In coronary and carotid plaques respectively we identified 1146 and 949 genes upregulated at least 1.5 times as compared to the healthy arterial tissue (Figure 7A). 201 of those genes are commonly expressed between the different plaque tissues, highly implying that there are common features between coronary and carotid plaques (Sikorski et al. [36]). Detailed promoter analysis of the differentially expressed genes in carotid and coronary plaques identified 128 (Figure 7B) and 362 (Figure 7C) genes, respectively containing GAS, ISRE or NFκB sites, either alone or in different combinations. This strongly suggests also the cooperative involvement of NFκB, STAT1 and/or IRF in the transcriptional regulation of genes in the plaque tissue.

Next we compared the 30 IFNγ and LPS amplified STAT1-target genes listed in Table 1 to the genes upregulated in carotid and coronary plaques. Indeed, 12 out of the 30 genes were expressed in carotid plaques and 6 out of 30 in coronary plaques, including *CXCL9, CXCL10, CCL5, CCL8, CRCL2, Cd74, GBP5, UBD, SECTM1, IFI16* (homologue *Ifi-205*), *UPP1*, *FAM26F* and the transcription factor *IRF8* as the above identified STAT1 targets (Fig. 7A).

Together, this points to a pro-atherogenic role of STAT1 in vascular cells of atherosclerotic plaques, and suggests the potential of a selection of STAT1-target genes as biomarkers to monitor plaque phenotype in human atherosclerosis.

## Discussion

The involvement of STAT1 in experimental atherosclerosis has recently been appreciated, especially in immune cells. It is additionally accepted that in immune cells STAT1 is a unique point of convergence for the antimicrobial and inflammatory synergism between IFNγ and TLRs. Recently, we showed that also in ECs cross-talk between IFNγ and TLR4 resulted in augmented STAT1 phosphorylation and increased expression of the chemokine CXCL10 [17]. Here, a similar STAT1-dependent mechanism for CXCL10 expression in response to IFNγ and LPS was observed in VSMCs at the RNA and protein level (Fig. 1). To date, no information is available on the genome-wide induced changes modulated by IFNγ and TLR4 in ECs and VSMCs and how this affects vascular function. Therefore, we decided to further characterize the role of STAT1 in the transcriptional response pathways involved in the interaction between IFNγ and TLR4 signaling in VSMCs. Thus, we identified a specific set of STAT1-dependent genes that were synergistically affected by IFNγ and LPS in VSMCs *in vitro*. These included the chemokines *Cxcl9, Ccl12, Ccl8, Ccl5, Cxcl10* and *Ccrl2*, adhesion molecules (*Cd40, Cd74*), and the antiviral and antibacterial response genes *Irf8, Rsad2, Mx1, Oasl, Gbp5, Nos2, Batf2* and *Tnfrsf11a*. Based on their response pattern to IFNγ, LPS and IFNγ+LPS in *WT-VSMCs* (Fig. 2B), we could distinguish five clusters of genes (Fig. 2C). In general, the absence of STAT1 severely abrogated the IFNγ-induced expression of all of these genes. Moreover, the LPS response of 50% of genes listed in Table 1 was decreased in the absence of STAT1. Consequently, the synergistic effect of IFNγ and LPS, as seen in *WT-VSMCs*, could no longer be detected in *STAT1^−/−^-VSMCs*. This strongly suggests the involvement of STAT1 in the signal integration between JAK/STAT and TLR4 pathways. The IFNγ-induced expression of *Ccl8* appeared STAT1-independent, with a similar fold induction in WT and STAT1^−/−^ VSMCs. In contrast, absence of STAT1 increased its response to LPS. As *Ccl8* is a known STAT3 target gene [37], it is possible that it's IFNγ and LPS inducibility in WT and STAT1^−/−^ VSMCs, is regulated by this transcription factor. The increased LPS-mediated *Ccl8* expression in STAT1^−/−^ VSMCs as compared to WT cells, could potentially be explained by the absence of a STAT1-dependent inhibitory mechanism of STAT3 activity mediated by the STAT1-target gene SOCS1.

The expression of a selection of these genes, including *Ccl5*, *Cxcl9*, *Nos2*, *Irf8* and *Ccl12*, *Ccrl2* (not shown) was additionally determined at the RNA and protein level, and confirmed the microarray data. Moreover, relative quantification as fold change compared to control, resulted in a similar range of induction values for RNA and protein expression. A similar expression pattern of some of these genes could also be identified in ECs and aortic ring segments, providing evidence for a universal STAT1-dependent mechanism in vascular cells triggered by IFNγ and LPS.

Integration of IFNγ and TLR signaling pathways occurs, for instance, through synergy between TLR- and IFNγ-induced transcription factors. Promoter analysis of the genes listed in Table 1 indeed predicted the presence of STAT-NFκB and IRF-NFκB modules or combinations of separate ISRE, STAT or NFκB binding sites in their promoters. Indeed, ChIP-qPCR confirmed binding of STAT1, NFκB and IRF1 to the *Cxcl10* gene, in an IFNγ and LPS-dependent manner (Figure 3E). This strongly suggested that cooperation between NFκB, STAT1 and/or IRFs is involved in the transcriptional regulation of all of these genes in response to IFNγ and LPS. According to previous studies, transcription of genes that contain STAT1- and NFκB-binding sites in their promoter regions are often cooperatively regulated by extracellular stimuli that induce STAT1 and NFκB, such as IFNγ and TNFα, IL-1β or LPS [38], [39], [40], [41], [42], [43]. Likewise, genes with both an ISRE element and NFκB-binding site are subjected to a similar mechanism of signal integration [44], [45]. In general it is believed that in immune cells, multiple inflammatory stimuli culminate in gene expression that requires cooperation between NFκB and STAT1 or NFκB and IRF1 [7]. They ultimately promote type I immune actions, which are associated with host-defense mechanisms against viral and bacterial infections and excessive immune responses [46]. Our data provides strong evidence that a similar mechanism of signal integration exists in vascular cells. The difference in expression pattern of these 30 genes did not correlate with the presence of a specific binding site or combination of binding sites. This implies that the affinity of the different transcription factors is most likely determining the transcriptional response of a particular gene.

Among the amplified genes we also identified the transcription factor IRF8, which expression is thought to be restricted to lymphoid-cell lineages such as B-, T- and macrophages. Thus, IRF8 may in part account for “immune cell-specific” STAT1-dependent functions of IFNγ. IRF8 is also directly connected to TLR action, regulating the production of type I IFNs and other inflammatory mediators. For example, co-administration of IFNγ and LPS to macrophages caused super-induction of IRF8 and IRF8 target genes [47]. As a consequence, synergistic induction of the pro-inflammatory genes *IL1*, *IL6*, *IL12* and *TNFα* was observed in an IRF8 dependent manner. In addition to its known immune cell functions, our results now uncover a novel “inflammation-dependent” role of IRF8 in cells from the vasculature. In ECs as well as VSMCs, combined treatment of IFNγ and LPS resulted in a synergistic increase in IRF8 gene and protein expression as compared to both factors alone (Figure 4A and B). The presence of a potential STAT1-NFκB module in the IRF8 promoter (Table 1) highly suggests that the cooperation of these two transcription factors is at the basis of its synergistic expression. Consequently, this revealed the possible existence of IRF8-dependent cross-talk between IFNγ and LPS in vascular cells. Indeed, we subsequently identified *Ccl5* (but not *Cxcl9* and *Cxcl10*) as a novel IRF8 target in VSMCs and ECs (Fig. 4C and D). The transcriptional regulation of the *Ccl5* gene in macrophages in response to IFNγ and LPS, was recently shown to involve IRF8 in combination with IRF1 and NFκB [34]. Therefore, the IRF8-dependent expression of *Ccl5* in IFNγ-primed vascular cells that are subsequently stimulated by LPS is likely to comprise a similar mechanism. These results correlates with the predicted presence of an IRF-NFκB module in the *Ccl5* promoter (Table 1). On the other hand, the same promoter contains also a potential STAT1-NFκB module (Table 1) which suggests the additional involvement of STAT1 as well.

Together, our detailed investigation of STAT1-dependent transcriptional synergism between IFNγ and LPS in cells from the vasculature predicts the existence of different regulatory mechanisms. It particularly involves cooperation between STAT1, IRF1, IRF8 and NFκB, with the novel role of IRF8 providing an additional layer to the overall complexity.

Functional analysis of the STAT1-dependent genes that were synergistically affected by interactions between IFNγ and LPS in VSMCs (Table 1), revealed significant enrichment in biological functions connected to host defense, immune response, inflammatory response, cytokine response, response to stress and to wound healing (Table 2). All these categories generally represent a similar group of genes, which together reflect an enhanced pro-inflammatory and pro-atherogenic profile.

The fact that synergistic interactions between IFNγ and LPS in VSMCs resulted in increased expression of multiple chemokines, prompted us to investigate T-cell migration. Indeed, a significant increase in migration of CD3^+^/CD45^+^ splenocytes could be detected towards conditioned medium from IFNγ and LPS treated *WT-VSMCs* as compared to that from cells treated with single factors. Importantly, splenocytes migration occurred in a STAT1-dependent manner, which correlated with decreased chemokine expression in *STAT1^−/−^-VSMCs* under these conditions (Fig. 4E). Interestingly a subset of these chemokines, including CXCL9, CXCL10, CCL5, CCL8 and CCRL2, has been reported to be increased in cells from the vasculature, which is in agreement with our results. Moreover, evidence exists that chemokines cooperate in leukocyte recruitment to the injured artery during vascular remodeling [35], [48], [49] and as such are involved in the pathogenesis of atherosclerosis.

To further elucidate the functional role of a cross-talk in the vessel, we performed contractility studies. The signal integration between IFNγ and LPS in aortic ring segments resulted in impaired aortic contractility (Fig. 5) and coincided with a dramatic increase in expression of *Nos2*. Nos2 participates in vascular dysfunction and is associated with progression of atherosclerosis [50], [51].

More important, we were able to detect phosphorylated STAT1 in VSMCs and ECs of human atherosclerotic plaques (Fig. 6), which correlated with elevated expression of the chemokines *CXCL9* and *CXCL10*. Recently, Agrawal et al. [10] showed that STAT1 deficiency reduced foam cell formation in an intraperitoneal inflammation model and reduced atherosclerosis in an atherosclerosis-susceptible bone marrow transplantation mouse model. In combination with our results, this highlights the pro-atherogenic role of STAT1 in cells from the vasculature in human vascular disease.

Using data mining of human plaque transcriptomes, we were able to show that expression of a selection of the above identified STAT1-dependent pro-atherogenic genes was significantly increased in human plaques from carotid and coronary arteries (Fig. 7A). Of these, *CXCL9*, *CXCL10*, *CCL5*, *CCL8*, *CRCL2*, *Cd74* and *IRF8* have previously been implicated in atherosclerosis [52], [53]. This is not the case for *GBP5*, *Ubd*, *SectM1*, *Ifi16*, *Upp1* and *Fam26F*, and could therefore represent potential novel biomarkers of atherosclerosis. Moreover, *CCL5* expression was higher in carotid (23.3 fold increase) as compared to coronary (2.9 fold increase) plaques, which correlated with *IRF8* levels (8.8 fold increase in carotid vs. 1.4 fold increase in coronary). Detailed promoter analysis of differentially expressed genes in coronary and carotid plaques predicted cooperative involvement of NFκB, STAT1 and/or IRF1 in regulation of their expression. This could point to the role of IFNγ and TLR4 activation in human atherosclerosis, which is in agreement with previous studies [6], [54], [55]. However, we cannot rule out the contribution of other pro-inflammatory stimuli in the regulation of these genes. Nevertheless, our data strongly suggest involvement of both STAT1 and IRF8 in the regulation of gene expression in different cell types present in human atherosclerotic plaques.

In summary, our findings provide additional evidence to suggest that in ECs and VSMCs STAT1, in cooperation with IRF1, IRF8 and NFκB, coordinates a platform for cross-talk between IFNγ and TLR4. This results in an increased pro-inflammatory phenotype and leads to amplified pro-atherogenic responses in the vasculature. As a consequence, in the presence of IFNγ and LPS (or any other exogenous or endogenous TLR4 ligands), multiple chemokines, adhesion molecules and antiviral and antibacterial response proteins can be over-produced in ECs and VSMCs. This may in turn modulate leukocyte attraction, adhesion and VSMC proliferation and migration, which are important characteristics of vascular dysfunction and early triggers of atherosclerosis. As such, a predefined STAT1-target gene signature could be developed as a novel diagnostic tool to monitor and diagnose plaque phenotype in human atherosclerosis. In addition, STAT1 represents an interesting novel target of therapeutic intervention that has a crucial role in mediating the interplay between damaged vessels and host immunity during the process of atherosclerosis.

## Supporting Information

Table S1 **List of up and down-regulated genes in response to LPS in VSMCs** ***WT*** **and** ***STAT1^−/−^*** **.** Fold change compared to control.
(DOCX)

Table S2 **List of up and down-regulated genes in response to IFNγ in VSMCs** ***WT*** **and** ***STAT1^−/−^*** **.** Fold change compared to control.
(DOCX)

Table S3 **List of up and down-regulated genes in response to IFNγ and LPS in VSMCs** ***WT*** **and** ***STAT1^−/−^*** **.** Fold change compared to control.
(DOCX)

Table S4 **Primer sequences used in experimental procedures.**
(DOCX)

## References

[1] Orr AW, Hastings NE, Blackman BR, Wamhoff BR (2010) Complex regulation and function of the inflammatory smooth muscle cell phenotype in atherosclerosis. J Vasc Res 47:168–180. 19851078 10.1159/000250095 PMC2842170

[2] Hansson GK, Libby P (2006) The immune response in atherosclerosis: a double-edged sword. Nat Rev Immunol 6:508–519. 16778830 10.1038/nri1882

[3] Russell PS, Chase CM, Winn HJ, Colvin RB (1994) Coronary atherosclerosis in transplanted mouse hearts. III. Effects of recipient treatment with a monoclonal antibody to interferon-gamma. Transplantation 57:1367–1371. 7910422

[4] Gupta S, Pablo AM, Jiang X, Wang N, Tall AR, et al (1997) IFN-gamma potentiates atherosclerosis in ApoE knock-out mice. J Clin Invest 99:2752–2761. 9169506 10.1172/JCI119465 PMC508122

[5] Nagano H, Mitchell RN, Taylor MK, Hasegawa S, Tilney NL, et al (1997) Interferon-gamma deficiency prevents coronary arteriosclerosis but not myocardial rejection in transplanted mouse hearts. J Clin Invest 100:550–557. 9239401 10.1172/JCI119564 PMC508221

[6] Tellides G, Tereb DA, Kirkiles-Smith NC, Kim RW, Wilson JH, et al (2000) Interferon-gamma elicits arteriosclerosis in the absence of leukocytes. Nature 403:207–211. 10646607 10.1038/35003221

[7] Sikorski K, Chmielewski S, Olejnik A, Wesoly JZ, Heemann U, et al (2012) STAT1 as a central mediator of IFNgamma and TLR4 signal integration in vascular dysfunction. JAKSTAT 1:241–249. 24058779 10.4161/jkst.22469 PMC3670280

[8] Tamura T, Yanai H, Savitsky D, Taniguchi T (2008) The IRF family transcription factors in immunity and oncogenesis. Annu Rev Immunol 26:535–584. 18303999 10.1146/annurev.immunol.26.021607.090400

[9] Gough DJ, Levy DE, Johnstone RW, Clarke CJ (2008) IFNgamma signaling-does it mean JAK-STAT? Cytokine Growth Factor Rev 19:383–394. 18929502 10.1016/j.cytogfr.2008.08.004

[10] Agrawal S, Febbraio M, Podrez E, Cathcart MK, Stark GR, et al (2007) Signal transducer and activator of transcription 1 is required for optimal foam cell formation and atherosclerotic lesion development. Circulation 115:2939–2947. 17533179 10.1161/CIRCULATIONAHA.107.696922

[11] Bjorkbacka H (2006) Multiple roles of Toll-like receptor signaling in atherosclerosis. Curr Opin Lipidol 17:527–533. 16960501 10.1097/01.mol.0000245258.25387.ec

[12] Akira S, Uematsu S, Takeuchi O (2006) Pathogen recognition and innate immunity. Cell 124:783–801. 16497588 10.1016/j.cell.2006.02.015

[13] Schroder K, Sweet MJ, Hume DA (2006) Signal integration between IFNgamma and TLR signalling pathways in macrophages. Immunobiology 211:511–524. 16920490 10.1016/j.imbio.2006.05.007

[14] Hu X, Chen J, Wang L, Ivashkiv LB (2007) Crosstalk among Jak-STAT, Toll-like receptor, and ITAM-dependent pathways in macrophage activation. J Leukoc Biol 82:237–243. 17502339 10.1189/jlb.1206763

[15] Hu X, Chakravarty SD, Ivashkiv LB (2008) Regulation of interferon and Toll-like receptor signaling during macrophage activation by opposing feedforward and feedback inhibition mechanisms. Immunol Rev 226:41–56. 19161415 10.1111/j.1600-065X.2008.00707.x PMC2630590

[16] Hu X, Ivashkiv LB (2009) Cross-regulation of signaling pathways by interferon-gamma: implications for immune responses and autoimmune diseases. Immunity 31:539–550. 19833085 10.1016/j.immuni.2009.09.002 PMC2774226

[17] Sikorski K, Chmielewski S, Przybyl L, Heemann U, Wesoly J, et al (2011) STAT1-mediated signal integration between IFNgamma and LPS leads to increased EC and SMC activation and monocyte adhesion. Am J Physiol Cell Physiol 300:C1337–1344. 21346151 10.1152/ajpcell.00276.2010

[18] Holtschke T, Lohler J, Kanno Y, Fehr T, Giese N, et al (1996) Immunodeficiency and chronic myelogenous leukemia-like syndrome in mice with a targeted mutation of the ICSBP gene. Cell 87:307–317. 8861914 10.1016/s0092-8674(00)81348-3

[19] Geisterfer AA, Peach MJ, Owens GK (1988) Angiotensin II induces hypertrophy, not hyperplasia, of cultured rat aortic smooth muscle cells. Circ Res 62:749–756. 3280155 10.1161/01.res.62.4.749

[20] Ades EW, Candal FJ, Swerlick RA, George VG, Summers S, et al (1992) HMEC-1: establishment of an immortalized human microvascular endothelial cell line. J Invest Dermatol 99:683–690. 1361507 10.1111/1523-1747.ep12613748

[21] Livak KJ, Schmittgen TD (2001) Analysis of relative gene expression data using real-time quantitative PCR and the 2(−Delta Delta C(T)) Method. Methods 25:402–408. 11846609 10.1006/meth.2001.1262

[22] Chen H, Boutros PC (2011) VennDiagram: a package for the generation of highly-customizable Venn and Euler diagrams in R. BMC Bioinformatics 12:35. 21269502 10.1186/1471-2105-12-35 PMC3041657

[23] Cartharius K, Frech K, Grote K, Klocke B, Haltmeier M, et al (2005) MatInspector and beyond: promoter analysis based on transcription factor binding sites. Bioinformatics 21:2933–2942. 15860560 10.1093/bioinformatics/bti473

[24] Eden E, Navon R, Steinfeld I, Lipson D, Yakhini Z (2009) GOrilla: a tool for discovery and visualization of enriched GO terms in ranked gene lists. BMC Bioinformatics 10:48. 19192299 10.1186/1471-2105-10-48 PMC2644678

[25] Supek F, Bosnjak M, Skunca N, Smuc T (2011) REVIGO summarizes and visualizes long lists of gene ontology terms. PLoS One 6:e21800. 21789182 10.1371/journal.pone.0021800 PMC3138752

[26] Folkersen L, Persson J, Ekstrand J, Agardh HE, Hansson GK, et al (2012) Prediction of ischemic events on the basis of transcriptomic and genomic profiling in patients undergoing carotid endarterectomy. Mol Med 18:669–675. 22371308 10.2119/molmed.2011.00479 PMC3388132

[27] Hagg S, Skogsberg J, Lundstrom J, Noori P, Nilsson R, et al (2009) Multi-organ expression profiling uncovers a gene module in coronary artery disease involving transendothelial migration of leukocytes and LIM domain binding 2: the Stockholm Atherosclerosis Gene Expression (STAGE) study. PLoS Genet 5:e1000754. 19997623 10.1371/journal.pgen.1000754 PMC2780352

[28] Johnson WE, Li C, Rabinovic A (2007) Adjusting batch effects in microarray expression data using empirical Bayes methods. Biostatistics 8:118–127. 16632515 10.1093/biostatistics/kxj037

[29] Kallio MA, Tuimala JT, Hupponen T, Klemela P, Gentile M, et al (2011) Chipster: user-friendly analysis software for microarray and other high-throughput data. BMC Genomics 12:507. 21999641 10.1186/1471-2164-12-507 PMC3215701

[30] Barish GD, Yu RT, Karunasiri M, Ocampo CB, Dixon J, et al (2010) Bcl-6 and NF-kappaB cistromes mediate opposing regulation of the innate immune response. Genes Dev 24:2760–2765. 21106671 10.1101/gad.1998010 PMC3003193

[31] Stuehr DJ, Nathan CF (1989) Nitric oxide. A macrophage product responsible for cytostasis and respiratory inhibition in tumor target cells. J Exp Med 169:1543–1555. 2497225 10.1084/jem.169.5.1543 PMC2189318

[32] Guo F, Weih D, Meier E, Weih F (2007) Constitutive alternative NF-kappaB signaling promotes marginal zone B-cell development but disrupts the marginal sinus and induces HEV-like structures in the spleen. Blood 110:2381–2389. 17620454 10.1182/blood-2007-02-075143

[33] Mulvany MJ, Halpern W (1977) Contractile properties of small arterial resistance vessels in spontaneously hypertensive and normotensive rats. Circ Res 41:19–26. 862138 10.1161/01.res.41.1.19

[34] Liu J, Ma X (2006) Interferon regulatory factor 8 regulates RANTES gene transcription in cooperation with interferon regulatory factor-1, NF-kappaB, and PU.1. J Biol Chem 281:19188–19195. 16707500 10.1074/jbc.M602059200

[35] Zernecke A, Weber C (2010) Chemokines in the vascular inflammatory response of atherosclerosis. Cardiovasc Res 86:192–201. 20007309 10.1093/cvr/cvp391

[36] Sikorski K, Wesoly J, Bluyssen H (2014) Data Mining of Atherosclerotic Plaque Transcriptomes Predicts STAT1-Dependent Inflammatory Signal Integration in Vascular Disease. International Journal of Molecular Sciences 15:14313–14331. 25196434 10.3390/ijms150814313 PMC4159852

[37] Hiwatashi K, Tamiya T, Hasegawa E, Fukaya T, Hashimoto M, et al (2011) Suppression of SOCS3 in macrophages prevents cancer metastasis by modifying macrophage phase and MCP2/CCL8 induction. Cancer Lett 308:172–180. 21624767 10.1016/j.canlet.2011.04.024

[38] Lowenstein CJ, Alley EW, Raval P, Snowman AM, Snyder SH, et al (1993) Macrophage nitric oxide synthase gene: two upstream regions mediate induction by interferon gamma and lipopolysaccharide. Proc Natl Acad Sci U S A 90:9730–9734. 7692452 10.1073/pnas.90.20.9730 PMC47644

[39] Ohmori Y, Hamilton TA (2001) Requirement for STAT1 in LPS-induced gene expression in macrophages. J Leukoc Biol 69:598–604. 11310846

[40] Jahnke A, Johnson JP (1994) Synergistic activation of intercellular adhesion molecule 1 (ICAM-1) by TNF-alpha and IFN-gamma is mediated by p65/p50 and p65/c-Rel and interferon-responsive factor Stat1 alpha (p91) that can be activated by both IFN-gamma and IFN-alpha. FEBS Lett 354:220–226. 7957928 10.1016/0014-5793(94)01130-3

[41] Ohmori Y, Hamilton TA (1995) The interferon-stimulated response element and a kappa B site mediate synergistic induction of murine IP-10 gene transcription by IFN-gamma and TNF-alpha. J Immunol 154:5235–5244. 7730628

[42] Ohmori Y, Schreiber RD, Hamilton TA (1997) Synergy between interferon-gamma and tumor necrosis factor-alpha in transcriptional activation is mediated by cooperation between signal transducer and activator of transcription 1 and nuclear factor kappaB. J Biol Chem 272:14899–14907. 9169460 10.1074/jbc.272.23.14899

[43] Pine R (1997) Convergence of TNFalpha and IFNgamma signalling pathways through synergistic induction of IRF-1/ISGF-2 is mediated by a composite GAS/kappaB promoter element. Nucleic Acids Res 25:4346–4354. 9336467 10.1093/nar/25.21.4346 PMC147058

[44] Naschberger E, Werner T, Vicente AB, Guenzi E, Topolt K, et al (2004) Nuclear factor-kappaB motif and interferon-alpha-stimulated response element co-operate in the activation of guanylate-binding protein-1 expression by inflammatory cytokines in endothelial cells. Biochem J 379:409–420. 14741045 10.1042/BJ20031873 PMC1224089

[45] Voraberger G, Schafer R, Stratowa C (1991) Cloning of the human gene for intercellular adhesion molecule 1 and analysis of its 5′-regulatory region. Induction by cytokines and phorbol ester. J Immunol 147:2777–2786. 1680919

[46] O'Shea JJ, Ma A, Lipsky P (2002) Cytokines and autoimmunity. Nat Rev Immunol 2:37–45. 11905836 10.1038/nri702

[47] Zhao J, Kong HJ, Li H, Huang B, Yang M, et al (2006) IRF-8/interferon (IFN) consensus sequence-binding protein is involved in Toll-like receptor (TLR) signaling and contributes to the cross-talk between TLR and IFN-gamma signaling pathways. J Biol Chem 281:10073–10080. 16484229 10.1074/jbc.M507788200

[48] Braunersreuther V, Mach F, Steffens S (2007) The specific role of chemokines in atherosclerosis. Thromb Haemost 97:714–721. 17479181

[49] Seo D, Wang T, Dressman H, Herderick EE, Iversen ES, et al (2004) Gene expression phenotypes of atherosclerosis. Arterioscler Thromb Vasc Biol 24:1922–1927. 15297278 10.1161/01.ATV.0000141358.65242.1f

[50] Kuhlencordt PJ, Chen J, Han F, Astern J, Huang PL (2001) Genetic deficiency of inducible nitric oxide synthase reduces atherosclerosis and lowers plasma lipid peroxides in apolipoprotein E-knockout mice. Circulation 103:3099–3104. 11425775 10.1161/01.cir.103.25.3099

[51] Niu XL, Yang X, Hoshiai K, Tanaka K, Sawamura S, et al (2001) Inducible nitric oxide synthase deficiency does not affect the susceptibility of mice to atherosclerosis but increases collagen content in lesions. Circulation 103:1115–1120. 11222475 10.1161/01.cir.103.8.1115

[52] Martin-Ventura JL, Madrigal-Matute J, Munoz-Garcia B, Blanco-Colio LM, Van Oostrom M, et al (2009) Increased CD74 expression in human atherosclerotic plaques: contribution to inflammatory responses in vascular cells. Cardiovasc Res 83:586–594. 19423618 10.1093/cvr/cvp141

[53] Doring Y, Soehnlein O, Drechsler M, Shagdarsuren E, Chaudhari SM, et al (2012) Hematopoietic interferon regulatory factor 8-deficiency accelerates atherosclerosis in mice. Arterioscler Thromb Vasc Biol 32:1613–1623. 22556330 10.1161/ATVBAHA.111.236539

[54] Michelsen KS, Wong MH, Shah PK, Zhang W, Yano J, et al (2004) Lack of Toll-like receptor 4 or myeloid differentiation factor 88 reduces atherosclerosis and alters plaque phenotype in mice deficient in apolipoprotein E. Proc Natl Acad Sci U S A 101:10679–10684. 15249654 10.1073/pnas.0403249101 PMC489994

[55] Eid RE, Rao DA, Zhou J, Lo SF, Ranjbaran H, et al (2009) Interleukin-17 and interferon-gamma are produced concomitantly by human coronary artery-infiltrating T cells and act synergistically on vascular smooth muscle cells. Circulation 119:1424–1432. 19255340 10.1161/CIRCULATIONAHA.108.827618 PMC2898514

