# Supplementary material for: STAT1-Dependent Signal Integration between IFNγ and TLR4 in Vascular Cells Reflect Pro-Atherogenic Responses in Human Atherosclerosis
Source: PLoS One. 2014 Dec 5;9(12):e113318. doi: 10.1371/journal.pone.0113318 (PMC4257532; doi:10.1371/journal.pone.0113318)
Supplement: Table S1 — List of up and down-regulated genes in response to LPS in VSMCs WT and STAT1−/− . Fold change compared to control. (DOCX) [file pone.0113318.s001.docx]

### **Table S1.** List of up and down-regulated genes in response to LPS in VSMCs *WT* and *STAT1^-/^*^-^. Fold change compared to control.

| **ENTREZ ID** | **SYMBOL** | **Ratio**  **WT**  **LPS** | **p_value**  **WT**  **LPS** | **Ratio**  **STAT1^-/-^**  **LPS** | **p_value**  **STAT1^-/-^**  **LPS** |
| --- | --- | --- | --- | --- | --- |
| 15945 | Cxcl10 | 665.68 | 0.00 | 119.63 | 0.00 |
| 20304 | Ccl5 | 249.59 | 0.00 | 64.48 | 0.00 |
| 20210 | Saa3 | 213.37 | 0.00 | 87.07 | 0.00 |
| 58185 | Rsad2 | 209.45 | 0.00 | 2.93 | 0.50 |
| 12981 | Csf2 | 196.45 | 0.00 | 211.11 | 0.00 |
| 20296 | Ccl2 | 168.07 | 0.00 | 40.71 | 0.00 |
| 20306 | Ccl7 | 93.46 | 0.00 | 21.95 | 0.00 |
| 229898 | Gbp5 | 82.66 | 0.00 | 65.64 | 0.00 |
| 24047 | Ccl19 | 81.57 | 0.00 | 114.75 | 0.00 |
| 12642 | Ch25h | 60.31 | 0.00 | 25.57 | 0.01 |
| 80910 | Gpr84 | 54.75 | 0.00 | 34.16 | 0.01 |
| 21928 | Tnfaip2 | 45.55 | 0.00 | 28.83 | 0.01 |
| 16819 | Lcn2 | 43.24 | 0.00 | 25.09 | 0.02 |
| 14204 | Il4i1 | 42.82 | 0.00 | 18.47 | 0.00 |
| 54720 | Rcan1 | 42.65 | 0.00 | 15.71 | 0.05 |
| 22169 | Tyki | 40.77 | 0.00 | 1.56 | 0.41 |
| 626578 | Gbp10 | 40.15 | 0.01 | 20.93 | 0.03 |
| 12522 | Cd83 | 39.85 | 0.02 | 14.17 | 0.02 |
| 14825 | Cxcl1 | 38.59 | 0.01 | 42.36 | 0.00 |
| 18126 | Nos2 | 33.99 | 0.00 | 22.52 | 0.03 |
| 17858 | Mx2 | 31.85 | 0.00 | 1.44 | 0.46 |
| 227659 | Slc2a6 | 29.97 | 0.00 | 14.88 | 0.00 |
| 20568 | Slpi | 29.37 | 0.00 | 17.81 | 0.05 |
| 60533 | Cd274 | 26.42 | 0.00 | 6.07 | 0.01 |
| 547253 | Parp14 | 24.47 | 0.00 | 8.57 | 0.02 |
| 17392 | Mmp3 | 23.31 | 0.01 | 19.31 | 0.00 |
| 24088 | Tlr2 | 22.61 | 0.00 | 15.29 | 0.00 |
| 214854 | Lincr | 21.38 | 0.00 | 29.33 | 0.01 |
| 14469 | Gbp2 | 21.34 | 0.00 | 22.99 | 0.00 |
| 55932 | Gbp3 | 21.33 | 0.00 | 21.51 | 0.00 |
| 16193 | Il6 | 21.24 | 0.00 | 9.54 | 0.01 |
| 20344 | Selp | 21.11 | 0.00 | 21.15 | 0.00 |
| 17329 | Cxcl9 | 20.25 | 0.03 | 9.87 | 0.07 |
| 15894 | Icam1 | 20.25 | 0.00 | 8.48 | 0.01 |
| 230738 | Zc3h12a | 19.91 | 0.00 | 14.77 | 0.00 |
| 21929 | Tnfaip3 | 18.86 | 0.00 | 7.83 | 0.01 |
| 17750 | Mt2 | 16.47 | 0.00 | 11.60 | 0.01 |
| 229900 | Gbp6 | 15.84 | 0.01 | 15.28 | 0.05 |
| 223881 | Rnd1 | 15.23 | 0.01 | 18.42 | 0.00 |
| 21939 | Cd40 | 15.21 | 0.00 | 14.09 | 0.00 |
| 64292 | Ptges | 13.97 | 0.01 | 7.04 | 0.26 |
| 18037 | Nfkbie | 13.66 | 0.00 | 11.78 | 0.00 |
| 215900 | A630077B13Rik | 13.60 | 0.00 | 13.25 | 0.00 |
| 20715 | Serpina3g | 13.36 | 0.00 | 5.12 | 0.06 |
| 57875 | Angptl4 | 13.15 | 0.00 | 13.43 | 0.03 |
| 270893 | Tmem132e | 13.01 | 0.02 | 18.49 | 0.02 |
| 17857 | Mx1 | 12.73 | 0.01 | 1.68 | 0.67 |
| 66102 | Cxcl16 | 12.49 | 0.00 | 9.81 | 0.00 |
| 15891 | Ibsp | 12.38 | 0.01 | 3.01 | 0.56 |
| 80859 | Nfkbiz | 12.36 | 0.00 | 9.79 | 0.00 |
| 13123 | Cyp7b1 | 12.11 | 0.00 | 8.49 | 0.01 |
| 16803 | Lbp | 12.06 | 0.00 | 13.66 | 0.02 |
| 23962 | Oasl2 | 11.91 | 0.01 | 2.16 | 0.06 |
| 18035 | Nfkbia | 11.73 | 0.00 | 8.93 | 0.00 |
| 26410 | Map3k8 | 11.69 | 0.00 | 9.24 | 0.00 |
| 100048346 | LOC100048346 | 11.33 | 0.01 | 0.62 | 0.57 |
| 12061 | Bdkrb1 | 11.17 | 0.01 | 6.39 | 0.03 |
| 21822 | Tgtp | 11.09 | 0.00 | 9.78 | 0.00 |
| 19288 | Ptx3 | 10.73 | 0.01 | 17.38 | 0.03 |
| 18591 | Pdgfb | 10.70 | 0.00 | 4.64 | 0.02 |
| 14528 | Gch1 | 10.20 | 0.00 | 14.13 | 0.00 |
| 24110 | Usp18 | 9.90 | 0.01 | 0.85 | 0.97 |
| 76408 | Abcc3 | 9.68 | 0.00 | 7.80 | 0.01 |
| 19734 | Rgs16 | 9.54 | 0.00 | 10.55 | 0.03 |
| 667370 | LOC667370 | 9.51 | 0.00 | 0.50 | 0.06 |
| 20556 | Slfn2 | 9.30 | 0.01 | 3.12 | 0.01 |
| 12363 | Casp4 | 9.25 | 0.00 | 5.60 | 0.02 |
| 17386 | Mmp13 | 9.21 | 0.00 | 5.70 | 0.01 |
| 16145 | Igtp | 8.89 | 0.00 | 2.71 | 0.01 |
| 546546 | Serpina3h | 8.76 | 0.00 | 3.24 | 0.13 |
| 26570 | Slc7a11 | 8.74 | 0.00 | 6.35 | 0.00 |
| 15116 | Has1 | 8.71 | 0.01 | 11.64 | 0.04 |
| 22029 | Traf1 | 8.66 | 0.00 | 4.73 | 0.00 |
| 218454 | Lhfpl2 | 8.57 | 0.00 | 11.57 | 0.00 |
| 30794 | Pdlim4 | 8.35 | 0.02 | 3.80 | 0.04 |
| 22329 | Vcam1 | 8.32 | 0.01 | 9.75 | 0.00 |
| 12051 | Bcl3 | 8.21 | 0.00 | 6.14 | 0.03 |
| 54199 | Ccrl2 | 8.16 | 0.00 | 5.87 | 0.00 |
| 100038882 | LOC100038882 | 8.14 | 0.03 | 5.14 | 0.00 |
| 20295 | Ccl17 | 7.88 | 0.02 | 3.58 | 0.07 |
| 15439 | Hp | 7.75 | 0.04 | 5.92 | 0.05 |
| 231655 | Oasl1 | 7.61 | 0.00 | 1.45 | 0.71 |
| 71803 | Slc25a18 | 7.57 | 0.00 | 10.22 | 0.01 |
| 15377 | Foxa3 | 7.33 | 0.04 | 4.34 | 0.00 |
| 11541 | Adora2b | 7.23 | 0.00 | 4.46 | 0.07 |
| 574428 | Zmynd15 | 7.21 | 0.01 | 8.93 | 0.01 |
| 14962 | Cfb | 7.16 | 0.00 | 8.29 | 0.04 |
| 54123 | Irf7 | 7.00 | 0.02 | 0.84 | 0.75 |
| 16913 | Psmb8 | 7.00 | 0.01 | 4.63 | 0.02 |
| 16912 | Psmb9 | 6.97 | 0.01 | 3.46 | 0.00 |
| 433470 | AA467197 | 6.95 | 0.00 | 2.16 | 0.32 |
| 240354 | Malt1 | 6.78 | 0.00 | 4.63 | 0.01 |
| 20201 | S100a8 | 6.78 | 0.04 | 4.46 | 0.59 |
| 27056 | Irf5 | 6.69 | 0.00 | 4.07 | 0.00 |
| 435565 | LOC435565 | 6.69 | 0.02 | 1.41 | 0.58 |
| 102084 | AI451557 | 6.61 | 0.01 | 2.71 | 0.08 |
| 74190 | 1200009I06Rik | 6.54 | 0.01 | 4.60 | 0.01 |
| 226594 | Rcsd1 | 6.48 | 0.04 | 3.92 | 0.00 |
| 434484 | Sp140 | 6.46 | 0.00 | 7.29 | 0.05 |
| 16169 | Il15ra | 6.24 | 0.01 | 5.35 | 0.00 |
| 100047200 | LOC100047200 | 6.18 | 0.03 | 3.17 | 0.41 |
| 15959 | Ifit3 | 6.12 | 0.00 | 0.45 | 0.01 |
| 219132 | D14Ertd668e | 6.09 | 0.01 | 1.35 | 0.11 |
| 11639 | Ak3l1 | 5.97 | 0.00 | 5.23 | 0.04 |
| 67775 | Rtp4 | 5.95 | 0.02 | 0.98 | 0.96 |
| 19171 | Psmb10 | 5.93 | 0.00 | 5.33 | 0.00 |
| 54396 | Iigp2 | 5.92 | 0.01 | 2.26 | 0.04 |
| 20821 | Trim21 | 5.91 | 0.01 | 3.96 | 0.00 |
| 21934 | Tnfrsf11a | 5.87 | 0.04 | 7.44 | 0.05 |
| 15953 | Ifi47 | 5.87 | 0.01 | 5.55 | 0.00 |
| 11535 | Adm | 5.83 | 0.00 | 3.51 | 0.08 |
| 20312 | Cx3cl1 | 5.77 | 0.00 | 4.28 | 0.00 |
| 23961 | Oas1b | 5.76 | 0.00 | 0.77 | 0.41 |
| 240913 | Adamts4 | 5.75 | 0.01 | 3.62 | 0.00 |
| 19698 | Relb | 5.75 | 0.00 | 4.84 | 0.00 |
| 69146 | Gsdmdc1 | 5.71 | 0.01 | 3.77 | 0.04 |
| 16362 | Irf1 | 5.51 | 0.00 | 4.71 | 0.00 |
| 12013 | Bach1 | 5.37 | 0.00 | 5.06 | 0.00 |
| 12457 | Ccrn4l | 5.31 | 0.01 | 5.27 | 0.01 |
| 18034 | Nfkb2 | 5.26 | 0.00 | 3.63 | 0.00 |
| 56489 | Ikbke | 5.25 | 0.00 | 3.46 | 0.00 |
| 15937 | Ier3 | 5.10 | 0.00 | 3.73 | 0.00 |
| 15186 | Hdc | 4.99 | 0.00 | 3.25 | 0.01 |
| 243910 | Nfkbid | 4.98 | 0.00 | 5.61 | 0.02 |
| 80861 | Dhx58 | 4.91 | 0.00 | 1.71 | 0.02 |
| 20308 | Ccl9 | 4.86 | 0.02 | 5.11 | 0.02 |
| 12700 | Cish | 4.83 | 0.00 | 2.71 | 0.00 |
| 12122 | Bid | 4.82 | 0.00 | 3.67 | 0.00 |
| 630499 | EG630499 | 4.68 | 0.01 | 5.35 | 0.02 |
| 229003 | BC006779 | 4.67 | 0.00 | 1.86 | 0.01 |
| 100047934 | LOC100047934 | 4.66 | 0.00 | 5.08 | 0.00 |
| 21942 | Tnfrsf9 | 4.66 | 0.01 | 5.24 | 0.00 |
| 68709 | Cilp2 | 4.61 | 0.00 | 34.52 | 0.00 |
| 57783 | Tnip1 | 4.60 | 0.00 | 4.40 | 0.00 |
| 74165 | Fbxl22 | 4.58 | 0.00 | 4.17 | 0.00 |
| 20128 | Trim30 | 4.57 | 0.03 | 1.85 | 0.10 |
| 18033 | Nfkb1 | 4.56 | 0.00 | 3.99 | 0.00 |
| 213233 | Tapbpl | 4.51 | 0.00 | 5.26 | 0.00 |
| 239559 | A4galt | 4.51 | 0.04 | 3.91 | 0.00 |
| 14584 | Gfpt2 | 4.49 | 0.01 | 3.36 | 0.03 |
| 83671 | Sytl2 | 4.40 | 0.00 | 4.55 | 0.00 |
| 217154 | Stac2 | 4.39 | 0.04 | 5.90 | 0.20 |
| 106878 | 2010002N04Rik | 4.35 | 0.01 | 4.37 | 0.02 |
| 106766 | Stap2 | 4.33 | 0.04 | 3.23 | 0.05 |
| 11797 | Birc2 | 4.30 | 0.00 | 3.72 | 0.00 |
| 171209 | Accn3 | 4.29 | 0.01 | 5.92 | 0.00 |
| 12796 | Camp | 4.27 | 0.02 | 3.13 | 0.14 |
| 73914 | Irak3 | 4.25 | 0.00 | 2.65 | 0.08 |
| 54720 | Dscr1 | 4.17 | 0.00 | 1.99 | 0.12 |
| 22040 | Trex1 | 4.17 | 0.01 | 1.79 | 0.08 |
| 73167 | Arhgap8 | 4.16 | 0.03 | 2.44 | 0.13 |
| 30935 | Tor3a | 4.14 | 0.01 | 2.17 | 0.13 |
| 58244 | Stx6 | 4.13 | 0.00 | 3.33 | 0.00 |
| 17384 | Mmp10 | 4.08 | 0.01 | 3.21 | 0.12 |
| 231830 | Micall2 | 4.05 | 0.00 | 3.33 | 0.00 |
| 15976 | Ifnar2 | 3.98 | 0.00 | 3.98 | 0.00 |
| 74153 | Ube1l | 3.86 | 0.00 | 1.80 | 0.02 |
| 56437 | Rrad | 3.82 | 0.01 | 1.85 | 0.37 |
| 12266 | C3 | 3.77 | 0.00 | 3.40 | 0.02 |
| 100048554 | LOC100048554 | 3.75 | 0.00 | 44.53 | 0.00 |
| 22038 | Plscr1 | 3.73 | 0.00 | 2.71 | 0.01 |
| 100047963 | LOC100047963 | 3.72 | 0.01 | 1.70 | 0.09 |
| 15251 | Hif1a | 3.63 | 0.01 | 4.77 | 0.00 |
| 230073 | Ddx58 | 3.56 | 0.02 | 2.25 | 0.01 |
| 56791 | Ube2l6 | 3.53 | 0.00 | 2.59 | 0.01 |
| 107607 | Nod1 | 3.49 | 0.00 | 2.72 | 0.00 |
| 21950 | Tnfsf9 | 3.47 | 0.01 | 2.96 | 0.03 |
| 12505 | Cd44 | 3.45 | 0.00 | 4.39 | 0.00 |
| 381845 | 2310014L17Rik | 3.43 | 0.01 | 1.62 | 0.08 |
| 108116 | Slco3a1 | 3.42 | 0.00 | 2.67 | 0.02 |
| 24108 | Ubd | 3.38 | 0.00 | 4.71 | 0.00 |
| 207565 | Camkk2 | 3.36 | 0.00 | 2.97 | 0.02 |
| 77590 | 4631426J05Rik | 3.32 | 0.04 | 1.69 | 0.10 |
| 20847 | Stat2 | 3.31 | 0.01 | 1.41 | 0.06 |
| 20846 | Stat1 | 3.25 | 0.01 | 1.26 | 0.03 |
| 68252 | A030007L17Rik | 3.24 | 0.00 | 3.62 | 0.02 |
| 16164 | Il13ra1 | 3.24 | 0.01 | 2.45 | 0.26 |
| 20656 | Sod2 | 3.23 | 0.00 | 3.13 | 0.00 |
| 12475 | Cd14 | 3.21 | 0.00 | 3.60 | 0.04 |
| 12977 | Csf1 | 3.19 | 0.01 | 2.62 | 0.00 |
| 26464 | Vnn3 | 3.16 | 0.01 | 1.77 | 0.56 |
| 67338 | Rffl | 3.16 | 0.00 | 2.75 | 0.00 |
| 71898 | Apol9b | 3.14 | 0.03 | 0.78 | 0.70 |
| 52857 | Gramd1a | 3.12 | 0.00 | 4.46 | 0.01 |
| 18174 | Slc11a2 | 3.11 | 0.01 | 2.67 | 0.00 |
| 328949 | Mcc | 3.08 | 0.02 | 2.39 | 0.03 |
| 67800 | Dgat2 | 3.03 | 0.00 | 2.82 | 0.03 |
| 209086 | Samd9l | 3.00 | 0.03 | 1.81 | 0.01 |
| 226695 | Ifi205 | 2.99 | 0.01 | 4.24 | 0.18 |
| 74155 | Errfi1 | 2.98 | 0.00 | 2.60 | 0.05 |
| 100048858 | LOC100048858 | 2.96 | 0.00 | 1.41 | 0.20 |
| 100046255 | LOC100046255 | 2.94 | 0.03 | 3.31 | 0.24 |
| 227731 | Slc25a25 | 2.93 | 0.00 | 2.72 | 0.00 |
| 16149 | Cd74 | 2.93 | 0.01 | 8.01 | 0.00 |
| 52118 | Pvr | 2.93 | 0.01 | 2.69 | 0.01 |
| 12369 | Casp7 | 2.92 | 0.01 | 2.28 | 0.00 |
| 70757 | Ptplb | 2.91 | 0.01 | 2.51 | 0.01 |
| 12192 | Zfp36l1 | 2.89 | 0.01 | 2.05 | 0.03 |
| 243771 | Parp12 | 2.88 | 0.00 | 2.07 | 0.01 |
| 74646 | Spsb1 | 2.85 | 0.01 | 3.04 | 0.00 |
| 16918 | Mycl1 | 2.81 | 0.01 | 1.51 | 0.11 |
| 11828 | Aqp3 | 2.81 | 0.04 | 2.10 | 0.16 |
| 67916 | Ppap2b | 2.77 | 0.04 | 2.42 | 0.03 |
| 20893 | Bhlhb2 | 2.76 | 0.01 | 2.52 | 0.01 |
| 213053 | Slc39a14 | 2.74 | 0.00 | 2.33 | 0.01 |
| 209387 | AI451617 | 2.73 | 0.02 | 1.11 | 0.70 |
| 53608 | Map3k6 | 2.70 | 0.01 | 2.36 | 0.07 |
| 21355 | Tap2 | 2.69 | 0.01 | 2.41 | 0.01 |
| 12394 | Runx1 | 2.68 | 0.01 | 4.68 | 0.00 |
| 235442 | Rab8b | 2.68 | 0.01 | 2.24 | 0.06 |
| 18792 | Plau | 2.67 | 0.01 | 0.97 | 0.97 |
| 21354 | Tap1 | 2.66 | 0.01 | 1.43 | 0.04 |
| 432763 | Prr7 | 2.64 | 0.02 | 3.66 | 0.01 |
| 52552 | Parp8 | 2.63 | 0.00 | 2.12 | 0.02 |
| 170737 | Znrf1 | 2.63 | 0.00 | 2.34 | 0.00 |
| 21460 | Tcp10a | 2.62 | 0.00 | 3.54 | 0.01 |
| 14102 | Fas | 2.61 | 0.00 | 2.74 | 0.00 |
| 15018 | H2-Q7 | 2.61 | 0.02 | 2.20 | 0.09 |
| 18595 | Pdgfra | 2.59 | 0.00 | 2.14 | 0.27 |
| 96979 | Ptges2 | 2.57 | 0.01 | 2.53 | 0.00 |
| 11810 | Apobec1 | 2.55 | 0.01 | 1.67 | 0.26 |
| 70110 | Ifi35 | 2.54 | 0.02 | 2.22 | 0.00 |
| 71683 | Gypc | 2.54 | 0.01 | 2.00 | 0.04 |
| 229599 | Gm129 | 2.52 | 0.01 | 2.81 | 0.07 |
| 71733 | Susd2 | 2.52 | 0.00 | 1.99 | 0.04 |
| 21938 | Tnfrsf1b | 2.51 | 0.01 | 3.61 | 0.02 |
| 58194 | Sh3kbp1 | 2.51 | 0.00 | 2.08 | 0.46 |
| 75146 | Tmem180 | 2.50 | 0.04 | 1.82 | 0.05 |
| 26427 | Creb3l1 | 2.50 | 0.00 | 3.49 | 0.00 |
| 207259 | Zbtb7c | 2.50 | 0.03 | 2.23 | 0.07 |
| 52857 | D7Bwg0611e | 2.46 | 0.01 | 3.54 | 0.03 |
| 320508 | Cachd1 | 2.45 | 0.01 | 1.56 | 0.01 |
| 21807 | Tsc22d1 | 2.45 | 0.00 | 2.04 | 0.08 |
| 13163 | Daxx | 2.43 | 0.01 | 1.14 | 0.21 |
| 72747 | 2810439F02Rik | 2.42 | 0.00 | 1.29 | 0.48 |
| 208846 | Daam1 | 2.40 | 0.00 | 2.03 | 0.01 |
| 13197 | Gadd45a | 2.39 | 0.00 | 2.09 | 0.03 |
| 110532 | Adarb1 | 2.38 | 0.01 | 2.25 | 0.08 |
| 100045780 | LOC100045780 | 2.38 | 0.04 | 1.92 | 0.03 |
| 74748 | Slamf8 | 2.38 | 0.02 | 2.51 | 0.00 |
| 94221 | Gopc | 2.37 | 0.03 | 0.91 | 0.89 |
| 100046232 | LOC100046232 | 2.36 | 0.01 | 2.74 | 0.01 |
| 387609 | Zhx2 | 2.35 | 0.01 | 2.55 | 0.00 |
| 59028 | Rcl1 | 2.34 | 0.00 | 2.68 | 0.00 |
| 54721 | Tyk2 | 2.34 | 0.00 | 2.03 | 0.00 |
| 20442 | St3gal1 | 2.33 | 0.02 | 2.19 | 0.02 |
| 106869 | Tnfaip8 | 2.33 | 0.01 | 1.86 | 0.05 |
| 434204 | Whdc1 | 2.31 | 0.00 | 2.17 | 0.00 |
| 19246 | Ptpn1 | 2.31 | 0.00 | 2.54 | 0.02 |
| 209760 | Tmc7 | 2.31 | 0.01 | 1.78 | 0.02 |
| 17123 | Madcam1 | 2.30 | 0.04 | 2.57 | 0.01 |
| 19222 | Ptgir | 2.29 | 0.03 | 3.03 | 0.08 |
| 17765 | Mtf2 | 2.28 | 0.01 | 1.69 | 0.00 |
| 67712 | Slc25a37 | 2.28 | 0.04 | 1.93 | 0.01 |
| 23871 | Ets1 | 2.28 | 0.00 | 2.10 | 0.02 |
| 16477 | Junb | 2.27 | 0.01 | 2.33 | 0.01 |
| 17874 | Myd88 | 2.26 | 0.02 | 1.82 | 0.06 |
| 93692 | Glrx | 2.26 | 0.01 | 1.54 | 0.00 |
| 71712 | 1200002N14Rik | 2.25 | 0.03 | 2.65 | 0.00 |
| 14972 | H2-K1 | 2.24 | 0.05 | 2.12 | 0.07 |
| 21815 | Tgif1 | 2.22 | 0.01 | 1.90 | 0.00 |
| 18452 | P4ha2 | 2.22 | 0.00 | 2.46 | 0.00 |
| 21664 | Phlda1 | 2.22 | 0.00 | 1.85 | 0.01 |
| 67844 | Rab32 | 2.21 | 0.01 | 2.22 | 0.01 |
| 63872 | Zfp296 | 2.20 | 0.04 | 1.70 | 0.01 |
| 170439 | Elovl6 | 2.20 | 0.03 | 1.74 | 0.06 |
| 22030 | Traf2 | 2.20 | 0.00 | 1.68 | 0.01 |
| 17133 | Maff | 2.20 | 0.00 | 2.12 | 0.01 |
| 384309 | Trim56 | 2.19 | 0.03 | 1.88 | 0.07 |
| 67102 | D16Ertd472e | 2.18 | 0.00 | 1.98 | 0.00 |
| 240880 | Scyl3 | 2.18 | 0.01 | 2.67 | 0.02 |
| 16423 | Cd47 | 2.17 | 0.00 | 1.86 | 0.00 |
| 64143 | Ralb | 2.17 | 0.00 | 2.07 | 0.00 |
| 12263 | C2 | 2.17 | 0.05 | 1.03 | 0.94 |
| 75731 | 5133401N09Rik | 2.16 | 0.00 | 1.83 | 0.00 |
| 21814 | Tgfbr3 | 2.15 | 0.03 | 1.18 | 0.67 |
| 21356 | Tapbp | 2.14 | 0.03 | 1.96 | 0.00 |
| 244152 | Tsku | 2.14 | 0.00 | 2.12 | 0.01 |
| 233733 | Galntl4 | 2.12 | 0.04 | 2.16 | 0.03 |
| 71982 | Snx10 | 2.11 | 0.00 | 1.96 | 0.03 |
| 17190 | Mbd1 | 2.10 | 0.00 | 1.38 | 0.02 |
| 20850 | Stat5a | 2.10 | 0.01 | 2.16 | 0.00 |
| 14991 | H2-M3 | 2.10 | 0.03 | 1.52 | 0.30 |
| 13660 | Ehd1 | 2.09 | 0.01 | 2.00 | 0.01 |
| 27060 | Tcirg1 | 2.08 | 0.00 | 1.20 | 0.25 |
| 18573 | Pde1a | 2.07 | 0.00 | 1.84 | 0.56 |
| 72075 | Ogfr | 2.06 | 0.01 | 1.83 | 0.00 |
| 226442 | Zfp281 | 2.06 | 0.00 | 1.87 | 0.01 |
| 268396 | Sh3pxd2b | 2.06 | 0.02 | 1.71 | 0.00 |
| 81018 | Rnf114 | 2.03 | 0.02 | 1.60 | 0.06 |
| 21682 | Tec | 2.01 | 0.03 | 2.01 | 0.02 |
| 24059 | Slco2a1 | 2.01 | 0.04 | 1.30 | 0.30 |
| 214855 | Arid5a | 2.01 | 0.03 | 2.74 | 0.03 |
| 19271 | Ptprj | 2.00 | 0.02 | 1.84 | 0.11 |
| 192198 | Lrrc4 | 0.66 | 0.02 | 0.99 | 0.92 |
| 106565 | Dlk2 | 0.66 | 0.04 | 0.58 | 0.18 |
| 217030 | Ap1gbp1 | 0.66 | 0.00 | 0.70 | 0.02 |
| 227738 | Lrsam1 | 0.66 | 0.01 | 0.63 | 0.01 |
| 269831 | Tspan12 | 0.66 | 0.03 | 0.60 | 0.45 |
| 73095 | Slc25a42 | 0.66 | 0.02 | 0.72 | 0.30 |
| 226025 | Trpm3 | 0.66 | 0.04 | 0.83 | 0.44 |
| 73296 | Rhobtb3 | 0.66 | 0.00 | 0.78 | 0.08 |
| 242705 | E2f2 | 0.65 | 0.05 | 0.93 | 0.64 |
| 66522 | Pgpep1 | 0.65 | 0.03 | 0.66 | 0.09 |
| 76608 | Hectd3 | 0.65 | 0.00 | 0.60 | 0.01 |
| 232337 | Zfp637 | 0.65 | 0.00 | 0.78 | 0.03 |
| 171580 | Mical1 | 0.65 | 0.02 | 0.68 | 0.08 |
| 98432 | Phlpp | 0.65 | 0.01 | 0.72 | 0.17 |
| 212679 | Mars2 | 0.65 | 0.02 | 0.89 | 0.41 |
| 114663 | Impa2 | 0.65 | 0.03 | 0.39 | 0.01 |
| 108097 | Prkab2 | 0.65 | 0.05 | 0.68 | 0.07 |
| 245847 | Amdhd2 | 0.65 | 0.03 | 0.67 | 0.00 |
| 216459 | Myl6b | 0.65 | 0.03 | 0.71 | 0.06 |
| 29856 | Smtn | 0.64 | 0.00 | 0.75 | 0.30 |
| 282619 | Sbsn | 0.64 | 0.00 | 0.78 | 0.20 |
| 329470 | Accs | 0.64 | 0.01 | 0.53 | 0.13 |
| 30951 | Cbx8 | 0.64 | 0.01 | 0.68 | 0.05 |
| 670044 | LOC670044 | 0.64 | 0.01 | 0.68 | 0.22 |
| 13848 | Ephb6 | 0.64 | 0.00 | 0.59 | 0.33 |
| 18858 | Pmp22 | 0.64 | 0.03 | 0.57 | 0.04 |
| 106572 | Rab31 | 0.64 | 0.00 | 0.67 | 0.05 |
| 319517 | 6430510M02Rik | 0.64 | 0.03 | 0.67 | 0.34 |
| 74764 | Klc4 | 0.64 | 0.01 | 0.56 | 0.02 |
| 102098 | Arhgef18 | 0.64 | 0.00 | 0.66 | 0.06 |
| 20776 | Tmie | 0.64 | 0.02 | 0.56 | 0.06 |
| 64436 | Inpp5e | 0.64 | 0.00 | 0.63 | 0.06 |
| 107986 | Ddb2 | 0.64 | 0.04 | 0.63 | 0.16 |
| 19401 | Rara | 0.64 | 0.04 | 0.63 | 0.04 |
| 14107 | Fat1 | 0.64 | 0.03 | 1.02 | 0.88 |
| 22701 | Zfp41 | 0.63 | 0.05 | 0.72 | 0.03 |
| 26401 | Map3k1 | 0.63 | 0.02 | 0.48 | 0.02 |
| 57170 | Dolpp1 | 0.63 | 0.01 | 0.67 | 0.02 |
| 231600 | Chfr | 0.63 | 0.01 | 0.59 | 0.01 |
| 14924 | Magi1 | 0.63 | 0.04 | 0.80 | 0.26 |
| 67306 | Fam164a | 0.63 | 0.02 | 0.64 | 0.02 |
| 17388 | Mmp15 | 0.63 | 0.01 | 0.49 | 0.11 |
| 229011 | Samd10 | 0.63 | 0.02 | 0.66 | 0.05 |
| 408062 | BC062115 | 0.63 | 0.04 | 0.85 | 0.40 |
| 69226 | Snx24 | 0.63 | 0.05 | 0.70 | 0.09 |
| 97998 | Depdc6 | 0.63 | 0.03 | 0.89 | 0.77 |
| 230316 | Megf9 | 0.63 | 0.02 | 0.62 | 0.11 |
| 235406 | Snx33 | 0.63 | 0.01 | 0.59 | 0.00 |
| 67457 | Frmd8 | 0.63 | 0.00 | 0.92 | 0.76 |
| 381493 | S100a7a | 0.63 | 0.01 | 0.77 | 0.70 |
| 278097 | Armcx6 | 0.63 | 0.05 | 0.59 | 0.03 |
| 213056 | BC049806 | 0.63 | 0.01 | 0.75 | 0.14 |
| 13653 | Egr1 | 0.63 | 0.05 | 0.85 | 0.17 |
| 332397 | Nanos1 | 0.62 | 0.02 | 0.68 | 0.15 |
| 12036 | Bcat2 | 0.62 | 0.01 | 0.66 | 0.03 |
| 106639 | AI662250 | 0.62 | 0.01 | 0.59 | 0.00 |
| 72308 | Brf1 | 0.62 | 0.01 | 0.66 | 0.03 |
| 58188 | Vstm2b | 0.62 | 0.02 | 0.72 | 0.64 |
| 68910 | Zfp467 | 0.62 | 0.00 | 0.58 | 0.13 |
| 213027 | Evi5l | 0.62 | 0.00 | 0.61 | 0.00 |
| 20346 | Sema3a | 0.62 | 0.03 | 0.59 | 0.36 |
| 21422 | Tcfcp2 | 0.62 | 0.02 | 0.88 | 0.33 |
| 14367 | Fzd5 | 0.61 | 0.01 | 0.74 | 0.12 |
| 107747 | Aldh1l1 | 0.61 | 0.03 | 0.90 | 0.77 |
| 56364 | Zmym3 | 0.61 | 0.00 | 0.49 | 0.02 |
| 20410 | Sorbs3 | 0.61 | 0.01 | 0.57 | 0.01 |
| 20322 | Sord | 0.61 | 0.00 | 0.68 | 0.12 |
| 23828 | Bves | 0.61 | 0.01 | 1.03 | 0.95 |
| 78339 | Ttyh3 | 0.61 | 0.00 | 0.57 | 0.01 |
| 20443 | St3gal4 | 0.61 | 0.05 | 0.68 | 0.19 |
| 26404 | Map3k12 | 0.61 | 0.05 | 0.56 | 0.02 |
| 214424 | Parp16 | 0.61 | 0.03 | 0.67 | 0.02 |
| 18162 | Npr3 | 0.61 | 0.02 | 0.58 | 0.07 |
| 226518 | Nmnat2 | 0.61 | 0.01 | 0.99 | 0.90 |
| 14070 | F8a | 0.61 | 0.00 | 0.67 | 0.04 |
| 18541 | Pcnt | 0.61 | 0.01 | 0.50 | 0.01 |
| 67304 | 3110070M22Rik | 0.61 | 0.04 | 0.63 | 0.15 |
| 69544 | Wdr5b | 0.61 | 0.02 | 0.66 | 0.04 |
| 104252 | Cdc42ep2 | 0.60 | 0.02 | 0.68 | 0.05 |
| 24000 | Ptpn21 | 0.60 | 0.01 | 0.66 | 0.01 |
| 74032 | 4632417N05Rik | 0.60 | 0.00 | 0.68 | 0.04 |
| 243362 | Stard13 | 0.60 | 0.02 | 0.51 | 0.06 |
| 59095 | Fxyd6 | 0.60 | 0.05 | 0.72 | 0.59 |
| 20324 | Sdpr | 0.60 | 0.00 | 0.60 | 0.08 |
| 235493 | BC031353 | 0.60 | 0.00 | 0.54 | 0.01 |
| 70599 | Ssfa2 | 0.60 | 0.00 | 0.57 | 0.10 |
| 78779 | Spata2L | 0.60 | 0.03 | 0.57 | 0.00 |
| 208967 | Thnsl1 | 0.60 | 0.01 | 0.69 | 0.11 |
| 230815 | Man1c1 | 0.60 | 0.01 | 0.62 | 0.09 |
| 22379 | Fmnl3 | 0.60 | 0.01 | 0.66 | 0.07 |
| 231912 | Katnal1 | 0.60 | 0.00 | 0.92 | 0.91 |
| 338350 | 9330129D05Rik | 0.60 | 0.03 | 0.57 | 0.05 |
| 21833 | Thra | 0.60 | 0.01 | 0.61 | 0.00 |
| 71522 | Ggt6 | 0.59 | 0.02 | 1.01 | 0.98 |
| 71904 | Paqr7 | 0.59 | 0.02 | 0.49 | 0.03 |
| 217893 | Pacs2 | 0.59 | 0.00 | 0.66 | 0.06 |
| 230101 | Gba2 | 0.59 | 0.00 | 0.48 | 0.03 |
| 15213 | Hey1 | 0.59 | 0.03 | 0.61 | 0.06 |
| 14368 | Fzd6 | 0.59 | 0.02 | 0.70 | 0.09 |
| 94281 | Sfxn4 | 0.59 | 0.01 | 0.57 | 0.05 |
| 14709 | Gng8 | 0.59 | 0.04 | 0.56 | 0.03 |
| 210719 | Mkx | 0.59 | 0.00 | 0.46 | 0.14 |
| 69253 | Hspb2 | 0.59 | 0.00 | 0.62 | 0.11 |
| 233103 | 4931406P16Rik | 0.59 | 0.01 | 0.71 | 0.07 |
| 229285 | Spg20 | 0.58 | 0.00 | 0.73 | 0.06 |
| 11522 | Adh1 | 0.58 | 0.04 | 0.51 | 0.34 |
| 69551 | 2310022B05Rik | 0.58 | 0.00 | 0.56 | 0.07 |
| 192166 | Sardh | 0.58 | 0.00 | 1.07 | 0.73 |
| 212647 | Aldh4a1 | 0.58 | 0.00 | 0.55 | 0.01 |
| 13631 | Eef2k | 0.58 | 0.01 | 0.72 | 0.22 |
| 226744 | 9630058J23Rik | 0.58 | 0.01 | 0.58 | 0.00 |
| 232232 | Hdac11 | 0.58 | 0.01 | 0.51 | 0.01 |
| 12163 | Bmp8a | 0.58 | 0.04 | 0.59 | 0.06 |
| 93691 | Klf7 | 0.58 | 0.02 | 0.80 | 0.04 |
| 27355 | X99384 | 0.57 | 0.03 | 0.63 | 0.19 |
| 100044566 | LOC100044566 | 0.57 | 0.02 | 0.49 | 0.04 |
| 72507 | 2610524A10Rik | 0.57 | 0.02 | 0.57 | 0.05 |
| 71998 | Slc25a35 | 0.57 | 0.01 | 0.59 | 0.07 |
| 83429 | Ctns | 0.57 | 0.00 | 0.64 | 0.02 |
| 399566 | Btbd6 | 0.57 | 0.00 | 0.54 | 0.02 |
| 106369 | Ypel1 | 0.57 | 0.02 | 0.40 | 0.07 |
| 14794 | Spsb2 | 0.57 | 0.00 | 0.70 | 0.00 |
| 269424 | Phf17 | 0.57 | 0.03 | 0.48 | 0.03 |
| 13549 | Dyrk1b | 0.57 | 0.00 | 0.60 | 0.02 |
| 100044475 | LOC100044475 | 0.57 | 0.00 | 0.61 | 0.06 |
| 66949 | Trim59 | 0.57 | 0.00 | 0.67 | 0.02 |
| 231532 | Arhgap24 | 0.57 | 0.00 | 0.54 | 0.07 |
| 213019 | Pdlim2 | 0.56 | 0.02 | 0.54 | 0.02 |
| 74202 | Fblim1 | 0.56 | 0.02 | 0.70 | 0.34 |
| 240168 | Rasgrp3 | 0.56 | 0.02 | 0.52 | 0.06 |
| 21415 | Tcf3 | 0.56 | 0.00 | 0.54 | 0.01 |
| 71774 | 1300007L22Rik | 0.56 | 0.03 | 0.56 | 0.00 |
| 72852 | 2900024O10Rik | 0.56 | 0.01 | 0.65 | 0.04 |
| 272396 | Tarsl2 | 0.56 | 0.01 | 0.52 | 0.18 |
| 76976 | 2900062L11Rik | 0.56 | 0.04 | 0.53 | 0.38 |
| 72033 | Tsc22d2 | 0.56 | 0.00 | 0.73 | 0.10 |
| 230761 | BC039093 | 0.56 | 0.02 | 0.60 | 0.01 |
| 74137 | Nuak2 | 0.55 | 0.00 | 0.73 | 0.20 |
| 58178 | Sorcs1 | 0.55 | 0.01 | 1.07 | 0.53 |
| 16886 | Limk2 | 0.55 | 0.01 | 0.66 | 0.06 |
| 50490 | Nox4 | 0.55 | 0.01 | 0.59 | 0.17 |
| 22643 | Zfp101 | 0.55 | 0.01 | 0.56 | 0.02 |
| 329910 | Acot11 | 0.55 | 0.01 | 0.55 | 0.03 |
| 54371 | Chst2 | 0.55 | 0.01 | 0.54 | 0.04 |
| 217410 | Trib2 | 0.55 | 0.03 | 0.75 | 0.26 |
| 72749 | Nfkbil2 | 0.54 | 0.04 | 0.74 | 0.22 |
| 83767 | Wasf1 | 0.54 | 0.03 | 0.45 | 0.02 |
| 65962 | Slc9a3r2 | 0.54 | 0.01 | 0.54 | 0.04 |
| 78558 | Htra3 | 0.54 | 0.01 | 0.42 | 0.05 |
| 13639 | Efna4 | 0.54 | 0.03 | 0.67 | 0.30 |
| 268480 | Rapgefl1 | 0.54 | 0.01 | 0.55 | 0.03 |
| 234857 | Spire2 | 0.54 | 0.05 | 0.54 | 0.04 |
| 19339 | Rab3a | 0.53 | 0.00 | 0.54 | 0.01 |
| 210710 | Gab3 | 0.53 | 0.01 | 0.57 | 0.03 |
| 17309 | Mgat3 | 0.53 | 0.01 | 0.61 | 0.03 |
| 140742 | Sesn1 | 0.53 | 0.00 | 0.48 | 0.01 |
| 12411 | Cbs | 0.53 | 0.00 | 0.64 | 0.40 |
| 215114 | Hip1 | 0.53 | 0.04 | 0.65 | 0.05 |
| 81004 | Tbl1xr1 | 0.53 | 0.02 | 0.73 | 0.38 |
| 213573 | Efcab4a | 0.53 | 0.02 | 0.53 | 0.10 |
| 19725 | Rfx2 | 0.53 | 0.02 | 0.53 | 0.07 |
| 54635 | Pdgfc | 0.53 | 0.03 | 0.51 | 0.02 |
| 74769 | Pik3cb | 0.52 | 0.02 | 0.62 | 0.04 |
| 14302 | Frk | 0.52 | 0.01 | 0.76 | 0.10 |
| 235431 | Coro2b | 0.52 | 0.02 | 0.63 | 0.36 |
| 16007 | Cyr61 | 0.52 | 0.00 | 0.68 | 0.13 |
| 19200 | Pstpip1 | 0.52 | 0.01 | 0.46 | 0.04 |
| 235050 | Zfp810 | 0.52 | 0.03 | 0.54 | 0.03 |
| 216505 | Pik3ip1 | 0.52 | 0.02 | 0.56 | 0.09 |
| 211652 | Wwc1 | 0.51 | 0.00 | 0.57 | 0.04 |
| 73680 | 2410081M15Rik | 0.51 | 0.01 | 0.60 | 0.09 |
| 100213 | Rusc2 | 0.51 | 0.00 | 0.54 | 0.08 |
| 53310 | Dlg3 | 0.51 | 0.00 | 0.52 | 0.00 |
| 245666 | Iqsec2 | 0.51 | 0.01 | 0.39 | 0.02 |
| 100044736 | LOC100044736 | 0.51 | 0.02 | 0.60 | 0.11 |
| 98496 | 5033414K04Rik | 0.51 | 0.02 | 0.46 | 0.03 |
| 70122 | Mllt3 | 0.50 | 0.00 | 0.36 | 0.04 |
| 71059 | Hexim2 | 0.50 | 0.01 | 0.63 | 0.15 |
| 76454 | Fbxo31 | 0.50 | 0.00 | 0.64 | 0.04 |
| 21899 | Tlr6 | 0.50 | 0.04 | 0.60 | 0.07 |
| 53412 | Ppp1r3c | 0.50 | 0.04 | 0.43 | 0.01 |
| 11302 | Aatk | 0.50 | 0.01 | 0.41 | 0.18 |
| 244238 | Mrgpre | 0.50 | 0.03 | 0.48 | 0.00 |
| 89867 | Sec16b | 0.50 | 0.00 | 0.52 | 0.09 |
| 237353 | Sh3md4 | 0.50 | 0.01 | 0.55 | 0.06 |
| 11555 | Adrb2 | 0.49 | 0.01 | 0.59 | 0.09 |
| 19400 | Rapsn | 0.49 | 0.03 | 0.52 | 0.35 |
| 14388 | Gab1 | 0.49 | 0.00 | 0.51 | 0.09 |
| 72309 | Tmem158 | 0.48 | 0.02 | 0.37 | 0.04 |
| 12159 | Bmp4 | 0.48 | 0.00 | 0.55 | 0.11 |
| 15394 | Hoxa1 | 0.48 | 0.00 | 0.58 | 0.03 |
| 27528 | D0H4S114 | 0.48 | 0.01 | 0.39 | 0.00 |
| 209039 | Tenc1 | 0.48 | 0.01 | 0.54 | 0.00 |
| 434197 | Fam169b | 0.48 | 0.02 | 0.43 | 0.00 |
| 15478 | Hs3st3a1 | 0.47 | 0.02 | 0.37 | 0.00 |
| 17268 | Meis1 | 0.47 | 0.00 | 0.52 | 0.06 |
| 67198 | 2810022L02Rik | 0.47 | 0.02 | 0.44 | 0.18 |
| 233315 | BB128963 | 0.47 | 0.01 | 0.49 | 0.03 |
| 73061 | 3110007F17Rik | 0.47 | 0.01 | 0.48 | 0.00 |
| 54366 | Ctnnal1 | 0.46 | 0.00 | 0.47 | 0.01 |
| 546161 | C85627 | 0.46 | 0.01 | 0.46 | 0.20 |
| 17260 | Mef2c | 0.46 | 0.04 | 0.38 | 0.14 |
| 103511 | BB146404 | 0.46 | 0.00 | 0.57 | 0.26 |
| 23882 | Gadd45g | 0.45 | 0.01 | 0.58 | 0.05 |
| 20947 | Swap70 | 0.45 | 0.01 | 0.48 | 0.03 |
| 93689 | Lmod1 | 0.45 | 0.01 | 0.59 | 0.21 |
| 64929 | Scel | 0.44 | 0.02 | 0.42 | 0.17 |
| 272636 | D9Ertd280e | 0.44 | 0.04 | 0.52 | 0.24 |
| 71721 | Fam13c | 0.44 | 0.00 | 0.53 | 0.07 |
| 211401 | Mtss1 | 0.44 | 0.03 | 0.46 | 0.01 |
| 57265 | Fzd2 | 0.44 | 0.03 | 0.43 | 0.05 |
| 66985 | Rassf7 | 0.43 | 0.00 | 0.57 | 0.01 |
| 15401 | Hoxa4 | 0.43 | 0.01 | 0.44 | 0.02 |
| 236899 | Pcyt1b | 0.43 | 0.02 | 0.43 | 0.09 |
| 192734 | AI646023 | 0.43 | 0.01 | 0.46 | 0.14 |
| 69274 | Ctdspl | 0.43 | 0.00 | 0.49 | 0.01 |
| 27428 | Shroom3 | 0.43 | 0.00 | 0.53 | 0.07 |
| 215690 | Nav1 | 0.43 | 0.02 | 0.51 | 0.14 |
| 21917 | Tmpo | 0.42 | 0.01 | 0.57 | 0.04 |
| 26930 | Ppnr | 0.42 | 0.05 | 0.53 | 0.16 |
| 242297 | Fam110b | 0.42 | 0.00 | 0.48 | 0.00 |
| 114142 | Foxp2 | 0.42 | 0.00 | 0.39 | 0.07 |
| 23937 | Mab21l2 | 0.42 | 0.00 | 0.51 | 0.45 |
| 18003 | Nedd9 | 0.42 | 0.00 | 0.57 | 0.04 |
| 100342 | 4732473B16Rik | 0.42 | 0.01 | 0.66 | 0.06 |
| 381175 | Ccdc68 | 0.41 | 0.03 | 0.47 | 0.36 |
| 58226 | Cacna1h | 0.41 | 0.00 | 0.38 | 0.25 |
| 102371 | AA407270 | 0.40 | 0.02 | 0.41 | 0.11 |
| 226352 | Epb4.1l5 | 0.40 | 0.00 | 0.47 | 0.02 |
| 68490 | Zfp579 | 0.40 | 0.01 | 0.46 | 0.11 |
| 15460 | Hr | 0.39 | 0.02 | 0.53 | 0.21 |
| 66895 | 1300014I06Rik | 0.39 | 0.00 | 0.48 | 0.11 |
| 225341 | Lims2 | 0.39 | 0.00 | 0.50 | 0.14 |
| 68283 | 9530077C05Rik | 0.39 | 0.00 | 0.43 | 0.01 |
| 269023 | Zfp608 | 0.38 | 0.01 | 0.45 | 0.02 |
| 72293 | Nkd2 | 0.38 | 0.04 | 0.34 | 0.05 |
| 13170 | Dbp | 0.38 | 0.00 | 0.35 | 0.01 |
| 20682 | Sox9 | 0.37 | 0.01 | 0.51 | 0.05 |
| 23859 | Dlg2 | 0.37 | 0.03 | 0.55 | 0.57 |
| 21679 | Tead4 | 0.35 | 0.00 | 0.45 | 0.01 |
| 16511 | Kcnh2 | 0.35 | 0.00 | 0.36 | 0.09 |
| 70788 | Klhl30 | 0.35 | 0.00 | 0.41 | 0.15 |
| 215748 | Cnksr3 | 0.34 | 0.00 | 0.46 | 0.03 |
| 15360 | Hmgcs2 | 0.34 | 0.05 | 0.32 | 0.37 |
| 55984 | Camkk1 | 0.33 | 0.00 | 0.34 | 0.02 |
| 71706 | Slc46a3 | 0.33 | 0.00 | 0.33 | 0.01 |
| 14200 | Fhl2 | 0.33 | 0.00 | 0.38 | 0.01 |
| 244867 | Arhgap20 | 0.33 | 0.03 | 0.57 | 0.13 |
| 77889 | Lbh | 0.33 | 0.03 | 0.44 | 0.00 |
| 68918 | 1190005I06Rik | 0.33 | 0.02 | 0.32 | 0.08 |
| 114301 | Palmd | 0.28 | 0.01 | 0.40 | 0.04 |
| 320365 | 9330186A19Rik | 0.28 | 0.02 | 0.28 | 0.01 |
| 67839 | Gpsm1 | 0.28 | 0.00 | 0.38 | 0.01 |
| 192786 | Rapgef6 | 0.26 | 0.00 | 0.22 | 0.03 |
| 14373 | G0s2 | 0.23 | 0.04 | 0.27 | 0.25 |
| 30937 | Lmcd1 | 0.23 | 0.00 | 0.26 | 0.00 |
| 14370 | Fzd8 | 0.20 | 0.01 | 0.28 | 0.00 |
| 208164 | BC064033 | 0.19 | 0.01 | 0.27 | 0.01 |
| 15214 | Hey2 | 0.17 | 0.01 | 0.34 | 0.21 |
