# Supplementary material for: STAT1-Dependent Signal Integration between IFNγ and TLR4 in Vascular Cells Reflect Pro-Atherogenic Responses in Human Atherosclerosis
Source: PLoS One. 2014 Dec 5;9(12):e113318. doi: 10.1371/journal.pone.0113318 (PMC4257532; doi:10.1371/journal.pone.0113318)
Supplement: Table S2 — List of up and down-regulated genes in response to IFNγ in VSMCs WT and STAT1−/− . Fold change compared to control. (DOCX) [file pone.0113318.s002.docx]

### Table S2. List of up and down-regulated genes in response to IFNγ in VSMCs *WT* and *STAT1^-/-^*^.^ Fold change compared to control.

| **ENTREZ ID** | **SYMBOL** | **Ratio**  **WT**  **IFNγ** | **p_value**  **WT**  **IFNγ** | **Ratio**  **STAT1^-/-^**  **IFNγ** | **p_value**  **STAT1^-/-^**  **IFNγ** |
| --- | --- | --- | --- | --- | --- |
| 15945 | Cxcl10 | 343.62 | 0.00 | 0.47 | 0.28 |
| 21822 | Tgtp | 182.91 | 0.00 | 2.93 | 0.02 |
| 12265 | Ciita | 153.84 | 0.00 | 1.07 | 0.84 |
| 17329 | Cxcl9 | 150.73 | 0.00 | 0.93 | 0.70 |
| 225594 | LOC225594 | 136.83 | 0.00 | 0.41 | 0.22 |
| 74481 | Batf2 | 134.37 | 0.00 | 5.35 | 0.16 |
| 16145 | Igtp | 104.95 | 0.00 | 1.16 | 0.41 |
| 435565 | LOC435565 | 92.64 | 0.00 | 0.87 | 0.82 |
| 626578 | Gbp10 | 81.44 | 0.00 | 0.99 | 0.97 |
| 60533 | Cd274 | 78.01 | 0.00 | 0.97 | 0.90 |
| 102084 | AI451557 | 77.90 | 0.00 | 1.03 | 0.76 |
| 55932 | Gbp3 | 72.35 | 0.00 | 0.97 | 0.98 |
| 14961 | H2-Ab1 | 57.18 | 0.00 | 0.74 | 0.44 |
| 16912 | Psmb9 | 56.81 | 0.00 | 0.85 | 0.34 |
| 229898 | Gbp5 | 53.48 | 0.00 | 1.28 | 0.75 |
| 229900 | Gbp6 | 50.68 | 0.00 | 1.65 | 0.35 |
| 433470 | AA467197 | 47.10 | 0.00 | 1.68 | 0.71 |
| 23962 | Oasl2 | 45.08 | 0.00 | 0.71 | 0.38 |
| 215900 | A630077B13Rik | 43.02 | 0.00 | 0.95 | 0.85 |
| 14469 | Gbp2 | 40.01 | 0.00 | 1.01 | 0.92 |
| 547253 | Parp14 | 38.97 | 0.00 | 0.99 | 0.97 |
| 16149 | Cd74 | 37.66 | 0.00 | 0.78 | 0.27 |
| 15953 | Ifi47 | 37.21 | 0.00 | 1.10 | 0.52 |
| 16913 | Psmb8 | 37.04 | 0.00 | 0.96 | 0.93 |
| 641240 | LOC641240 | 33.51 | 0.00 | 1.07 | 0.64 |
| 22169 | Tyki | 25.09 | 0.00 | 1.33 | 0.30 |
| 20715 | Serpina3g | 24.76 | 0.00 | 2.12 | 0.33 |
| 74748 | Slamf8 | 24.47 | 0.00 | 1.02 | 0.95 |
| 16362 | Irf1 | 23.54 | 0.00 | 1.21 | 0.03 |
| 54396 | Iigp2 | 23.39 | 0.00 | 1.26 | 0.50 |
| 58185 | Rsad2 | 22.91 | 0.00 | 1.53 | 0.58 |
| 24108 | Ubd | 21.54 | 0.00 | 0.80 | 0.12 |
| 14969 | H2-Eb1 | 20.66 | 0.00 | 1.07 | 0.59 |
| 667370 | LOC667370 | 20.43 | 0.00 | 0.61 | 0.01 |
| 20306 | Ccl7 | 20.03 | 0.00 | 1.05 | 0.85 |
| 20846 | Stat1 | 19.26 | 0.00 | 1.13 | 0.37 |
| 54123 | Irf7 | 15.67 | 0.00 | 0.70 | 0.87 |
| 21354 | Tap1 | 15.09 | 0.00 | 0.88 | 0.11 |
| 15186 | Hdc | 14.78 | 0.00 | 1.11 | 0.75 |
| 219132 | D14Ertd668e | 14.73 | 0.00 | 1.06 | 0.82 |
| 19171 | Psmb10 | 13.98 | 0.00 | 1.16 | 0.37 |
| 56791 | Ube2l6 | 13.18 | 0.00 | 1.02 | 0.90 |
| 74153 | Ube1l | 13.12 | 0.00 | 1.01 | 0.97 |
| 23960 | Oas1g | 13.07 | 0.00 | 0.68 | 0.79 |
| 12904 | Crabp2 | 13.03 | 0.00 | 1.13 | 0.65 |
| 100038882 | LOC100038882 | 12.16 | 0.00 | 1.16 | 0.35 |
| 20128 | Trim30 | 11.90 | 0.01 | 0.97 | 0.85 |
| 56045 | Samhd1 | 11.54 | 0.00 | 1.05 | 0.68 |
| 434484 | Sp140 | 11.24 | 0.00 | 1.76 | 0.44 |
| 100048346 | LOC100048346 | 11.04 | 0.01 | 0.72 | 0.56 |
| 620913 | OTTMUSG00000005523 | 10.97 | 0.00 | 0.97 | 0.98 |
| 213233 | Tapbpl | 10.80 | 0.00 | 1.09 | 0.50 |
| 15959 | Ifit3 | 10.42 | 0.00 | 0.60 | 0.00 |
| 20821 | Trim21 | 9.87 | 0.00 | 1.04 | 0.32 |
| 71898 | Apol9b | 9.67 | 0.00 | 0.48 | 0.55 |
| 12263 | C2 | 9.44 | 0.00 | 1.03 | 0.97 |
| 24110 | Usp18 | 9.43 | 0.01 | 1.12 | 0.87 |
| 20296 | Ccl2 | 8.62 | 0.00 | 0.97 | 0.89 |
| 67775 | Rtp4 | 8.48 | 0.00 | 0.50 | 0.23 |
| 23969 | Pacsin1 | 8.42 | 0.00 | 0.94 | 0.91 |
| 243771 | Parp12 | 8.35 | 0.00 | 0.78 | 0.04 |
| 17858 | Mx2 | 8.10 | 0.00 | 2.31 | 0.10 |
| 15930 | Indo | 7.80 | 0.00 | 0.79 | 0.38 |
| 14960 | H2-Aa | 7.76 | 0.03 | 0.20 | 0.00 |
| 60440 | AW111922 | 7.59 | 0.00 | 0.69 | 0.15 |
| 21355 | Tap2 | 7.31 | 0.00 | 0.93 | 0.70 |
| 20847 | Stat2 | 7.06 | 0.00 | 1.02 | 0.87 |
| 80861 | Dhx58 | 7.02 | 0.00 | 1.03 | 0.98 |
| 14998 | H2-DMa | 6.59 | 0.00 | 0.90 | 0.88 |
| 21928 | Tnfaip2 | 6.52 | 0.00 | 1.14 | 0.89 |
| 16169 | Il15ra | 6.49 | 0.00 | 0.95 | 0.72 |
| 23961 | Oas1b | 6.39 | 0.00 | 0.79 | 0.69 |
| 219103 | Cenpj | 6.38 | 0.00 | 0.35 | 0.17 |
| 22375 | Wars | 6.22 | 0.00 | 1.21 | 0.09 |
| 12363 | Casp4 | 6.15 | 0.00 | 1.57 | 0.52 |
| 15900 | Irf8 | 6.13 | 0.00 | 1.40 | 0.29 |
| 100048554 | LOC100048554 | 6.12 | 0.01 | 5.21 | 0.33 |
| 16174 | Il18rap | 6.04 | 0.02 | 1.12 | 1.00 |
| 74568 | Mlkl | 5.91 | 0.00 | 1.12 | 0.80 |
| 22436 | Xdh | 5.81 | 0.00 | 1.64 | 0.54 |
| 12258 | Serping1 | 5.80 | 0.00 | 1.07 | 0.94 |
| 15018 | H2-Q7 | 5.59 | 0.00 | 0.77 | 0.14 |
| 12362 | Casp1 | 5.56 | 0.02 | 1.11 | 0.93 |
| 69550 | Bst2 | 5.54 | 0.00 | 0.75 | 0.35 |
| 226695 | Ifi205 | 5.39 | 0.00 | 0.64 | 0.92 |
| 59027 | Nampt | 5.23 | 0.00 | 0.73 | 0.77 |
| 70110 | Ifi35 | 5.22 | 0.00 | 1.18 | 0.39 |
| 328561 | 9130218O11Rik | 5.21 | 0.00 | 1.04 | 0.87 |
| 12703 | Socs1 | 5.19 | 0.00 | 1.00 | 1.00 |
| 15040 | H2-T23 | 5.04 | 0.00 | 1.01 | 0.95 |
| 17067 | Ly6c1 | 4.95 | 0.00 | 1.39 | 0.22 |
| 12006 | Axin2 | 4.91 | 0.00 | 1.06 | 0.79 |
| 12916 | Crem | 4.85 | 0.00 | 1.11 | 0.52 |
| 66892 | Eif4e3 | 4.83 | 0.00 | 1.29 | 0.24 |
| 14999 | H2-DMb1 | 4.78 | 0.00 | 0.97 | 0.94 |
| 69146 | Gsdmdc1 | 4.72 | 0.00 | 0.96 | 0.95 |
| 15894 | Icam1 | 4.71 | 0.00 | 1.11 | 0.84 |
| 230073 | Ddx58 | 4.64 | 0.00 | 0.90 | 0.39 |
| 209387 | AI451617 | 4.35 | 0.00 | 1.09 | 0.64 |
| 217203 | Tmem106a | 4.19 | 0.00 | 0.61 | 0.12 |
| 54199 | Ccrl2 | 4.18 | 0.00 | 0.76 | 0.10 |
| 17069 | Ly6e | 4.17 | 0.01 | 1.15 | 0.44 |
| 209086 | Samd9l | 4.16 | 0.00 | 0.95 | 0.73 |
| 12702 | Socs3 | 4.16 | 0.00 | 2.31 | 0.10 |
| 67749 | 4930583H14Rik | 4.11 | 0.01 | 1.64 | 0.08 |
| 224613 | E030034P13Rik | 4.08 | 0.01 | 0.92 | 0.70 |
| 14991 | H2-M3 | 4.08 | 0.00 | 1.12 | 0.77 |
| 630499 | EG630499 | 4.07 | 0.00 | 1.10 | 0.99 |
| 240327 | EG240327 | 4.06 | 0.00 | 0.80 | 0.53 |
| 20684 | Sp100 | 3.99 | 0.01 | 1.18 | 0.79 |
| 75731 | 5133401N09Rik | 3.95 | 0.00 | 0.96 | 0.66 |
| 107607 | Nod1 | 3.90 | 0.00 | 1.08 | 0.61 |
| 69183 | C1qtnf2 | 3.86 | 0.03 | 1.02 | 0.92 |
| 142980 | Tlr3 | 3.84 | 0.01 | 0.77 | 0.46 |
| 15958 | Ifit2 | 3.79 | 0.00 | 0.74 | 0.10 |
| 21356 | Tapbp | 3.78 | 0.00 | 0.95 | 0.39 |
| 14696 | Gnb4 | 3.74 | 0.00 | 0.81 | 0.37 |
| 12457 | Ccrn4l | 3.74 | 0.00 | 1.42 | 0.29 |
| 30935 | Tor3a | 3.69 | 0.01 | 1.27 | 0.19 |
| 319278 | A230050P20Rik | 3.68 | 0.02 | 1.15 | 0.81 |
| 68252 | A030007L17Rik | 3.66 | 0.00 | 1.16 | 0.66 |
| 100047934 | LOC100047934 | 3.64 | 0.00 | 1.50 | 0.04 |
| 546546 | Serpina3h | 3.62 | 0.01 | 1.43 | 0.87 |
| 100047963 | LOC100047963 | 3.55 | 0.00 | 0.87 | 0.27 |
| 210029 | Metrnl | 3.41 | 0.02 | 1.07 | 0.41 |
| 67809 | 1200015F23Rik | 3.37 | 0.00 | 1.05 | 0.66 |
| 19186 | Psme1 | 3.36 | 0.00 | 1.01 | 0.93 |
| 215418 | Axud1 | 3.31 | 0.02 | 1.23 | 0.36 |
| 19106 | Eif2ak2 | 3.29 | 0.00 | 1.23 | 0.12 |
| 85031 | Pla1a | 3.22 | 0.00 | 1.07 | 0.99 |
| 30959 | Ddx25 | 3.17 | 0.00 | 0.96 | 0.62 |
| 70082 | Lysmd2 | 3.16 | 0.01 | 0.75 | 0.33 |
| 100044190 | LOC100044190 | 3.15 | 0.00 | 0.88 | 0.17 |
| 18769 | Pkig | 3.11 | 0.00 | 1.10 | 0.84 |
| 55991 | Panx1 | 3.09 | 0.00 | 1.14 | 0.58 |
| 621823 | LOC621823 | 3.08 | 0.00 | 0.86 | 0.60 |
| 15944 | Irgm1 | 3.05 | 0.00 | 1.04 | 0.87 |
| 81907 | Tmem108 | 3.01 | 0.01 | 2.90 | 0.12 |
| 16168 | Il15 | 3.01 | 0.00 | 0.84 | 0.59 |
| 229003 | BC006779 | 3.01 | 0.00 | 0.88 | 0.53 |
| 16574 | Kif5c | 2.97 | 0.01 | 0.88 | 0.50 |
| 81018 | Rnf114 | 2.95 | 0.01 | 1.22 | 0.30 |
| 213603 | Slc44a3 | 2.92 | 0.01 | 1.23 | 0.80 |
| 71839 | Osgin1 | 2.92 | 0.01 | 0.91 | 0.78 |
| 12369 | Casp7 | 2.86 | 0.00 | 1.10 | 0.28 |
| 229905 | Ccbl2 | 2.85 | 0.00 | 1.05 | 0.98 |
| 71982 | Snx10 | 2.81 | 0.00 | 1.08 | 0.77 |
| 80281 | Cttnbp2nl | 2.79 | 0.00 | 1.15 | 0.55 |
| 330890 | Piwil4 | 2.77 | 0.00 | 0.57 | 0.26 |
| 72075 | Ogfr | 2.76 | 0.00 | 1.04 | 0.83 |
| 20556 | Slfn2 | 2.72 | 0.01 | 0.86 | 0.58 |
| 80281 | BC003236 | 2.71 | 0.00 | 1.17 | 0.54 |
| 241274 | Pnpla7 | 2.65 | 0.01 | 0.97 | 0.92 |
| 22695 | Zfp36 | 2.63 | 0.01 | 1.34 | 0.03 |
| 22040 | Trex1 | 2.61 | 0.00 | 1.13 | 0.58 |
| 50908 | C1s | 2.61 | 0.03 | 1.21 | 0.72 |
| 77590 | 4631426J05Rik | 2.57 | 0.03 | 1.41 | 0.39 |
| 16164 | Il13ra1 | 2.57 | 0.03 | 1.85 | 0.43 |
| 100608 | Noc4l | 2.56 | 0.00 | 1.29 | 0.09 |
| 547343 | LOC547343 | 2.55 | 0.01 | 0.69 | 0.26 |
| 67179 | Ccdc25 | 2.54 | 0.00 | 0.78 | 0.24 |
| 14962 | Cfb | 2.53 | 0.05 | 0.81 | 0.39 |
| 72297 | B3gnt3 | 2.53 | 0.01 | 1.00 | 0.95 |
| 18854 | Pml | 2.53 | 0.01 | 0.97 | 0.57 |
| 21810 | Tgfbi | 2.52 | 0.04 | 1.36 | 0.88 |
| 71684 | Rbm43 | 2.52 | 0.00 | 1.00 | 0.99 |
| 16476 | Jun | 2.48 | 0.00 | 1.07 | 0.57 |
| 93694 | Clec2d | 2.47 | 0.00 | 1.07 | 0.67 |
| 73167 | Arhgap8 | 2.46 | 0.05 | 1.30 | 0.64 |
| 11569 | Aebp2 | 2.46 | 0.00 | 1.25 | 0.22 |
| 103511 | BB146404 | 2.46 | 0.00 | 0.68 | 0.42 |
| 18519 | Kat2b | 2.46 | 0.00 | 1.34 | 0.12 |
| 67895 | Ppa1 | 2.45 | 0.00 | 1.17 | 0.04 |
| 56417 | Adar | 2.45 | 0.01 | 1.05 | 0.72 |
| 17874 | Myd88 | 2.45 | 0.00 | 1.37 | 0.29 |
| 100045567 | LOC100045567 | 2.43 | 0.00 | 1.15 | 0.01 |
| 109660 | Ctrl | 2.40 | 0.02 | 1.02 | 0.87 |
| 105827 | Amigo2 | 2.40 | 0.01 | 1.53 | 0.30 |
| 13163 | Daxx | 2.38 | 0.00 | 1.00 | 0.99 |
| 242669 | Adc | 2.38 | 0.01 | 1.00 | 0.95 |
| 667977 | EG667977 | 2.38 | 0.01 | 1.10 | 0.87 |
| 235587 | Parp3 | 2.37 | 0.01 | 1.17 | 0.16 |
| 81018 | Zfp313 | 2.36 | 0.01 | 1.18 | 0.43 |
| 66970 | Ssbp2 | 2.34 | 0.01 | 1.06 | 0.85 |
| 16196 | Il7 | 2.33 | 0.01 | 1.05 | 0.86 |
| 16391 | Irf9 | 2.33 | 0.01 | 1.36 | 0.01 |
| 106672 | AI413582 | 2.33 | 0.00 | 0.95 | 0.61 |
| 17524 | Mpp1 | 2.32 | 0.00 | 1.22 | 0.19 |
| 12122 | Bid | 2.28 | 0.00 | 1.02 | 0.92 |
| 18045 | Nfyb | 2.26 | 0.00 | 1.22 | 0.01 |
| 109676 | Ank2 | 2.25 | 0.04 | 0.98 | 0.97 |
| 227659 | Slc2a6 | 2.25 | 0.00 | 0.96 | 0.94 |
| 231712 | Trafd1 | 2.25 | 0.00 | 0.88 | 0.10 |
| 66102 | Cxcl16 | 2.23 | 0.02 | 0.92 | 0.83 |
| 14674 | Gna13 | 2.22 | 0.00 | 1.00 | 0.92 |
| 268749 | Rnf31 | 2.19 | 0.00 | 0.96 | 0.71 |
| 19877 | Rock1 | 2.19 | 0.00 | 1.38 | 0.04 |
| 231287 | Atp10d | 2.18 | 0.01 | 1.13 | 0.62 |
| 14972 | H2-K1 | 2.18 | 0.01 | 1.15 | 0.55 |
| 637082 | LOC637082 | 2.18 | 0.02 | 0.98 | 0.82 |
| 16859 | Lgals9 | 2.17 | 0.01 | 0.99 | 0.95 |
| 67196 | Ube2t | 2.17 | 0.04 | 0.62 | 0.56 |
| 214855 | Arid5a | 2.17 | 0.01 | 1.56 | 0.28 |
| 19039 | Lgals3bp | 2.16 | 0.02 | 0.98 | 0.95 |
| 74338 | Slc6a19 | 2.15 | 0.00 | 0.72 | 0.30 |
| 20304 | Ccl5 | 2.14 | 0.01 | 0.83 | 0.40 |
| 64164 | Ifrg15 | 2.12 | 0.01 | 1.10 | 0.44 |
| 15019 | H2-Q8 | 2.10 | 0.03 | 0.99 | 0.98 |
| 12364 | Casp12 | 2.09 | 0.00 | 1.42 | 0.09 |
| 69399 | 1700025G04Rik | 2.09 | 0.01 | 1.03 | 0.98 |
| 67776 | Vwa5a | 2.08 | 0.00 | 1.10 | 0.56 |
| 381334 | Gal3st2 | 2.08 | 0.00 | 0.89 | 0.84 |
| 19246 | Ptpn1 | 2.06 | 0.00 | 0.91 | 0.76 |
| 68487 | Tmem140 | 2.05 | 0.05 | 0.60 | 0.25 |
| 384309 | Trim56 | 2.05 | 0.00 | 0.97 | 0.85 |
| 18479 | Pak1 | 2.05 | 0.01 | 1.09 | 0.43 |
| 18712 | Pim1 | 2.04 | 0.00 | 0.93 | 0.69 |
| 108116 | Slco3a1 | 2.04 | 0.01 | 1.00 | 0.95 |
| 13852 | Stx2 | 2.03 | 0.02 | 1.28 | 0.06 |
| 18128 | Notch1 | 2.03 | 0.03 | 0.73 | 0.11 |
| 23924 | Katna1 | 2.02 | 0.00 | 1.00 | 1.00 |
| 230119 | Zbtb5 | 2.02 | 0.02 | 0.91 | 0.59 |
| 74137 | Nuak2 | 2.00 | 0.00 | 1.21 | 0.35 |
| 329910 | Acot11 | 0.66 | 0.03 | 0.96 | 0.89 |
| 53858 | Rwdd2b | 0.66 | 0.00 | 0.98 | 0.94 |
| 67475 | Ero1lb | 0.65 | 0.01 | 0.97 | 0.92 |
| 20541 | Slc8a1 | 0.65 | 0.05 | 0.99 | 0.94 |
| 675567 | LOC675567 | 0.65 | 0.02 | 1.07 | 0.52 |
| 27376 | Slc25a10 | 0.65 | 0.01 | 0.86 | 0.35 |
| 77613 | Prss36 | 0.65 | 0.01 | 0.74 | 0.08 |
| 50997 | Mpp2 | 0.65 | 0.03 | 1.01 | 0.93 |
| 67119 | 2510048L02Rik | 0.64 | 0.01 | 1.01 | 0.92 |
| 107986 | Ddb2 | 0.64 | 0.02 | 0.79 | 0.41 |
| 22342 | Lin7b | 0.64 | 0.04 | 0.70 | 0.30 |
| 16780 | Lamb3 | 0.64 | 0.01 | 0.86 | 0.69 |
| 15500 | Hsf2 | 0.64 | 0.02 | 0.90 | 0.71 |
| 21422 | Tcfcp2 | 0.64 | 0.00 | 0.78 | 0.19 |
| 272396 | Tarsl2 | 0.63 | 0.01 | 0.92 | 0.80 |
| 100637 | B230342M21Rik | 0.63 | 0.04 | 0.78 | 0.48 |
| 67370 | Zfp606 | 0.63 | 0.04 | 0.92 | 0.23 |
| 27355 | X99384 | 0.63 | 0.02 | 0.92 | 0.79 |
| 105005 | Fam84a | 0.63 | 0.02 | 0.72 | 0.26 |
| 630537 | Dcpp2 | 0.63 | 0.02 | 0.89 | 0.70 |
| 76467 | Msrb2 | 0.63 | 0.00 | 0.94 | 0.56 |
| 67839 | Gpsm1 | 0.63 | 0.03 | 0.87 | 0.16 |
| 68017 | Ftsj2 | 0.62 | 0.00 | 0.96 | 0.80 |
| 70266 | Ccbl1 | 0.62 | 0.02 | 0.99 | 0.92 |
| 70419 | 2810408A11Rik | 0.62 | 0.05 | 0.75 | 0.13 |
| 17388 | Mmp15 | 0.62 | 0.02 | 0.83 | 0.64 |
| 26412 | Map4k2 | 0.61 | 0.02 | 1.04 | 0.78 |
| 18612 | Etv4 | 0.61 | 0.01 | 1.00 | 0.92 |
| 235497 | LOC235497 | 0.61 | 0.02 | 1.11 | 0.99 |
| 19339 | Rab3a | 0.61 | 0.00 | 0.79 | 0.17 |
| 108097 | Prkab2 | 0.61 | 0.00 | 0.87 | 0.49 |
| 17921 | Myo7a | 0.61 | 0.00 | 1.02 | 0.97 |
| 105171 | Arrdc3 | 0.61 | 0.01 | 0.95 | 0.79 |
| 58226 | Cacna1h | 0.61 | 0.02 | 0.98 | 0.81 |
| 29856 | Smtn | 0.60 | 0.00 | 1.01 | 1.00 |
| 12724 | Clcn2 | 0.60 | 0.00 | 0.91 | 0.50 |
| 20538 | Slc6a2 | 0.60 | 0.02 | 0.85 | 0.49 |
| 213056 | BC049806 | 0.60 | 0.00 | 1.02 | 0.73 |
| 54366 | Ctnnal1 | 0.60 | 0.00 | 0.86 | 0.30 |
| 16398 | Itga2 | 0.60 | 0.03 | 0.81 | 0.87 |
| 236576 | Spry3 | 0.59 | 0.00 | 0.94 | 0.83 |
| 58188 | Vstm2b | 0.58 | 0.01 | 1.03 | 0.92 |
| 20887 | Sult1a1 | 0.58 | 0.03 | 0.73 | 0.06 |
| 22691 | Zscan2 | 0.58 | 0.04 | 0.90 | 0.40 |
| 13426 | Dync1i1 | 0.57 | 0.01 | 1.00 | 0.89 |
| 58242 | Nudt11 | 0.56 | 0.01 | 0.84 | 0.61 |
| 100048332 | LOC100048332 | 0.56 | 0.04 | 0.86 | 0.59 |
| 15925 | Ide | 0.56 | 0.01 | 1.11 | 0.32 |
| 63913 | Fam129a | 0.56 | 0.02 | 1.00 | 0.97 |
| 242642 | Gloxd1 | 0.55 | 0.01 | 0.97 | 0.91 |
| 11522 | Adh1 | 0.54 | 0.02 | 0.75 | 0.62 |
| 545253 | EG545253 | 0.54 | 0.03 | 1.22 | 0.05 |
| 278240 | Spin2 | 0.54 | 0.01 | 0.86 | 0.85 |
| 22402 | Wisp1 | 0.53 | 0.00 | 0.98 | 0.78 |
| 435766 | Tnni3k | 0.52 | 0.00 | 0.91 | 0.79 |
| 15478 | Hs3st3a1 | 0.52 | 0.04 | 0.71 | 0.01 |
| 18121 | Nog | 0.52 | 0.01 | 0.95 | 0.78 |
| 12819 | Col15a1 | 0.51 | 0.03 | 0.84 | 0.95 |
| 235431 | Coro2b | 0.50 | 0.01 | 0.87 | 0.85 |
| 78558 | Htra3 | 0.50 | 0.00 | 0.75 | 0.39 |
| 53412 | Ppp1r3c | 0.49 | 0.00 | 0.74 | 0.08 |
| 70603 | Mutyh | 0.49 | 0.02 | 0.66 | 0.13 |
| 98365 | Slamf9 | 0.46 | 0.01 | 0.99 | 0.95 |
| 68709 | Cilp2 | 0.46 | 0.01 | 0.70 | 0.33 |
| 22061 | Trp63 | 0.46 | 0.02 | 1.11 | 0.44 |
| 89867 | Sec16b | 0.45 | 0.01 | 0.87 | 0.68 |
| 319517 | 6430510M02Rik | 0.43 | 0.00 | 0.90 | 0.79 |
| 14262 | Fmo3 | 0.43 | 0.01 | 0.73 | 0.90 |
| 11302 | Aatk | 0.42 | 0.01 | 0.79 | 0.67 |
| 213391 | Rassf4 | 0.41 | 0.02 | 1.08 | 0.23 |
| 53945 | Slc40a1 | 0.34 | 0.05 | 0.72 | 0.30 |
| 27528 | D0H4S114 | 0.30 | 0.01 | 0.70 | 0.03 |
| 16511 | Kcnh2 | 0.30 | 0.00 | 0.80 | 0.62 |
| 213393 | 8430408G22Rik | 0.29 | 0.00 | 0.89 | 0.94 |
