# Supplementary material for: STAT1-Dependent Signal Integration between IFNγ and TLR4 in Vascular Cells Reflect Pro-Atherogenic Responses in Human Atherosclerosis
Source: PLoS One. 2014 Dec 5;9(12):e113318. doi: 10.1371/journal.pone.0113318 (PMC4257532; doi:10.1371/journal.pone.0113318)
Supplement: Table S3 — List of up and down-regulated genes in response to IFNγ and LPS in VSMCs WT and STAT1−/− . Fold change compared to control. (DOCX) [file pone.0113318.s003.docx]

### Table S3. List of up and down-regulated genes in response to IFNγ and LPS in VSMCs *WT* and *STAT1^-/^*^-^. Fold change compared to control.

| **ENTREZ ID** | **SYMBOL** | **Ratio**  **WT**  **IFNγ + LPS** | **p_value**  **WT**  **IFNγ + LPS** | **Ratio**  **STAT1^-/-^**  **IFNγ + LPS** | **p_value STAT1^-/-^**  **IFNγ + LPS** |
| --- | --- | --- | --- | --- | --- |
| 17329 | Cxcl9 | 2643.50 | 0.00 | 150.73 | 0.00 |
| 15945 | Cxcl10 | 2273.44 | 0.00 | 343.62 | 0.00 |
| 20304 | Ccl5 | 512.54 | 0.00 | 2.14 | 0.01 |
| 58185 | Rsad2 | 509.05 | 0.00 | 22.91 | 0.00 |
| 229898 | Gbp5 | 388.28 | 0.00 | 53.48 | 0.00 |
| 24108 | Ubd | 326.57 | 0.00 | 21.54 | 0.00 |
| 74481 | Batf2 | 314.46 | 0.00 | 134.37 | 0.00 |
| 20296 | Ccl2 | 226.54 | 0.00 | 8.62 | 0.00 |
| 626578 | Gbp10 | 223.24 | 0.00 | 81.44 | 0.00 |
| 16149 | Cd74 | 206.50 | 0.00 | 37.66 | 0.00 |
| 20210 | Saa3 | 206.15 | 0.00 | 2.84 | 0.53 |
| 12981 | Csf2 | 198.96 | 0.00 | 0.37 | 0.13 |
| 60533 | Cd274 | 185.78 | 0.00 | 78.01 | 0.00 |
| 21822 | Tgtp | 169.24 | 0.00 | 182.91 | 0.00 |
| 20306 | Ccl7 | 152.49 | 0.00 | 20.03 | 0.00 |
| 12265 | Ciita | 141.55 | 0.00 | 153.84 | 0.00 |
| 215900 | A630077B13Rik | 137.16 | 0.00 | 43.02 | 0.00 |
| 21939 | Cd40 | 127.14 | 0.00 | 1.03 | 0.86 |
| 22169 | Tyki | 118.80 | 0.00 | 25.09 | 0.00 |
| 21928 | Tnfaip2 | 118.00 | 0.00 | 6.52 | 0.00 |
| 102084 | AI451557 | 115.24 | 0.00 | 77.90 | 0.00 |
| 16145 | Igtp | 114.37 | 0.00 | 104.95 | 0.00 |
| 214854 | Lincr | 108.75 | 0.00 | 1.07 | 0.88 |
| 55932 | Gbp3 | 99.25 | 0.00 | 72.35 | 0.00 |
| 20715 | Serpina3g | 98.56 | 0.00 | 24.76 | 0.00 |
| 547253 | Parp14 | 88.95 | 0.00 | 38.97 | 0.00 |
| 12642 | Ch25h | 83.66 | 0.00 | 1.91 | 0.14 |
| 225594 | LOC225594 | 82.48 | 0.00 | 136.83 | 0.00 |
| 433470 | AA467197 | 79.70 | 0.00 | 47.10 | 0.00 |
| 14961 | H2-Ab1 | 77.85 | 0.00 | 57.18 | 0.00 |
| 435565 | LOC435565 | 77.59 | 0.00 | 92.64 | 0.00 |
| 18126 | Nos2 | 72.15 | 0.00 | 0.71 | 0.00 |
| 24047 | Ccl19 | 68.62 | 0.00 | 1.13 | 0.61 |
| 229900 | Gbp6 | 65.00 | 0.00 | 50.68 | 0.00 |
| 16912 | Psmb9 | 63.87 | 0.00 | 56.81 | 0.00 |
| 17858 | Mx2 | 58.83 | 0.00 | 8.10 | 0.00 |
| 23962 | Oasl2 | 58.10 | 0.00 | 45.08 | 0.00 |
| 80910 | Gpr84 | 57.58 | 0.00 | 0.28 | 0.09 |
| 227659 | Slc2a6 | 53.76 | 0.00 | 2.25 | 0.00 |
| 641240 | LOC641240 | 49.04 | 0.00 | 33.51 | 0.00 |
| 14469 | Gbp2 | 46.90 | 0.00 | 40.01 | 0.00 |
| 54199 | Ccrl2 | 45.93 | 0.00 | 4.18 | 0.00 |
| 14825 | Cxcl1 | 41.78 | 0.01 | 1.09 | 0.84 |
| 16819 | Lcn2 | 39.77 | 0.00 | 0.97 | 0.96 |
| 16913 | Psmb8 | 39.48 | 0.00 | 37.04 | 0.00 |
| 17857 | Mx1 | 39.41 | 0.00 | 3.10 | 0.11 |
| 100048554 | LOC100048554 | 37.77 | 0.00 | 6.12 | 0.01 |
| 15186 | Hdc | 35.87 | 0.00 | 14.78 | 0.00 |
| 74748 | Slamf8 | 35.71 | 0.00 | 24.47 | 0.00 |
| 16362 | Irf1 | 33.07 | 0.00 | 23.54 | 0.00 |
| 54720 | Rcan1 | 32.64 | 0.00 | 1.76 | 0.31 |
| 15953 | Ifi47 | 31.61 | 0.00 | 37.21 | 0.00 |
| 24088 | Tlr2 | 30.94 | 0.00 | 2.13 | 0.11 |
| 23960 | Oas1g | 30.90 | 0.00 | 13.07 | 0.00 |
| 223881 | Rnd1 | 29.63 | 0.01 | 0.83 | 0.79 |
| 100048346 | LOC100048346 | 29.15 | 0.00 | 11.04 | 0.01 |
| 15894 | Icam1 | 28.74 | 0.00 | 4.71 | 0.00 |
| 54123 | Irf7 | 27.36 | 0.00 | 15.67 | 0.00 |
| 24110 | Usp18 | 26.51 | 0.00 | 9.43 | 0.01 |
| 667370 | LOC667370 | 25.77 | 0.00 | 20.43 | 0.00 |
| 219132 | D14Ertd668e | 25.68 | 0.00 | 14.73 | 0.00 |
| 64292 | Ptges | 25.51 | 0.01 | 0.83 | 0.57 |
| 230738 | Zc3h12a | 23.45 | 0.00 | 0.99 | 0.86 |
| 21929 | Tnfaip3 | 23.03 | 0.00 | 0.85 | 0.69 |
| 54396 | Iigp2 | 22.59 | 0.00 | 23.39 | 0.00 |
| 434484 | Sp140 | 22.39 | 0.00 | 11.24 | 0.00 |
| 21354 | Tap1 | 22.03 | 0.00 | 15.09 | 0.00 |
| 15116 | Has1 | 21.84 | 0.00 | 1.40 | 0.30 |
| 19734 | Rgs16 | 21.49 | 0.00 | 1.65 | 0.35 |
| 11910 | Atf3 | 20.29 | 0.00 | 3.73 | 0.06 |
| 231655 | Oasl1 | 20.10 | 0.00 | 1.80 | 0.23 |
| 66102 | Cxcl16 | 19.88 | 0.00 | 2.23 | 0.02 |
| 20344 | Selp | 19.87 | 0.00 | 1.69 | 0.15 |
| 100038882 | LOC100038882 | 19.83 | 0.00 | 12.16 | 0.00 |
| 20556 | Slfn2 | 19.65 | 0.00 | 2.72 | 0.01 |
| 15900 | Irf8 | 19.51 | 0.00 | 6.13 | 0.00 |
| 226695 | Ifi205 | 19.10 | 0.00 | 5.39 | 0.00 |
| 80859 | Nfkbiz | 18.86 | 0.00 | 1.70 | 0.02 |
| 12363 | Casp4 | 18.27 | 0.00 | 6.15 | 0.00 |
| 20846 | Stat1 | 18.17 | 0.00 | 19.26 | 0.00 |
| 546546 | Serpina3h | 18.16 | 0.00 | 3.62 | 0.01 |
| 14969 | H2-Eb1 | 17.33 | 0.00 | 20.66 | 0.00 |
| 19171 | Psmb10 | 17.17 | 0.00 | 13.98 | 0.00 |
| 18037 | Nfkbie | 16.52 | 0.00 | 0.84 | 0.25 |
| 16193 | Il6 | 16.42 | 0.00 | 1.03 | 0.78 |
| 20128 | Trim30 | 16.28 | 0.00 | 11.90 | 0.01 |
| 17392 | Mmp3 | 16.22 | 0.02 | 0.99 | 0.84 |
| 26410 | Map3k8 | 16.14 | 0.00 | 1.28 | 0.10 |
| 20568 | Slpi | 16.05 | 0.00 | 1.27 | 0.61 |
| 23961 | Oas1b | 16.04 | 0.00 | 6.39 | 0.00 |
| 270893 | Tmem132e | 16.03 | 0.03 | 0.20 | 0.10 |
| 16169 | Il15ra | 15.69 | 0.00 | 6.49 | 0.00 |
| 56791 | Ube2l6 | 15.68 | 0.00 | 13.18 | 0.00 |
| 74153 | Ube1l | 15.25 | 0.00 | 13.12 | 0.00 |
| 14204 | Il4i1 | 15.19 | 0.00 | 0.93 | 0.79 |
| 17750 | Mt2 | 14.96 | 0.00 | 1.60 | 0.11 |
| 15891 | Ibsp | 14.94 | 0.00 | 0.84 | 0.54 |
| 22029 | Traf1 | 14.92 | 0.00 | 0.81 | 0.41 |
| 14962 | Cfb | 14.57 | 0.00 | 2.53 | 0.05 |
| 22271 | Upp1 | 14.45 | 0.00 | 2.67 | 0.08 |
| 213233 | Tapbpl | 13.93 | 0.00 | 10.80 | 0.00 |
| 192656 | Ripk2 | 13.85 | 0.00 | 2.18 | 0.00 |
| 12263 | C2 | 13.49 | 0.00 | 9.44 | 0.00 |
| 80861 | Dhx58 | 13.32 | 0.00 | 7.02 | 0.00 |
| 12904 | Crabp2 | 13.24 | 0.00 | 13.03 | 0.00 |
| 19288 | Ptx3 | 13.06 | 0.01 | 2.07 | 0.44 |
| 15959 | Ifit3 | 13.02 | 0.00 | 10.42 | 0.00 |
| 18035 | Nfkbia | 13.02 | 0.00 | 1.05 | 0.87 |
| 20821 | Trim21 | 12.89 | 0.00 | 9.87 | 0.00 |
| 21934 | Tnfrsf11a | 12.85 | 0.01 | 0.20 | 0.09 |
| 67775 | Rtp4 | 12.51 | 0.00 | 8.48 | 0.00 |
| 100047934 | LOC100047934 | 12.51 | 0.00 | 3.64 | 0.00 |
| 56045 | Samhd1 | 12.37 | 0.00 | 11.54 | 0.00 |
| 14528 | Gch1 | 12.31 | 0.00 | 1.65 | 0.02 |
| 26570 | Slc7a11 | 12.31 | 0.00 | 0.91 | 0.77 |
| 74190 | 1200009I06Rik | 12.30 | 0.00 | 1.70 | 0.16 |
| 240354 | Malt1 | 12.22 | 0.00 | 1.41 | 0.24 |
| 27056 | Irf5 | 12.12 | 0.00 | 1.97 | 0.05 |
| 16174 | Il18rap | 11.96 | 0.01 | 6.04 | 0.02 |
| 12061 | Bdkrb1 | 11.96 | 0.01 | 0.42 | 0.10 |
| 15114 | Hap1 | 11.89 | 0.00 | 4.03 | 0.01 |
| 100048556 | LOC100048556 | 11.86 | 0.00 | 1.05 | 0.87 |
| 574428 | Zmynd15 | 11.65 | 0.00 | 1.20 | 0.64 |
| 69146 | Gsdmdc1 | 11.57 | 0.00 | 4.72 | 0.00 |
| 11541 | Adora2b | 11.54 | 0.00 | 1.01 | 0.94 |
| 12700 | Cish | 11.44 | 0.00 | 1.81 | 0.04 |
| 12051 | Bcl3 | 11.28 | 0.00 | 1.97 | 0.03 |
| 620913 | OTTMUSG00000005523 | 11.13 | 0.00 | 10.97 | 0.00 |
| 17386 | Mmp13 | 11.08 | 0.00 | 0.65 | 0.31 |
| 18591 | Pdgfb | 10.96 | 0.00 | 0.90 | 0.68 |
| 13123 | Cyp7b1 | 10.75 | 0.00 | 0.69 | 0.04 |
| 71898 | Apol9b | 10.68 | 0.00 | 9.67 | 0.00 |
| 30935 | Tor3a | 10.51 | 0.00 | 3.69 | 0.01 |
| 229003 | BC006779 | 10.47 | 0.00 | 3.01 | 0.00 |
| 12457 | Ccrn4l | 10.37 | 0.00 | 3.74 | 0.00 |
| 22329 | Vcam1 | 10.35 | 0.01 | 1.66 | 0.30 |
| 14960 | H2-Aa | 10.27 | 0.02 | 7.76 | 0.03 |
| 73167 | Arhgap8 | 10.21 | 0.00 | 2.46 | 0.05 |
| 630499 | EG630499 | 9.97 | 0.00 | 4.07 | 0.00 |
| 15930 | Indo | 9.88 | 0.00 | 7.80 | 0.00 |
| 218454 | Lhfpl2 | 9.47 | 0.00 | 0.91 | 0.55 |
| 243771 | Parp12 | 9.47 | 0.00 | 8.35 | 0.00 |
| 14612 | Gja4 | 9.35 | 0.00 | 1.25 | 0.80 |
| 22436 | Xdh | 9.26 | 0.00 | 5.81 | 0.00 |
| 15958 | Ifit2 | 9.18 | 0.00 | 3.79 | 0.00 |
| 71803 | Slc25a18 | 8.90 | 0.00 | 0.20 | 0.07 |
| 11988 | Slc7a2 | 8.86 | 0.00 | 0.91 | 0.77 |
| 20312 | Cx3cl1 | 8.78 | 0.00 | 1.89 | 0.00 |
| 22040 | Trex1 | 8.75 | 0.00 | 2.61 | 0.00 |
| 60440 | AW111922 | 8.74 | 0.00 | 7.59 | 0.00 |
| 215418 | Axud1 | 8.70 | 0.00 | 3.31 | 0.02 |
| 23969 | Pacsin1 | 8.63 | 0.00 | 8.42 | 0.00 |
| 21355 | Tap2 | 8.50 | 0.00 | 7.31 | 0.00 |
| 16168 | Il15 | 8.50 | 0.00 | 3.01 | 0.00 |
| 12916 | Crem | 8.43 | 0.00 | 4.85 | 0.00 |
| 20684 | Sp100 | 8.32 | 0.00 | 3.99 | 0.01 |
| 12703 | Socs1 | 8.27 | 0.00 | 5.19 | 0.00 |
| 74568 | Mlkl | 8.25 | 0.00 | 5.91 | 0.00 |
| 12258 | Serping1 | 8.19 | 0.00 | 5.80 | 0.00 |
| 15019 | H2-Q8 | 7.96 | 0.00 | 2.10 | 0.03 |
| 12362 | Casp1 | 7.87 | 0.00 | 5.56 | 0.02 |
| 22375 | Wars | 7.86 | 0.00 | 6.22 | 0.00 |
| 57875 | Angptl4 | 7.75 | 0.00 | 1.25 | 0.67 |
| 15018 | H2-Q7 | 7.55 | 0.00 | 5.59 | 0.00 |
| 56489 | Ikbke | 7.47 | 0.00 | 1.02 | 0.46 |
| 100047963 | LOC100047963 | 7.36 | 0.00 | 3.55 | 0.00 |
| 13654 | Egr2 | 7.32 | 0.00 | 1.74 | 0.23 |
| 16803 | Lbp | 7.19 | 0.00 | 0.88 | 0.61 |
| 15976 | Ifnar2 | 7.17 | 0.00 | 1.85 | 0.00 |
| 19698 | Relb | 7.00 | 0.00 | 0.96 | 0.70 |
| 13163 | Daxx | 6.98 | 0.00 | 2.38 | 0.00 |
| 20847 | Stat2 | 6.98 | 0.00 | 7.06 | 0.00 |
| 107607 | Nod1 | 6.97 | 0.00 | 3.90 | 0.00 |
| 328561 | 9130218O11Rik | 6.85 | 0.00 | 5.21 | 0.00 |
| 77590 | 4631426J05Rik | 6.80 | 0.01 | 2.57 | 0.03 |
| 12702 | Socs3 | 6.66 | 0.00 | 4.16 | 0.00 |
| 68252 | A030007L17Rik | 6.48 | 0.00 | 3.66 | 0.00 |
| 70110 | Ifi35 | 6.44 | 0.00 | 5.22 | 0.00 |
| 11639 | Ak3l1 | 6.43 | 0.00 | 1.68 | 0.25 |
| 209387 | AI451617 | 6.42 | 0.00 | 4.35 | 0.00 |
| 18033 | Nfkb1 | 6.41 | 0.00 | 1.12 | 0.60 |
| 74155 | Errfi1 | 6.41 | 0.00 | 1.37 | 0.04 |
| 57783 | Tnip1 | 6.35 | 0.00 | 1.08 | 0.63 |
| 14999 | H2-DMb1 | 6.30 | 0.00 | 4.78 | 0.00 |
| 12122 | Bid | 6.26 | 0.00 | 2.28 | 0.00 |
| 637082 | LOC637082 | 6.22 | 0.00 | 2.18 | 0.02 |
| 105841 | Dennd3 | 6.21 | 0.01 | 1.60 | 0.20 |
| 209086 | Samd9l | 6.17 | 0.00 | 4.16 | 0.00 |
| 15024 | H2-T10 | 6.14 | 0.03 | 1.86 | 0.34 |
| 70012 | Ccdc21 | 6.11 | 0.02 | 1.88 | 0.23 |
| 21356 | Tapbp | 6.11 | 0.00 | 3.78 | 0.00 |
| 319278 | A230050P20Rik | 5.99 | 0.01 | 3.68 | 0.02 |
| 108116 | Slco3a1 | 5.91 | 0.00 | 2.04 | 0.01 |
| 16164 | Il13ra1 | 5.81 | 0.00 | 2.57 | 0.03 |
| 73167 | 3110043J09Rik | 5.77 | 0.00 | 1.45 | 0.20 |
| 12369 | Casp7 | 5.70 | 0.00 | 2.86 | 0.00 |
| 12266 | C3 | 5.67 | 0.00 | 1.40 | 0.07 |
| 667977 | EG667977 | 5.66 | 0.00 | 2.38 | 0.01 |
| 80885 | Gpr109a | 5.63 | 0.00 | 0.09 | 0.00 |
| 12006 | Axin2 | 5.58 | 0.00 | 4.91 | 0.00 |
| 18991 | Pou3f1 | 5.54 | 0.00 | 1.90 | 0.14 |
| 50908 | C1s | 5.52 | 0.00 | 2.61 | 0.03 |
| 213603 | Slc44a3 | 5.48 | 0.00 | 2.92 | 0.01 |
| 14991 | H2-M3 | 5.46 | 0.00 | 4.08 | 0.00 |
| 12013 | Bach1 | 5.38 | 0.00 | 1.56 | 0.02 |
| 224794 | Enpp4 | 5.35 | 0.00 | 1.53 | 0.12 |
| 11535 | Adm | 5.34 | 0.00 | 0.75 | 0.06 |
| 17069 | Ly6e | 5.33 | 0.01 | 4.17 | 0.01 |
| 230073 | Ddx58 | 5.31 | 0.00 | 4.64 | 0.00 |
| 15377 | Foxa3 | 5.31 | 0.04 | 0.69 | 0.41 |
| 243910 | Nfkbid | 5.31 | 0.00 | 1.28 | 0.19 |
| 18034 | Nfkb2 | 5.29 | 0.00 | 0.96 | 0.64 |
| 217837 | Itpk1 | 5.29 | 0.00 | 1.12 | 0.28 |
| 207259 | Zbtb7c | 5.24 | 0.00 | 1.33 | 0.51 |
| 240913 | Adamts4 | 5.24 | 0.01 | 0.64 | 0.17 |
| 142980 | Tlr3 | 5.23 | 0.00 | 3.84 | 0.01 |
| 15040 | H2-T23 | 5.22 | 0.00 | 5.04 | 0.00 |
| 17874 | Myd88 | 5.18 | 0.00 | 2.45 | 0.00 |
| 69550 | Bst2 | 5.16 | 0.00 | 5.54 | 0.00 |
| 14998 | H2-DMa | 5.14 | 0.00 | 6.59 | 0.00 |
| 14972 | H2-K1 | 5.13 | 0.00 | 2.18 | 0.01 |
| 68709 | Cilp2 | 5.12 | 0.01 | 0.46 | 0.01 |
| 67809 | 1200015F23Rik | 5.11 | 0.00 | 3.37 | 0.00 |
| 17067 | Ly6c1 | 5.05 | 0.00 | 4.95 | 0.00 |
| 67338 | Rffl | 5.01 | 0.00 | 0.72 | 0.06 |
| 21810 | Tgfbi | 5.01 | 0.01 | 2.52 | 0.04 |
| 80287 | Apobec3 | 4.97 | 0.02 | 2.65 | 0.07 |
| 239559 | A4galt | 4.90 | 0.03 | 0.99 | 0.88 |
| 21942 | Tnfrsf9 | 4.89 | 0.01 | 0.95 | 0.87 |
| 66892 | Eif4e3 | 4.88 | 0.00 | 4.83 | 0.00 |
| 56628 | LOC56628 | 4.86 | 0.04 | 1.76 | 0.19 |
| 20308 | Ccl9 | 4.83 | 0.01 | 0.73 | 0.35 |
| 19106 | Eif2ak2 | 4.78 | 0.00 | 3.29 | 0.00 |
| 214855 | Arid5a | 4.77 | 0.00 | 2.17 | 0.01 |
| 22038 | Plscr1 | 4.75 | 0.00 | 1.36 | 0.11 |
| 231830 | Micall2 | 4.74 | 0.00 | 0.83 | 0.34 |
| 171209 | Accn3 | 4.72 | 0.01 | 0.31 | 0.07 |
| 18712 | Pim1 | 4.70 | 0.00 | 2.04 | 0.00 |
| 235442 | Rab8b | 4.67 | 0.00 | 1.20 | 0.17 |
| 14696 | Gnb4 | 4.67 | 0.00 | 3.74 | 0.00 |
| 381058 | Unc93a | 4.66 | 0.00 | 2.58 | 0.05 |
| 224613 | E030034P13Rik | 4.65 | 0.00 | 4.08 | 0.01 |
| 73914 | Irak3 | 4.62 | 0.00 | 1.00 | 0.98 |
| 72747 | 2810439F02Rik | 4.52 | 0.00 | 1.14 | 0.12 |
| 21460 | Tcp10a | 4.43 | 0.00 | 0.49 | 0.01 |
| 18036 | Nfkbib | 4.43 | 0.05 | 1.18 | 0.64 |
| 11797 | Birc2 | 4.40 | 0.00 | 1.25 | 0.03 |
| 16818 | Lck | 4.40 | 0.02 | 1.65 | 0.38 |
| 59027 | Nampt | 4.39 | 0.00 | 5.23 | 0.00 |
| 217203 | Tmem106a | 4.38 | 0.00 | 4.19 | 0.00 |
| 547343 | LOC547343 | 4.38 | 0.00 | 2.55 | 0.01 |
| 229905 | Ccbl2 | 4.36 | 0.00 | 2.85 | 0.00 |
| 16909 | Lmo2 | 4.34 | 0.00 | 1.41 | 0.46 |
| 208846 | Daam1 | 4.33 | 0.00 | 1.49 | 0.01 |
| 381269 | Mreg | 4.32 | 0.00 | 1.33 | 0.41 |
| 100046232 | LOC100046232 | 4.29 | 0.00 | 1.64 | 0.02 |
| 63872 | Zfp296 | 4.28 | 0.00 | 1.61 | 0.05 |
| 23924 | Katna1 | 4.21 | 0.00 | 2.02 | 0.00 |
| 67916 | Ppap2b | 4.19 | 0.01 | 1.24 | 0.54 |
| 100047200 | LOC100047200 | 4.17 | 0.02 | 0.62 | 0.46 |
| 75731 | 5133401N09Rik | 4.17 | 0.00 | 3.95 | 0.00 |
| 109660 | Ctrl | 4.14 | 0.01 | 2.40 | 0.02 |
| 54720 | Dscr1 | 4.13 | 0.00 | 0.97 | 0.81 |
| 14102 | Fas | 4.12 | 0.00 | 1.65 | 0.00 |
| 67749 | 4930583H14Rik | 4.05 | 0.01 | 4.11 | 0.01 |
| 75379 | 4930599N23Rik | 4.04 | 0.04 | 2.10 | 0.15 |
| 100044190 | LOC100044190 | 4.00 | 0.00 | 3.15 | 0.00 |
| 15937 | Ier3 | 3.96 | 0.00 | 1.22 | 0.10 |
| 22695 | Zfp36 | 3.96 | 0.00 | 2.63 | 0.01 |
| 20656 | Sod2 | 3.95 | 0.00 | 1.40 | 0.01 |
| 84652 | Drctnnb1a | 3.94 | 0.00 | 1.35 | 0.00 |
| 71684 | Rbm43 | 3.93 | 0.00 | 2.52 | 0.00 |
| 76408 | Abcc3 | 3.92 | 0.00 | 0.90 | 0.64 |
| 71982 | Snx10 | 3.88 | 0.00 | 2.81 | 0.00 |
| 58244 | Stx6 | 3.87 | 0.00 | 1.07 | 0.57 |
| 55991 | Panx1 | 3.86 | 0.00 | 3.09 | 0.00 |
| 328949 | Mcc | 3.85 | 0.02 | 0.85 | 0.54 |
| 81018 | Rnf114 | 3.85 | 0.00 | 2.95 | 0.01 |
| 12505 | Cd44 | 3.84 | 0.00 | 1.15 | 0.29 |
| 434204 | Whdc1 | 3.83 | 0.00 | 1.66 | 0.00 |
| 240327 | EG240327 | 3.83 | 0.00 | 4.06 | 0.00 |
| 19186 | Psme1 | 3.82 | 0.00 | 3.36 | 0.00 |
| 30953 | Schip1 | 3.81 | 0.00 | 1.94 | 0.01 |
| 231713 | C330023M02Rik | 3.80 | 0.00 | 1.67 | 0.00 |
| 52552 | Parp8 | 3.75 | 0.00 | 1.08 | 0.73 |
| 72075 | Ogfr | 3.74 | 0.00 | 2.76 | 0.00 |
| 227731 | Slc25a25 | 3.72 | 0.00 | 0.86 | 0.51 |
| 74646 | Spsb1 | 3.70 | 0.00 | 1.75 | 0.06 |
| 85031 | Pla1a | 3.66 | 0.00 | 3.22 | 0.00 |
| 239650 | AI836003 | 3.65 | 0.04 | 1.02 | 0.96 |
| 226419 | Dyrk3 | 3.64 | 0.00 | 1.27 | 0.53 |
| 209588 | Sectm1a | 3.62 | 0.01 | 1.21 | 0.60 |
| 20295 | Ccl17 | 3.62 | 0.04 | 0.89 | 0.75 |
| 71712 | 1200002N14Rik | 3.62 | 0.00 | 1.53 | 0.13 |
| 170737 | Znrf1 | 3.58 | 0.00 | 1.53 | 0.00 |
| 100045567 | LOC100045567 | 3.57 | 0.00 | 2.43 | 0.00 |
| 56417 | Adar | 3.56 | 0.00 | 2.45 | 0.01 |
| 11810 | Apobec1 | 3.56 | 0.00 | 1.38 | 0.18 |
| 330890 | Piwil4 | 3.54 | 0.00 | 2.77 | 0.00 |
| 18174 | Slc11a2 | 3.54 | 0.00 | 1.09 | 0.67 |
| 232533 | Stk38l | 3.52 | 0.00 | 1.83 | 0.01 |
| 12977 | Csf1 | 3.47 | 0.01 | 1.63 | 0.07 |
| 15251 | Hif1a | 3.46 | 0.03 | 1.14 | 0.67 |
| 18081 | Ninj1 | 3.45 | 0.01 | 0.94 | 0.76 |
| 16476 | Jun | 3.45 | 0.01 | 2.48 | 0.00 |
| 100608 | Noc4l | 3.45 | 0.00 | 2.56 | 0.00 |
| 93694 | Clec2d | 3.44 | 0.00 | 2.47 | 0.00 |
| 16918 | Mycl1 | 3.42 | 0.02 | 0.93 | 0.73 |
| 15944 | Irgm1 | 3.40 | 0.00 | 3.05 | 0.00 |
| 18573 | Pde1a | 3.40 | 0.00 | 1.87 | 0.00 |
| 12192 | Zfp36l1 | 3.37 | 0.00 | 1.13 | 0.53 |
| 381845 | 2310014L17Rik | 3.36 | 0.00 | 1.05 | 0.84 |
| 18854 | Pml | 3.36 | 0.00 | 2.53 | 0.01 |
| 228775 | Trib3 | 3.35 | 0.00 | 1.82 | 0.01 |
| 80281 | Cttnbp2nl | 3.35 | 0.00 | 2.79 | 0.00 |
| 52118 | Pvr | 3.34 | 0.00 | 1.26 | 0.07 |
| 16477 | Junb | 3.34 | 0.01 | 1.04 | 0.97 |
| 11569 | Aebp2 | 3.31 | 0.00 | 2.46 | 0.00 |
| 17384 | Mmp10 | 3.30 | 0.01 | 0.70 | 0.42 |
| 14313 | Fst | 3.30 | 0.00 | 1.83 | 0.03 |
| 20442 | St3gal1 | 3.28 | 0.01 | 0.92 | 0.75 |
| 107569 | Nt5c3 | 3.28 | 0.00 | 1.41 | 0.17 |
| 14674 | Gna13 | 3.27 | 0.00 | 2.22 | 0.00 |
| 229599 | Gm129 | 3.26 | 0.00 | 1.16 | 0.23 |
| 80281 | BC003236 | 3.25 | 0.00 | 2.71 | 0.00 |
| 78781 | Zc3hav1 | 3.25 | 0.01 | 1.88 | 0.06 |
| 23871 | Ets1 | 3.24 | 0.00 | 1.20 | 0.05 |
| 16423 | Cd47 | 3.23 | 0.00 | 1.70 | 0.01 |
| 30959 | Ddx25 | 3.23 | 0.01 | 3.17 | 0.00 |
| 230119 | Zbtb5 | 3.21 | 0.01 | 2.02 | 0.02 |
| 14584 | Gfpt2 | 3.20 | 0.01 | 0.78 | 0.43 |
| 67102 | D16Ertd472e | 3.19 | 0.00 | 1.45 | 0.02 |
| 14008 | Etsrp71 | 3.18 | 0.00 | 0.48 | 0.15 |
| 67712 | Slc25a37 | 3.17 | 0.01 | 1.33 | 0.18 |
| 68487 | Tmem140 | 3.17 | 0.01 | 2.05 | 0.05 |
| 57444 | Isg20 | 3.14 | 0.05 | 1.90 | 0.18 |
| 100048858 | LOC100048858 | 3.13 | 0.00 | 1.11 | 0.47 |
| 19271 | Ptprj | 3.13 | 0.00 | 0.77 | 0.37 |
| 83671 | Sytl2 | 3.12 | 0.00 | 0.64 | 0.08 |
| 11501 | Adam8 | 3.10 | 0.05 | 1.07 | 0.86 |
| 67895 | Ppa1 | 3.09 | 0.00 | 2.45 | 0.00 |
| 246696 | Slc25a28 | 3.09 | 0.00 | 1.88 | 0.00 |
| 207565 | Camkk2 | 3.08 | 0.00 | 0.93 | 0.46 |
| 241274 | Pnpla7 | 3.07 | 0.01 | 2.65 | 0.01 |
| 56075 | Pdss1 | 3.06 | 0.00 | 1.64 | 0.03 |
| 18769 | Pkig | 3.06 | 0.00 | 3.11 | 0.00 |
| 214359 | Tmem51 | 3.04 | 0.01 | 1.61 | 0.16 |
| 53608 | Map3k6 | 3.04 | 0.02 | 0.87 | 0.53 |
| 22341 | Vegfc | 3.03 | 0.03 | 2.17 | 0.23 |
| 81018 | Zfp313 | 3.01 | 0.00 | 2.36 | 0.01 |
| 67179 | Ccdc25 | 2.98 | 0.00 | 2.54 | 0.00 |
| 21817 | Tgm2 | 2.98 | 0.00 | 1.98 | 0.02 |
| 21950 | Tnfsf9 | 2.97 | 0.02 | 0.90 | 0.80 |
| 71704 | Arhgef3 | 2.95 | 0.03 | 1.01 | 0.99 |
| 380711 | Garnl4 | 2.93 | 0.00 | 1.61 | 0.04 |
| 11852 | Rhob | 2.91 | 0.00 | 1.37 | 0.07 |
| 268749 | Rnf31 | 2.91 | 0.00 | 2.19 | 0.00 |
| 56437 | Rrad | 2.89 | 0.01 | 0.94 | 0.89 |
| 96979 | Ptges2 | 2.88 | 0.00 | 0.91 | 0.41 |
| 230837 | Asap3 | 2.88 | 0.02 | 1.74 | 0.11 |
| 16574 | Kif5c | 2.88 | 0.01 | 2.97 | 0.01 |
| 67664 | Rnf125 | 2.86 | 0.00 | 0.67 | 0.20 |
| 216549 | Aftph | 2.86 | 0.00 | 1.37 | 0.05 |
| 80290 | Gpr146 | 2.86 | 0.04 | 2.32 | 0.08 |
| 11828 | Aqp3 | 2.85 | 0.02 | 1.06 | 0.70 |
| 27060 | Tcirg1 | 2.84 | 0.00 | 1.47 | 0.08 |
| 19222 | Ptgir | 2.83 | 0.01 | 0.83 | 0.44 |
| 76889 | Adck4 | 2.83 | 0.01 | 1.96 | 0.01 |
| 231712 | Trafd1 | 2.83 | 0.00 | 2.25 | 0.00 |
| 387609 | Zhx2 | 2.82 | 0.01 | 0.76 | 0.03 |
| 69183 | C1qtnf2 | 2.81 | 0.04 | 3.86 | 0.03 |
| 66970 | Ssbp2 | 2.81 | 0.01 | 2.34 | 0.01 |
| 16859 | Lgals9 | 2.78 | 0.00 | 2.17 | 0.01 |
| 246256 | Fcgr4 | 2.77 | 0.01 | 1.86 | 0.05 |
| 19039 | Lgals3bp | 2.77 | 0.00 | 2.16 | 0.02 |
| 320508 | Cachd1 | 2.76 | 0.00 | 1.07 | 0.63 |
| 13660 | Ehd1 | 2.76 | 0.02 | 1.08 | 0.51 |
| 21815 | Tgif1 | 2.76 | 0.00 | 1.52 | 0.04 |
| 384309 | Trim56 | 2.75 | 0.00 | 2.05 | 0.00 |
| 213053 | Slc39a14 | 2.75 | 0.00 | 0.84 | 0.19 |
| 621823 | LOC621823 | 2.74 | 0.00 | 3.08 | 0.00 |
| 100046255 | LOC100046255 | 2.74 | 0.04 | 0.85 | 0.51 |
| 72297 | B3gnt3 | 2.74 | 0.00 | 2.53 | 0.01 |
| 70082 | Lysmd2 | 2.72 | 0.03 | 3.16 | 0.01 |
| 27966 | Rrp9 | 2.72 | 0.00 | 1.55 | 0.02 |
| 102626 | Mapkapk3 | 2.72 | 0.00 | 1.09 | 0.52 |
| 93692 | Glrx | 2.71 | 0.00 | 1.16 | 0.36 |
| 12796 | Camp | 2.70 | 0.03 | 0.48 | 0.01 |
| 17164 | Mapkapk2 | 2.69 | 0.01 | 1.04 | 0.69 |
| 74165 | Fbxl22 | 2.68 | 0.01 | 0.60 | 0.19 |
| 71956 | Rnf135 | 2.68 | 0.00 | 1.50 | 0.01 |
| 71683 | Gypc | 2.67 | 0.00 | 0.97 | 0.92 |
| 19697 | Rela | 2.67 | 0.00 | 1.23 | 0.17 |
| 64164 | Ifrg15 | 2.66 | 0.00 | 2.12 | 0.01 |
| 12475 | Cd14 | 2.66 | 0.00 | 1.25 | 0.24 |
| 15898 | Icam5 | 2.65 | 0.05 | 0.54 | 0.12 |
| 58194 | Sh3kbp1 | 2.64 | 0.00 | 1.06 | 0.33 |
| 16363 | Irf2 | 2.64 | 0.00 | 1.83 | 0.00 |
| 12364 | Casp12 | 2.64 | 0.02 | 2.09 | 0.00 |
| 71893 | Noxo1 | 2.64 | 0.01 | 1.26 | 0.03 |
| 68591 | Mocos | 2.60 | 0.00 | 1.91 | 0.00 |
| 67844 | Rab32 | 2.59 | 0.00 | 1.07 | 0.57 |
| 105203 | BC016423 | 2.57 | 0.00 | 1.46 | 0.10 |
| 319765 | Igf2bp2 | 2.57 | 0.00 | 1.28 | 0.06 |
| 235587 | Parp3 | 2.57 | 0.00 | 2.37 | 0.01 |
| 85305 | Kars | 2.55 | 0.00 | 1.90 | 0.00 |
| 66943 | Pqlc1 | 2.55 | 0.01 | 1.54 | 0.00 |
| 69065 | Chac1 | 2.54 | 0.01 | 1.12 | 0.35 |
| 17190 | Mbd1 | 2.54 | 0.00 | 0.94 | 0.61 |
| 110532 | Adarb1 | 2.53 | 0.01 | 0.86 | 0.44 |
| 77056 | Tmco4 | 2.53 | 0.04 | 1.94 | 0.10 |
| 20910 | Stxbp1 | 2.53 | 0.00 | 1.70 | 0.00 |
| 58203 | Zbp1 | 2.50 | 0.00 | 1.63 | 0.00 |
| 21664 | Phlda1 | 2.50 | 0.00 | 1.10 | 0.24 |
| 216161 | Sbno2 | 2.50 | 0.01 | 1.22 | 0.24 |
| 66853 | Pnpla2 | 2.50 | 0.00 | 1.96 | 0.00 |
| 228836 | Dlgap4 | 2.49 | 0.01 | 1.07 | 0.61 |
| 19650 | Rbl1 | 2.49 | 0.00 | 1.99 | 0.02 |
| 11641 | Akap2 | 2.48 | 0.00 | 1.08 | 0.64 |
| 54721 | Tyk2 | 2.48 | 0.01 | 1.07 | 0.12 |
| 54445 | Unc93b1 | 2.47 | 0.01 | 2.05 | 0.05 |
| 24099 | Tnfsf13b | 2.47 | 0.02 | 1.82 | 0.20 |
| 226442 | Zfp281 | 2.46 | 0.00 | 1.36 | 0.02 |
| 53312 | Nub1 | 2.46 | 0.00 | 1.85 | 0.00 |
| 26464 | Vnn3 | 2.44 | 0.03 | 0.75 | 0.33 |
| 105827 | Amigo2 | 2.43 | 0.02 | 2.40 | 0.01 |
| 108682 | Gpt2 | 2.41 | 0.03 | 0.91 | 0.65 |
| 22033 | Traf5 | 2.41 | 0.00 | 1.24 | 0.23 |
| 73379 | Dcbld2 | 2.39 | 0.02 | 0.99 | 0.92 |
| 269113 | Nup54 | 2.39 | 0.00 | 1.23 | 0.34 |
| 108960 | Irak2 | 2.39 | 0.02 | 1.52 | 0.05 |
| 18128 | Notch1 | 2.39 | 0.02 | 2.03 | 0.03 |
| 117171 | 1110038F14Rik | 2.39 | 0.00 | 1.90 | 0.01 |
| 18950 | Pnp1 | 2.39 | 0.01 | 1.13 | 0.65 |
| 230484 | Usp1 | 2.38 | 0.00 | 1.66 | 0.05 |
| 20525 | Slc2a1 | 2.38 | 0.00 | 1.25 | 0.14 |
| 231452 | Sdad1 | 2.38 | 0.00 | 1.13 | 0.21 |
| 217370 | BC017643 | 2.38 | 0.00 | 1.57 | 0.01 |
| 17873 | Gadd45b | 2.37 | 0.02 | 1.05 | 0.63 |
| 226823 | Kctd3 | 2.36 | 0.03 | 1.69 | 0.07 |
| 74338 | Slc6a19 | 2.36 | 0.00 | 2.15 | 0.00 |
| 59028 | Rcl1 | 2.36 | 0.00 | 0.95 | 0.64 |
| 100044934 | LOC100044934 | 2.36 | 0.02 | 1.27 | 0.23 |
| 23872 | Ets2 | 2.35 | 0.00 | 0.86 | 0.46 |
| 210808 | 9030625A04Rik | 2.35 | 0.02 | 0.89 | 0.60 |
| 106869 | Tnfaip8 | 2.35 | 0.00 | 1.31 | 0.17 |
| 20511 | Slc1a2 | 2.34 | 0.02 | 0.69 | 0.21 |
| 102294 | Cyp4v3 | 2.34 | 0.03 | 1.56 | 0.11 |
| 14739 | Edg5 | 2.34 | 0.03 | 1.10 | 0.27 |
| 54383 | Phc2 | 2.34 | 0.01 | 1.57 | 0.04 |
| 170460 | Stard5 | 2.34 | 0.04 | 1.18 | 0.54 |
| 18669 | Abcb1b | 2.34 | 0.01 | 1.06 | 0.24 |
| 217578 | Baz1a | 2.33 | 0.02 | 1.68 | 0.09 |
| 20352 | Sema4b | 2.33 | 0.01 | 1.28 | 0.09 |
| 71733 | Susd2 | 2.32 | 0.02 | 0.79 | 0.36 |
| 18414 | Osmr | 2.32 | 0.00 | 1.04 | 0.78 |
| 414801 | Itprip | 2.32 | 0.00 | 1.46 | 0.02 |
| 208263 | Tor1aip1 | 2.32 | 0.00 | 1.90 | 0.00 |
| 22670 | Trim26 | 2.32 | 0.00 | 1.65 | 0.00 |
| 140780 | Bmp2k | 2.31 | 0.01 | 1.01 | 0.88 |
| 193742 | Bat5 | 2.31 | 0.00 | 1.93 | 0.00 |
| 14941 | Gzmd | 2.30 | 0.04 | 0.47 | 0.14 |
| 64143 | Ralb | 2.30 | 0.00 | 1.10 | 0.13 |
| 71839 | Osgin1 | 2.30 | 0.03 | 2.92 | 0.01 |
| 67800 | Dgat2 | 2.30 | 0.01 | 0.77 | 0.44 |
| 67245 | Peli1 | 2.29 | 0.00 | 1.34 | 0.05 |
| 11853 | Rhoc | 2.29 | 0.01 | 1.67 | 0.02 |
| 16391 | Irf9 | 2.29 | 0.01 | 2.33 | 0.01 |
| 73835 | Ifitm5 | 2.28 | 0.04 | 0.58 | 0.29 |
| 74868 | Tmem65 | 2.28 | 0.02 | 1.48 | 0.02 |
| 102162 | Taf5l | 2.28 | 0.00 | 1.57 | 0.01 |
| 70928 | Trim69 | 2.26 | 0.01 | 1.25 | 0.23 |
| 80285 | BC003281 | 2.26 | 0.00 | 1.46 | 0.25 |
| 387524 | Znrf2 | 2.26 | 0.00 | 1.67 | 0.00 |
| 50772 | Mapk6 | 2.25 | 0.00 | 1.11 | 0.53 |
| 545389 | Cep170 | 2.25 | 0.02 | 1.58 | 0.02 |
| 384009 | Glipr2 | 2.25 | 0.00 | 1.62 | 0.01 |
| 66595 | Aste1 | 2.25 | 0.00 | 1.77 | 0.02 |
| 74194 | Rnd3 | 2.25 | 0.00 | 1.35 | 0.01 |
| 70757 | Ptplb | 2.24 | 0.01 | 0.73 | 0.22 |
| 109676 | Ank2 | 2.24 | 0.05 | 2.25 | 0.04 |
| 18452 | P4ha2 | 2.24 | 0.00 | 1.00 | 1.00 |
| 72446 | 2600010E01Rik | 2.23 | 0.03 | 0.92 | 0.81 |
| 56421 | Pfkp | 2.23 | 0.01 | 2.06 | 0.01 |
| 18045 | Nfyb | 2.23 | 0.00 | 2.26 | 0.00 |
| 20530 | Slc31a2 | 2.23 | 0.01 | 1.31 | 0.09 |
| 97287 | Mtmr14 | 2.23 | 0.02 | 1.14 | 0.12 |
| 23797 | Akt3 | 2.22 | 0.00 | 1.58 | 0.00 |
| 21682 | Tec | 2.22 | 0.01 | 1.10 | 0.68 |
| 71660 | Rarres2 | 2.21 | 0.01 | 1.46 | 0.06 |
| 24059 | Slco2a1 | 2.21 | 0.02 | 0.94 | 0.82 |
| 12018 | Bak1 | 2.21 | 0.02 | 1.91 | 0.01 |
| 75788 | Smurf1 | 2.21 | 0.00 | 1.32 | 0.00 |
| 30059 | Timm10 | 2.20 | 0.00 | 1.20 | 0.08 |
| 74596 | Cds1 | 2.20 | 0.03 | 1.48 | 0.20 |
| 13709 | Elf1 | 2.19 | 0.00 | 1.74 | 0.01 |
| 17118 | Marcks | 2.19 | 0.00 | 0.88 | 0.08 |
| 72565 | Uaca | 2.19 | 0.01 | 1.32 | 0.09 |
| 12297 | Cacnb3 | 2.18 | 0.02 | 0.96 | 0.90 |
| 101985 | AA960436 | 2.17 | 0.00 | 1.30 | 0.02 |
| 14081 | Acsl1 | 2.17 | 0.00 | 1.22 | 0.11 |
| 16418 | Eif6 | 2.17 | 0.00 | 1.20 | 0.06 |
| 213753 | Zfp598 | 2.17 | 0.02 | 0.80 | 0.17 |
| 242669 | Adc | 2.16 | 0.00 | 2.38 | 0.01 |
| 19305 | Pex5 | 2.13 | 0.00 | 1.10 | 0.56 |
| 19365 | Rad52 | 2.13 | 0.00 | 1.19 | 0.11 |
| 26427 | Creb3l1 | 2.13 | 0.03 | 1.06 | 0.99 |
| 17357 | Mlp | 2.13 | 0.01 | 1.30 | 0.10 |
| 245944 | Vps54 | 2.12 | 0.00 | 1.44 | 0.06 |
| 78177 | 4930519N13Rik | 2.12 | 0.00 | 0.96 | 0.86 |
| 66388 | Cutc | 2.12 | 0.00 | 1.33 | 0.05 |
| 108954 | Ppp1r15b | 2.11 | 0.01 | 1.31 | 0.15 |
| 53857 | Tuba8 | 2.11 | 0.05 | 0.69 | 0.36 |
| 22278 | Usf1 | 2.11 | 0.02 | 1.95 | 0.01 |
| 100048649 | LOC100048649 | 2.10 | 0.04 | 1.16 | 0.53 |
| 433256 | Acsl5 | 2.09 | 0.00 | 1.62 | 0.00 |
| 68235 | 2410066E13Rik | 2.09 | 0.00 | 1.20 | 0.37 |
| 18519 | Kat2b | 2.09 | 0.03 | 2.46 | 0.00 |
| 19094 | Mapk11 | 2.09 | 0.01 | 1.01 | 0.99 |
| 70762 | Dclk2 | 2.08 | 0.02 | 1.12 | 0.67 |
| 14670 | Gnal1 | 2.08 | 0.01 | 1.51 | 0.01 |
| 106628 | Trip10 | 2.08 | 0.01 | 1.31 | 0.17 |
| 240832 | Tor1aip2 | 2.07 | 0.01 | 1.96 | 0.01 |
| 68497 | 1110018G07Rik | 2.07 | 0.00 | 1.21 | 0.11 |
| 100047762 | LOC100047762 | 2.07 | 0.00 | 1.39 | 0.04 |
| 11491 | Adam17 | 2.07 | 0.00 | 1.04 | 0.70 |
| 56738 | Mocs1 | 2.06 | 0.01 | 0.93 | 0.55 |
| 76438 | Rftn1 | 2.06 | 0.01 | 1.81 | 0.02 |
| 78830 | Slc25a12 | 2.05 | 0.01 | 1.34 | 0.14 |
| 12125 | Bcl2l11 | 2.05 | 0.00 | 0.81 | 0.07 |
| 66141 | Ifitm3 | 2.04 | 0.00 | 1.58 | 0.00 |
| 68865 | Arv1 | 2.04 | 0.00 | 1.39 | 0.00 |
| 56480 | Tbk1 | 2.04 | 0.00 | 1.27 | 0.00 |
| 268420 | Alkbh5 | 2.04 | 0.00 | 0.97 | 0.54 |
| 106672 | AI413582 | 2.04 | 0.01 | 2.33 | 0.00 |
| 17357 | Marcksl1 | 2.04 | 0.01 | 1.30 | 0.03 |
| 16195 | Il6st | 2.04 | 0.01 | 1.23 | 0.09 |
| 74442 | Sgms2 | 2.03 | 0.03 | 1.58 | 0.08 |
| 74123 | Foxp4 | 2.03 | 0.04 | 0.84 | 0.33 |
| 56722 | Litaf | 2.03 | 0.00 | 1.50 | 0.00 |
| 13852 | Stx2 | 2.03 | 0.02 | 2.03 | 0.02 |
| 72503 | 2610507B11Rik | 2.02 | 0.00 | 1.89 | 0.00 |
| 20448 | St6galnac4 | 2.02 | 0.01 | 1.60 | 0.03 |
| 217684 | 4933426M11Rik | 2.02 | 0.01 | 1.10 | 0.40 |
| 21975 | Top3a | 2.02 | 0.03 | 1.06 | 0.80 |
| 78388 | Mvp | 2.02 | 0.01 | 1.45 | 0.01 |
| 11637 | Ak2 | 2.02 | 0.00 | 1.64 | 0.00 |
| 53380 | Psmd10 | 2.01 | 0.00 | 1.00 | 0.93 |
| 21417 | Zeb1 | 2.01 | 0.00 | 1.15 | 0.13 |
| 234728 | BC025546 | 2.01 | 0.00 | 1.09 | 0.42 |
| 21353 | Tank | 2.00 | 0.00 | 1.11 | 0.22 |
| 74201 | Lrriq2 | 0.66 | 0.01 | 0.84 | 0.10 |
| 229487 | Pet112l | 0.66 | 0.01 | 0.85 | 0.07 |
| 67046 | Tbc1d7 | 0.66 | 0.00 | 0.86 | 0.11 |
| 242894 | Actr3b | 0.66 | 0.03 | 0.61 | 0.06 |
| 12070 | Ngfrap1 | 0.66 | 0.01 | 0.88 | 0.23 |
| 101543 | Wtip | 0.66 | 0.02 | 0.99 | 0.92 |
| 100047634 | LOC100047634 | 0.66 | 0.03 | 1.16 | 0.15 |
| 319625 | Galm | 0.66 | 0.00 | 0.94 | 0.41 |
| 71446 | Wrb | 0.66 | 0.00 | 0.91 | 0.17 |
| 13848 | Ephb6 | 0.66 | 0.01 | 0.77 | 0.04 |
| 66046 | Ndufb5 | 0.66 | 0.02 | 1.10 | 0.33 |
| 21833 | Thra | 0.66 | 0.02 | 0.95 | 0.71 |
| 20866 | Stim1 | 0.66 | 0.02 | 0.94 | 0.67 |
| 243362 | Stard13 | 0.66 | 0.04 | 0.93 | 0.54 |
| 66056 | Zfp524 | 0.66 | 0.01 | 0.95 | 0.68 |
| 66922 | Rras2 | 0.65 | 0.01 | 1.32 | 0.03 |
| 19655 | Rbmx | 0.65 | 0.05 | 1.00 | 0.91 |
| 68969 | Eif1b | 0.65 | 0.00 | 0.86 | 0.10 |
| 23992 | Prkra | 0.65 | 0.02 | 0.85 | 0.19 |
| 68666 | Svop | 0.65 | 0.01 | 0.80 | 0.08 |
| 56176 | Pigp | 0.65 | 0.00 | 0.86 | 0.01 |
| 238871 | Pde4d | 0.65 | 0.03 | 0.89 | 0.56 |
| 16825 | Ldb1 | 0.65 | 0.01 | 0.89 | 0.33 |
| 100045019 | LOC100045019 | 0.65 | 0.01 | 0.83 | 0.11 |
| 26940 | Ecsit | 0.65 | 0.01 | 1.02 | 0.96 |
| 235406 | Snx33 | 0.65 | 0.01 | 0.96 | 0.62 |
| 22402 | Wisp1 | 0.65 | 0.00 | 0.53 | 0.00 |
| 14874 | Gstz1 | 0.65 | 0.03 | 0.77 | 0.21 |
| 75710 | Rbm12 | 0.65 | 0.04 | 0.76 | 0.09 |
| 217716 | Mlh3 | 0.65 | 0.01 | 0.82 | 0.09 |
| 66928 | 3110001D03Rik | 0.65 | 0.00 | 0.89 | 0.08 |
| 67933 | Hcfc2 | 0.65 | 0.03 | 0.93 | 0.34 |
| 17436 | Mod1 | 0.65 | 0.05 | 1.08 | 0.62 |
| 66359 | 2310005N03Rik | 0.65 | 0.01 | 0.97 | 0.84 |
| 66049 | Rogdi | 0.65 | 0.03 | 0.83 | 0.12 |
| 68332 | 0610010E21Rik | 0.64 | 0.01 | 0.87 | 0.03 |
| 107338 | Gbf1 | 0.64 | 0.02 | 0.82 | 0.17 |
| 22697 | Zscan21 | 0.64 | 0.00 | 1.01 | 0.88 |
| 102098 | Arhgef18 | 0.64 | 0.00 | 1.03 | 0.59 |
| 27428 | Shroom3 | 0.64 | 0.02 | 1.14 | 0.29 |
| 17420 | Mnat1 | 0.64 | 0.00 | 1.00 | 0.93 |
| 59038 | Pxmp4 | 0.64 | 0.00 | 0.95 | 0.40 |
| 218232 | Ptpdc1 | 0.64 | 0.02 | 0.87 | 0.10 |
| 67392 | 4833420G17Rik | 0.64 | 0.05 | 1.16 | 0.19 |
| 68631 | Cryl1 | 0.64 | 0.05 | 0.83 | 0.32 |
| 23971 | Papss1 | 0.64 | 0.00 | 0.78 | 0.00 |
| 15184 | Hdac5 | 0.64 | 0.01 | 1.00 | 0.97 |
| 242785 | Klhl21 | 0.64 | 0.02 | 0.85 | 0.31 |
| 74270 | Usp20 | 0.64 | 0.04 | 0.93 | 0.71 |
| 192231 | Hexim1 | 0.64 | 0.01 | 0.85 | 0.09 |
| 53892 | Ppm1d | 0.64 | 0.01 | 0.81 | 0.09 |
| 56357 | Ivd | 0.64 | 0.05 | 0.82 | 0.15 |
| 99138 | Stard7 | 0.64 | 0.02 | 0.90 | 0.06 |
| 72333 | Palld | 0.64 | 0.01 | 0.83 | 0.14 |
| 12348 | Car11 | 0.64 | 0.01 | 0.76 | 0.04 |
| 231600 | Chfr | 0.64 | 0.00 | 1.01 | 0.94 |
| 59031 | Chst12 | 0.64 | 0.02 | 0.90 | 0.26 |
| 73327 | 1700040I03Rik | 0.64 | 0.01 | 0.88 | 0.10 |
| 333331 | LOC333331 | 0.64 | 0.05 | 1.06 | 0.57 |
| 216345 | Zfc3h1 | 0.64 | 0.00 | 0.82 | 0.03 |
| 100041103 | LOC100041103 | 0.64 | 0.04 | 1.28 | 0.25 |
| 104445 | Cdc42ep1 | 0.64 | 0.01 | 0.98 | 0.71 |
| 237052 | Tceal1 | 0.64 | 0.02 | 0.88 | 0.43 |
| 101113 | Snx21 | 0.64 | 0.01 | 1.06 | 0.62 |
| 70178 | 2210412D01Rik | 0.63 | 0.04 | 1.60 | 0.04 |
| 211914 | Ddef2 | 0.63 | 0.00 | 0.69 | 0.07 |
| 13360 | Dhcr7 | 0.63 | 0.01 | 0.83 | 0.11 |
| 64436 | Inpp5e | 0.63 | 0.00 | 0.84 | 0.00 |
| 69928 | Apitd1 | 0.63 | 0.01 | 0.70 | 0.00 |
| 67306 | Fam164a | 0.63 | 0.00 | 1.00 | 0.95 |
| 11732 | Ank | 0.63 | 0.00 | 0.73 | 0.00 |
| 58996 | 4933428G20Rik | 0.63 | 0.00 | 0.97 | 0.56 |
| 105278 | Ccrk | 0.63 | 0.00 | 0.84 | 0.06 |
| 67689 | Aldh3b1 | 0.63 | 0.05 | 0.90 | 0.58 |
| 74772 | Atp13a2 | 0.63 | 0.03 | 0.74 | 0.04 |
| 209039 | Tenc1 | 0.63 | 0.02 | 0.98 | 0.93 |
| 208647 | Creb3l2 | 0.63 | 0.00 | 0.70 | 0.01 |
| 231801 | Agfg2 | 0.63 | 0.01 | 0.92 | 0.34 |
| 57874 | Ptplad1 | 0.63 | 0.00 | 0.69 | 0.01 |
| 19090 | Prkdc | 0.63 | 0.01 | 0.72 | 0.06 |
| 269639 | Zfp512 | 0.63 | 0.01 | 0.86 | 0.22 |
| 68385 | Tlcd1 | 0.63 | 0.04 | 0.75 | 0.14 |
| 21987 | Tpd52l1 | 0.63 | 0.00 | 0.72 | 0.00 |
| 67306 | 3110050N22Rik | 0.63 | 0.02 | 1.26 | 0.19 |
| 68659 | 1110032E23Rik | 0.63 | 0.03 | 0.92 | 0.57 |
| 16661 | Krt10 | 0.63 | 0.00 | 0.84 | 0.02 |
| 56737 | Alg2 | 0.62 | 0.01 | 0.85 | 0.03 |
| 73284 | Ddit4l | 0.62 | 0.04 | 0.94 | 0.55 |
| 69253 | Hspb2 | 0.62 | 0.01 | 0.93 | 0.59 |
| 26401 | Map3k1 | 0.62 | 0.03 | 0.95 | 0.57 |
| 226252 | AI450540 | 0.62 | 0.00 | 0.82 | 0.00 |
| 93840 | Vangl2 | 0.62 | 0.04 | 0.69 | 0.12 |
| 66129 | 1110018J18Rik | 0.62 | 0.04 | 0.79 | 0.19 |
| 66905 | M6prbp1 | 0.62 | 0.02 | 0.95 | 0.63 |
| 59056 | Evc | 0.62 | 0.00 | 0.81 | 0.02 |
| 19727 | Rfxank | 0.62 | 0.03 | 0.79 | 0.34 |
| 230101 | Gba2 | 0.62 | 0.01 | 0.78 | 0.04 |
| 214901 | Chtf18 | 0.62 | 0.05 | 0.75 | 0.26 |
| 107995 | Cdc20 | 0.62 | 0.03 | 0.91 | 0.50 |
| 11841 | Arf2 | 0.62 | 0.02 | 0.83 | 0.14 |
| 72139 | 2610044O15Rik | 0.62 | 0.03 | 0.85 | 0.28 |
| 319757 | Smo | 0.62 | 0.03 | 0.87 | 0.41 |
| 66836 | 0610006I08Rik | 0.62 | 0.00 | 0.81 | 0.06 |
| 66090 | Ypel3 | 0.62 | 0.04 | 0.85 | 0.29 |
| 228410 | Cstf3 | 0.62 | 0.01 | 0.78 | 0.02 |
| 66522 | Pgpep1 | 0.62 | 0.01 | 1.17 | 0.25 |
| 56017 | Slc2a8 | 0.61 | 0.01 | 0.73 | 0.01 |
| 18483 | Palm | 0.61 | 0.02 | 1.08 | 0.50 |
| 66273 | 1810020D17Rik | 0.61 | 0.00 | 0.86 | 0.12 |
| 20598 | Smpd2 | 0.61 | 0.01 | 0.77 | 0.10 |
| 68977 | Haghl | 0.61 | 0.00 | 0.80 | 0.02 |
| 74246 | Gale | 0.61 | 0.05 | 0.83 | 0.30 |
| 73451 | 1700065O13Rik | 0.61 | 0.01 | 0.96 | 0.76 |
| 231912 | Katnal1 | 0.61 | 0.01 | 0.82 | 0.11 |
| 328801 | Zfp414 | 0.61 | 0.00 | 0.76 | 0.01 |
| 66704 | Rbm4b | 0.60 | 0.05 | 0.82 | 0.12 |
| 107568 | Wwp1 | 0.60 | 0.04 | 0.84 | 0.34 |
| 110078 | Pygb | 0.60 | 0.01 | 0.91 | 0.37 |
| 67774 | Loh12cr1 | 0.60 | 0.01 | 0.80 | 0.08 |
| 21679 | Tead4 | 0.60 | 0.04 | 0.89 | 0.29 |
| 223593 | E430025E21Rik | 0.60 | 0.02 | 1.04 | 0.76 |
| 103677 | Smg6 | 0.60 | 0.01 | 0.75 | 0.09 |
| 12159 | Bmp4 | 0.60 | 0.01 | 1.19 | 0.09 |
| 17826 | Mtvr2 | 0.60 | 0.00 | 0.91 | 0.27 |
| 70747 | Tspan2 | 0.60 | 0.03 | 0.68 | 0.05 |
| 544922 | OTTMUSG00000000421 | 0.60 | 0.02 | 0.80 | 0.28 |
| 319480 | Itga11 | 0.60 | 0.01 | 0.72 | 0.07 |
| 26404 | Map3k12 | 0.60 | 0.02 | 0.81 | 0.14 |
| 18709 | Pik3r2 | 0.60 | 0.01 | 0.84 | 0.03 |
| 76478 | 2410004L22Rik | 0.60 | 0.01 | 0.75 | 0.06 |
| 74729 | Setmar | 0.60 | 0.01 | 0.70 | 0.04 |
| 23821 | Bace1 | 0.60 | 0.01 | 0.93 | 0.66 |
| 260409 | Cdc42ep3 | 0.60 | 0.01 | 0.93 | 0.53 |
| 22718 | Zfp60 | 0.60 | 0.00 | 0.75 | 0.02 |
| 71670 | Acy3 | 0.60 | 0.00 | 0.72 | 0.04 |
| 235674 | Acaa1b | 0.60 | 0.01 | 0.72 | 0.09 |
| 352968 | D830050J10Rik | 0.60 | 0.03 | 0.83 | 0.23 |
| 231670 | Fbxo21 | 0.59 | 0.01 | 0.83 | 0.04 |
| 76108 | Rap2a | 0.59 | 0.01 | 1.19 | 0.13 |
| 11481 | Acvr2b | 0.59 | 0.01 | 0.91 | 0.50 |
| 66648 | 5730494M16Rik | 0.59 | 0.00 | 0.78 | 0.02 |
| 68968 | Cdan1 | 0.59 | 0.00 | 0.80 | 0.01 |
| 97031 | C430004E15Rik | 0.59 | 0.01 | 0.81 | 0.04 |
| 330695 | Ctxn1 | 0.59 | 0.03 | 0.76 | 0.12 |
| 104732 | 4930427A07Rik | 0.59 | 0.04 | 0.71 | 0.17 |
| 319876 | Cobll1 | 0.59 | 0.04 | 0.98 | 0.85 |
| 69534 | Avpi1 | 0.59 | 0.01 | 0.89 | 0.17 |
| 171580 | Mical1 | 0.59 | 0.01 | 0.91 | 0.21 |
| 233103 | 4931406P16Rik | 0.59 | 0.01 | 0.80 | 0.15 |
| 226791 | Lyplal1 | 0.59 | 0.05 | 0.74 | 0.13 |
| 234686 | Fhod1 | 0.59 | 0.00 | 0.86 | 0.24 |
| 12036 | Bcat2 | 0.59 | 0.01 | 0.92 | 0.44 |
| 211586 | Tfdp2 | 0.58 | 0.01 | 0.96 | 0.71 |
| 18612 | Etv4 | 0.58 | 0.02 | 0.61 | 0.01 |
| 217140 | Scrn2 | 0.58 | 0.03 | 0.77 | 0.12 |
| 68283 | 9530077C05Rik | 0.58 | 0.02 | 1.26 | 0.23 |
| 26901 | Deb1 | 0.58 | 0.00 | 0.73 | 0.01 |
| 213027 | Evi5l | 0.58 | 0.00 | 0.77 | 0.02 |
| 68636 | Fahd1 | 0.58 | 0.04 | 0.77 | 0.11 |
| 108097 | Prkab2 | 0.58 | 0.03 | 0.61 | 0.00 |
| 67993 | Nudt12 | 0.58 | 0.00 | 0.78 | 0.05 |
| 13019 | Ctf1 | 0.58 | 0.01 | 0.74 | 0.02 |
| 100044566 | LOC100044566 | 0.58 | 0.02 | 1.00 | 0.95 |
| 353502 | Hcfc1r1 | 0.58 | 0.03 | 0.81 | 0.22 |
| 223864 | Rapgef3 | 0.57 | 0.04 | 0.71 | 0.17 |
| 107351 | Kank1 | 0.57 | 0.00 | 0.84 | 0.05 |
| 19301 | Pxmp2 | 0.57 | 0.05 | 0.58 | 0.08 |
| 68510 | Ints1 | 0.57 | 0.00 | 0.87 | 0.19 |
| 18604 | Pdk2 | 0.57 | 0.03 | 0.75 | 0.05 |
| 67298 | Gprasp1 | 0.57 | 0.03 | 0.76 | 0.22 |
| 101772 | Ano1 | 0.57 | 0.00 | 0.66 | 0.02 |
| 121022 | Mrps6 | 0.57 | 0.00 | 0.86 | 0.13 |
| 11364 | Acadm | 0.57 | 0.00 | 0.92 | 0.38 |
| 209773 | Dennd2a | 0.57 | 0.01 | 0.76 | 0.07 |
| 21886 | Tle2 | 0.57 | 0.04 | 0.67 | 0.15 |
| 232337 | Zfp637 | 0.57 | 0.00 | 0.85 | 0.02 |
| 207278 | Fchsd2 | 0.57 | 0.01 | 0.90 | 0.29 |
| 217351 | Tnrc6c | 0.57 | 0.01 | 0.85 | 0.19 |
| 17347 | Mknk2 | 0.57 | 0.04 | 0.77 | 0.08 |
| 217166 | Nr1d1 | 0.57 | 0.02 | 0.78 | 0.16 |
| 319604 | Fam168a | 0.56 | 0.01 | 0.84 | 0.23 |
| 18162 | Npr3 | 0.56 | 0.01 | 1.05 | 0.81 |
| 106795 | Tcf19 | 0.56 | 0.04 | 0.92 | 0.52 |
| 217310 | C630004H02Rik | 0.56 | 0.02 | 0.74 | 0.15 |
| 50708 | Hist1h1c | 0.56 | 0.04 | 0.78 | 0.17 |
| 14461 | Gata2 | 0.56 | 0.00 | 0.92 | 0.11 |
| 210719 | Mkx | 0.56 | 0.00 | 0.70 | 0.12 |
| 320234 | Ccdc66 | 0.56 | 0.01 | 0.77 | 0.06 |
| 14794 | Spsb2 | 0.56 | 0.00 | 1.15 | 0.14 |
| 79059 | Nme3 | 0.56 | 0.03 | 0.70 | 0.09 |
| 66532 | 2210417D09Rik | 0.55 | 0.03 | 0.76 | 0.13 |
| 227094 | 5330401P04Rik | 0.55 | 0.04 | 0.74 | 0.24 |
| 59095 | Fxyd6 | 0.55 | 0.02 | 0.67 | 0.09 |
| 216190 | Appl2 | 0.55 | 0.01 | 0.68 | 0.08 |
| 211586 | A330080J22Rik | 0.55 | 0.02 | 0.93 | 0.56 |
| 214424 | Parp16 | 0.55 | 0.01 | 0.73 | 0.17 |
| 208967 | Thnsl1 | 0.55 | 0.00 | 0.76 | 0.07 |
| 74764 | Klc4 | 0.55 | 0.00 | 0.86 | 0.20 |
| 73296 | Rhobtb3 | 0.55 | 0.00 | 0.81 | 0.02 |
| 17540 | Mrvi1 | 0.55 | 0.03 | 0.87 | 0.46 |
| 107328 | Trpt1 | 0.55 | 0.00 | 0.78 | 0.08 |
| 215474 | Sec22c | 0.55 | 0.00 | 0.90 | 0.45 |
| 50496 | E2f6 | 0.55 | 0.00 | 0.92 | 0.37 |
| 18858 | Pmp22 | 0.55 | 0.00 | 1.13 | 0.26 |
| 73095 | Slc25a42 | 0.55 | 0.03 | 0.72 | 0.18 |
| 14924 | Magi1 | 0.55 | 0.04 | 0.74 | 0.13 |
| 56520 | Nme4 | 0.55 | 0.03 | 0.75 | 0.20 |
| 73680 | 2410081M15Rik | 0.55 | 0.02 | 1.01 | 0.98 |
| 14026 | Evl | 0.55 | 0.00 | 0.93 | 0.60 |
| 19725 | Rfx2 | 0.55 | 0.03 | 1.09 | 0.80 |
| 12580 | Cdkn2c | 0.55 | 0.02 | 0.89 | 0.57 |
| 74202 | Fblim1 | 0.55 | 0.02 | 0.75 | 0.21 |
| 66355 | Gmpr | 0.54 | 0.01 | 0.84 | 0.27 |
| 16886 | Limk2 | 0.54 | 0.00 | 0.83 | 0.04 |
| 83429 | Ctns | 0.54 | 0.00 | 0.87 | 0.06 |
| 232811 | Suv420h2 | 0.54 | 0.02 | 0.97 | 0.83 |
| 242705 | E2f2 | 0.54 | 0.04 | 0.65 | 0.05 |
| 83669 | Wdr6 | 0.54 | 0.04 | 0.85 | 0.49 |
| 70784 | Rasl12 | 0.54 | 0.00 | 0.96 | 0.60 |
| 12009 | Azi1 | 0.54 | 0.00 | 0.73 | 0.01 |
| 15404 | Hoxa7 | 0.54 | 0.01 | 1.01 | 0.95 |
| 15586 | Hyal1 | 0.54 | 0.04 | 0.85 | 0.20 |
| 104175 | Sbk | 0.54 | 0.00 | 0.77 | 0.01 |
| 57170 | Dolpp1 | 0.54 | 0.00 | 0.89 | 0.26 |
| 245847 | Amdhd2 | 0.54 | 0.00 | 0.88 | 0.36 |
| 14173 | Fgf2 | 0.54 | 0.05 | 0.76 | 0.37 |
| 100201 | 9630015D15Rik | 0.54 | 0.00 | 0.73 | 0.01 |
| 23882 | Gadd45g | 0.54 | 0.02 | 1.35 | 0.09 |
| 54722 | Dfna5h | 0.54 | 0.00 | 0.87 | 0.16 |
| 108099 | Prkag2 | 0.54 | 0.05 | 0.92 | 0.85 |
| 70266 | Ccbl1 | 0.54 | 0.02 | 0.62 | 0.02 |
| 20744 | Strbp | 0.54 | 0.00 | 0.79 | 0.13 |
| 269831 | Tspan12 | 0.53 | 0.00 | 0.71 | 0.03 |
| 72033 | Tsc22d2 | 0.53 | 0.00 | 0.92 | 0.44 |
| 229285 | Spg20 | 0.53 | 0.00 | 0.74 | 0.01 |
| 85308 | Fam158a | 0.53 | 0.00 | 0.91 | 0.51 |
| 21808 | Tgfb2 | 0.53 | 0.01 | 0.97 | 0.68 |
| 72154 | 2610020C11Rik | 0.53 | 0.02 | 0.65 | 0.07 |
| 69551 | 2310022B05Rik | 0.53 | 0.00 | 1.09 | 0.36 |
| 329470 | Accs | 0.53 | 0.00 | 0.76 | 0.06 |
| 100213 | Rusc2 | 0.53 | 0.00 | 0.76 | 0.05 |
| 21809 | Tgfb3 | 0.53 | 0.00 | 0.76 | 0.05 |
| 80288 | Bcl9l | 0.53 | 0.00 | 0.80 | 0.01 |
| 106565 | Dlk2 | 0.53 | 0.02 | 1.08 | 0.63 |
| 75735 | Pank1 | 0.53 | 0.02 | 0.87 | 0.56 |
| 208659 | Fam20a | 0.53 | 0.00 | 0.81 | 0.08 |
| 56016 | Hebp2 | 0.53 | 0.01 | 0.72 | 0.08 |
| 14367 | Fzd5 | 0.53 | 0.00 | 0.85 | 0.37 |
| 54201 | Zfp316 | 0.53 | 0.00 | 0.81 | 0.15 |
| 67017 | 2010011I20Rik | 0.53 | 0.00 | 0.68 | 0.01 |
| 12325 | Camk2g | 0.52 | 0.01 | 0.92 | 0.60 |
| 71904 | Paqr7 | 0.52 | 0.00 | 0.82 | 0.06 |
| 338350 | 9330129D05Rik | 0.52 | 0.04 | 0.83 | 0.46 |
| 69215 | Sat2 | 0.52 | 0.02 | 0.74 | 0.11 |
| 217219 | Fam171a2 | 0.52 | 0.00 | 0.85 | 0.08 |
| 83815 | Cenpq | 0.52 | 0.01 | 1.19 | 0.26 |
| 74769 | Pik3cb | 0.52 | 0.01 | 1.04 | 0.81 |
| 20592 | Jarid1d | 0.52 | 0.00 | 0.85 | 0.28 |
| 212647 | Aldh4a1 | 0.52 | 0.00 | 0.82 | 0.12 |
| 332397 | Nanos1 | 0.52 | 0.02 | 0.90 | 0.28 |
| 211401 | Mtss1 | 0.52 | 0.02 | 1.07 | 0.81 |
| 14368 | Fzd6 | 0.52 | 0.01 | 0.71 | 0.14 |
| 15925 | Ide | 0.51 | 0.01 | 0.56 | 0.01 |
| 54169 | Myst4 | 0.51 | 0.02 | 0.86 | 0.46 |
| 12064 | Bdnf | 0.51 | 0.00 | 1.03 | 0.83 |
| 100040799 | LOC100040799 | 0.51 | 0.02 | 0.78 | 0.30 |
| 71998 | Slc25a35 | 0.51 | 0.00 | 0.76 | 0.10 |
| 21422 | Tcfcp2 | 0.51 | 0.00 | 0.64 | 0.00 |
| 245522 | Zc4h2 | 0.51 | 0.00 | 0.83 | 0.26 |
| 56018 | Stard10 | 0.51 | 0.01 | 0.90 | 0.42 |
| 269424 | Phf17 | 0.51 | 0.02 | 0.94 | 0.71 |
| 14388 | Gab1 | 0.51 | 0.01 | 1.01 | 0.84 |
| 269608 | Plekhg5 | 0.50 | 0.03 | 0.74 | 0.22 |
| 13590 | Lefty1 | 0.50 | 0.00 | 0.71 | 0.00 |
| 269152 | Kif26b | 0.50 | 0.03 | 0.65 | 0.11 |
| 70717 | 6330406I15Rik | 0.50 | 0.04 | 0.67 | 0.06 |
| 108686 | Ccdc88a | 0.50 | 0.00 | 0.73 | 0.01 |
| 72630 | Hspa12b | 0.50 | 0.04 | 0.62 | 0.16 |
| 22701 | Zfp41 | 0.50 | 0.02 | 0.77 | 0.24 |
| 230793 | Ahdc1 | 0.50 | 0.03 | 0.84 | 0.53 |
| 67862 | 2310033P09Rik | 0.50 | 0.00 | 0.78 | 0.01 |
| 12323 | Camk2b | 0.49 | 0.02 | 0.70 | 0.10 |
| 14709 | Gng8 | 0.49 | 0.00 | 0.74 | 0.10 |
| 22693 | Zfp30 | 0.49 | 0.00 | 0.88 | 0.04 |
| 230752 | 2610027C15Rik | 0.49 | 0.03 | 0.74 | 0.13 |
| 16007 | Cyr61 | 0.49 | 0.01 | 1.05 | 0.69 |
| 107986 | Ddb2 | 0.49 | 0.00 | 0.64 | 0.02 |
| 56279 | B230317C12Rik | 0.49 | 0.01 | 0.75 | 0.10 |
| 233315 | BB128963 | 0.49 | 0.01 | 0.94 | 0.74 |
| 72749 | Nfkbil2 | 0.49 | 0.04 | 0.66 | 0.18 |
| 399566 | Btbd6 | 0.49 | 0.00 | 0.98 | 0.61 |
| 94281 | Sfxn4 | 0.49 | 0.00 | 0.74 | 0.07 |
| 22379 | Fmnl3 | 0.49 | 0.00 | 0.69 | 0.04 |
| 71774 | 1300007L22Rik | 0.49 | 0.02 | 0.82 | 0.40 |
| 72748 | Hdhd3 | 0.49 | 0.03 | 0.69 | 0.02 |
| 13614 | Edn1 | 0.48 | 0.00 | 0.75 | 0.05 |
| 245670 | Rragb | 0.48 | 0.05 | 0.69 | 0.25 |
| 67304 | 3110070M22Rik | 0.48 | 0.00 | 0.68 | 0.00 |
| 66431 | 1810049H13Rik | 0.48 | 0.03 | 0.74 | 0.11 |
| 13549 | Dyrk1b | 0.48 | 0.01 | 1.02 | 0.91 |
| 66985 | Rassf7 | 0.48 | 0.01 | 1.01 | 0.91 |
| 211652 | Wwc1 | 0.48 | 0.00 | 0.84 | 0.10 |
| 114663 | Impa2 | 0.48 | 0.01 | 0.85 | 0.40 |
| 231532 | Arhgap24 | 0.48 | 0.00 | 0.79 | 0.04 |
| 20324 | Sdpr | 0.48 | 0.00 | 0.93 | 0.51 |
| 71279 | Slc29a3 | 0.47 | 0.00 | 0.73 | 0.01 |
| 72507 | 2610524A10Rik | 0.47 | 0.01 | 0.78 | 0.10 |
| 208518 | Cep78 | 0.47 | 0.00 | 0.74 | 0.01 |
| 105988 | Espl1 | 0.47 | 0.02 | 0.74 | 0.34 |
| 67379 | Dedd2 | 0.47 | 0.00 | 0.96 | 0.77 |
| 76467 | Msrb2 | 0.47 | 0.00 | 0.63 | 0.00 |
| 235431 | Coro2b | 0.47 | 0.04 | 0.50 | 0.01 |
| 278240 | Spin2 | 0.46 | 0.03 | 0.54 | 0.01 |
| 213056 | BC049806 | 0.46 | 0.00 | 0.60 | 0.00 |
| 57434 | Xrcc2 | 0.46 | 0.04 | 0.63 | 0.12 |
| 17921 | Myo7a | 0.46 | 0.00 | 0.61 | 0.00 |
| 269951 | Idh2 | 0.46 | 0.02 | 1.02 | 0.82 |
| 21917 | Tmpo | 0.46 | 0.02 | 0.95 | 0.84 |
| 20346 | Sema3a | 0.46 | 0.01 | 0.68 | 0.09 |
| 213573 | Efcab4a | 0.46 | 0.00 | 0.73 | 0.02 |
| 21899 | Tlr6 | 0.45 | 0.02 | 0.87 | 0.55 |
| 236899 | Pcyt1b | 0.45 | 0.01 | 0.86 | 0.53 |
| 12416 | Cbx2 | 0.45 | 0.00 | 0.71 | 0.18 |
| 17268 | Meis1 | 0.45 | 0.00 | 0.85 | 0.18 |
| 50524 | Sall2 | 0.45 | 0.00 | 0.68 | 0.02 |
| 226744 | 9630058J23Rik | 0.45 | 0.00 | 0.85 | 0.32 |
| 21415 | Tcf3 | 0.45 | 0.00 | 0.78 | 0.04 |
| 71436 | Flrt3 | 0.45 | 0.01 | 0.74 | 0.10 |
| 30951 | Cbx8 | 0.45 | 0.00 | 0.83 | 0.10 |
| 76976 | 2900062L11Rik | 0.45 | 0.01 | 0.81 | 0.28 |
| 26568 | Slc27a3 | 0.44 | 0.02 | 0.76 | 0.28 |
| 13631 | Eef2k | 0.44 | 0.01 | 0.85 | 0.32 |
| 14070 | F8a | 0.44 | 0.00 | 0.84 | 0.12 |
| 213019 | Pdlim2 | 0.44 | 0.01 | 0.80 | 0.07 |
| 53310 | Dlg3 | 0.44 | 0.01 | 0.80 | 0.15 |
| 107250 | Kazald1 | 0.44 | 0.01 | 0.95 | 0.90 |
| 215114 | Hip1 | 0.44 | 0.00 | 0.91 | 0.45 |
| 213649 | Arhgef19 | 0.44 | 0.04 | 0.76 | 0.42 |
| 78779 | Spata2L | 0.43 | 0.00 | 0.80 | 0.05 |
| 29856 | Smtn | 0.43 | 0.00 | 0.60 | 0.00 |
| 56405 | Dusp14 | 0.43 | 0.00 | 0.74 | 0.01 |
| 66949 | Trim59 | 0.43 | 0.01 | 0.97 | 0.55 |
| 54635 | Pdgfc | 0.42 | 0.02 | 0.75 | 0.25 |
| 15402 | Hoxa5 | 0.42 | 0.02 | 0.89 | 0.64 |
| 229949 | Ak5 | 0.42 | 0.03 | 0.62 | 0.15 |
| 546161 | C85627 | 0.42 | 0.00 | 0.69 | 0.09 |
| 272396 | Tarsl2 | 0.41 | 0.00 | 0.63 | 0.01 |
| 71706 | Slc46a3 | 0.41 | 0.00 | 0.84 | 0.30 |
| 76454 | Fbxo31 | 0.41 | 0.00 | 0.81 | 0.07 |
| 20887 | Sult1a1 | 0.41 | 0.01 | 0.58 | 0.03 |
| 20440 | St6gal1 | 0.41 | 0.03 | 0.94 | 0.80 |
| 100048332 | LOC100048332 | 0.40 | 0.00 | 0.56 | 0.04 |
| 233335 | Synm | 0.40 | 0.02 | 0.66 | 0.19 |
| 98496 | 5033414K04Rik | 0.40 | 0.00 | 0.75 | 0.01 |
| 93691 | Klf7 | 0.40 | 0.00 | 0.93 | 0.62 |
| 106369 | Ypel1 | 0.40 | 0.01 | 0.71 | 0.20 |
| 18710 | Pik3r3 | 0.40 | 0.01 | 0.62 | 0.07 |
| 15401 | Hoxa4 | 0.40 | 0.01 | 1.13 | 0.72 |
| 13860 | Eps8 | 0.39 | 0.02 | 0.88 | 0.71 |
| 26358 | Aldh1a7 | 0.39 | 0.00 | 0.67 | 0.02 |
| 20289 | Scx | 0.39 | 0.01 | 0.80 | 0.32 |
| 21401 | Tcea3 | 0.39 | 0.01 | 0.82 | 0.40 |
| 77976 | Nuak1 | 0.39 | 0.00 | 0.75 | 0.08 |
| 11891 | Rab27a | 0.39 | 0.04 | 0.60 | 0.17 |
| 11522 | Adh1 | 0.38 | 0.01 | 0.54 | 0.02 |
| 242297 | Fam110b | 0.38 | 0.00 | 0.77 | 0.03 |
| 102371 | AA407270 | 0.38 | 0.01 | 0.81 | 0.42 |
| 240168 | Rasgrp3 | 0.38 | 0.01 | 0.77 | 0.00 |
| 27355 | X99384 | 0.38 | 0.00 | 0.63 | 0.02 |
| 68067 | 3010026O09Rik | 0.38 | 0.03 | 0.71 | 0.18 |
| 225341 | Lims2 | 0.38 | 0.00 | 0.99 | 0.94 |
| 106639 | AI662250 | 0.38 | 0.00 | 0.71 | 0.01 |
| 230761 | BC039093 | 0.38 | 0.00 | 0.73 | 0.07 |
| 381175 | Ccdc68 | 0.37 | 0.05 | 1.00 | 0.92 |
| 13639 | Efna4 | 0.37 | 0.00 | 0.91 | 0.42 |
| 26930 | Ppnr | 0.37 | 0.03 | 0.81 | 0.63 |
| 93689 | Lmod1 | 0.37 | 0.00 | 0.77 | 0.18 |
| 192786 | Rapgef6 | 0.37 | 0.00 | 0.94 | 0.31 |
| 16170 | Il16 | 0.37 | 0.02 | 1.06 | 0.80 |
| 72852 | 2900024O10Rik | 0.37 | 0.00 | 0.76 | 0.05 |
| 17385 | Mmp11 | 0.37 | 0.01 | 0.75 | 0.23 |
| 329910 | Acot11 | 0.36 | 0.00 | 0.66 | 0.03 |
| 232174 | Cyp26b1 | 0.36 | 0.01 | 1.00 | 0.97 |
| 21419 | Tcfap2b | 0.36 | 0.02 | 0.85 | 0.63 |
| 77045 | Bcl7a | 0.36 | 0.01 | 0.64 | 0.05 |
| 14183 | Fgfr2 | 0.36 | 0.02 | 0.78 | 0.36 |
| 15478 | Hs3st3a1 | 0.36 | 0.01 | 0.52 | 0.04 |
| 381409 | Cdh26 | 0.36 | 0.02 | 0.58 | 0.19 |
| 68490 | Zfp579 | 0.35 | 0.01 | 0.99 | 0.99 |
| 14302 | Frk | 0.35 | 0.01 | 0.74 | 0.01 |
| 14200 | Fhl2 | 0.35 | 0.00 | 0.91 | 0.36 |
| 69274 | Ctdspl | 0.35 | 0.00 | 0.71 | 0.07 |
| 58226 | Cacna1h | 0.35 | 0.01 | 0.61 | 0.02 |
| 208117 | Aph1b | 0.34 | 0.00 | 0.71 | 0.00 |
| 15213 | Hey1 | 0.34 | 0.01 | 0.57 | 0.06 |
| 20947 | Swap70 | 0.34 | 0.00 | 1.06 | 0.74 |
| 15460 | Hr | 0.34 | 0.00 | 0.75 | 0.04 |
| 230991 | B930041F14Rik | 0.33 | 0.01 | 0.61 | 0.10 |
| 23937 | Mab21l2 | 0.33 | 0.00 | 0.75 | 0.07 |
| 18003 | Nedd9 | 0.32 | 0.00 | 0.69 | 0.07 |
| 226352 | Epb4.1l5 | 0.32 | 0.00 | 0.69 | 0.00 |
| 83767 | Wasf1 | 0.32 | 0.00 | 0.81 | 0.18 |
| 15399 | Hoxa2 | 0.32 | 0.03 | 0.80 | 0.60 |
| 11555 | Adrb2 | 0.31 | 0.01 | 0.85 | 0.39 |
| 231633 | Tmem119 | 0.31 | 0.04 | 0.62 | 0.31 |
| 64929 | Scel | 0.31 | 0.00 | 0.98 | 0.80 |
| 211577 | Mrgprf | 0.31 | 0.01 | 0.88 | 0.59 |
| 54366 | Ctnnal1 | 0.30 | 0.00 | 0.60 | 0.00 |
| 215748 | Cnksr3 | 0.30 | 0.00 | 0.94 | 0.36 |
| 70788 | Klhl30 | 0.30 | 0.00 | 0.70 | 0.02 |
| 57265 | Fzd2 | 0.29 | 0.01 | 0.77 | 0.41 |
| 14281 | Fos | 0.29 | 0.04 | 1.06 | 0.99 |
| 30937 | Lmcd1 | 0.29 | 0.00 | 1.32 | 0.03 |
| 100342 | 4732473B16Rik | 0.29 | 0.00 | 0.93 | 0.73 |
| 192734 | AI646023 | 0.28 | 0.00 | 0.63 | 0.12 |
| 21390 | Tbxa2r | 0.28 | 0.03 | 0.68 | 0.39 |
| 272636 | D9Ertd280e | 0.27 | 0.02 | 0.54 | 0.10 |
| 19339 | Rab3a | 0.26 | 0.00 | 0.61 | 0.00 |
| 269023 | Zfp608 | 0.26 | 0.00 | 0.75 | 0.23 |
| 27528 | D0H4S114 | 0.26 | 0.00 | 0.30 | 0.01 |
| 66895 | 1300014I06Rik | 0.25 | 0.00 | 0.73 | 0.01 |
| 13170 | Dbp | 0.25 | 0.00 | 0.83 | 0.34 |
| 77889 | Lbh | 0.24 | 0.02 | 0.83 | 0.65 |
| 215690 | Nav1 | 0.24 | 0.00 | 0.78 | 0.40 |
| 244867 | Arhgap20 | 0.23 | 0.01 | 0.80 | 0.56 |
| 20682 | Sox9 | 0.23 | 0.00 | 1.02 | 0.97 |
| 72293 | Nkd2 | 0.22 | 0.00 | 0.78 | 0.38 |
| 68918 | 1190005I06Rik | 0.22 | 0.01 | 0.64 | 0.14 |
| 494504 | Apcdd1 | 0.22 | 0.04 | 0.56 | 0.35 |
| 14370 | Fzd8 | 0.22 | 0.02 | 0.82 | 0.56 |
| 71721 | Fam13c | 0.21 | 0.00 | 0.85 | 0.07 |
| 217410 | Trib2 | 0.21 | 0.00 | 0.76 | 0.26 |
| 15214 | Hey2 | 0.20 | 0.01 | 0.75 | 0.40 |
| 19736 | Rgs4 | 0.19 | 0.02 | 0.77 | 0.48 |
| 114301 | Palmd | 0.19 | 0.00 | 0.60 | 0.13 |
| 67839 | Gpsm1 | 0.15 | 0.00 | 0.63 | 0.03 |
| 53412 | Ppp1r3c | 0.15 | 0.00 | 0.49 | 0.00 |
| 320365 | 9330186A19Rik | 0.13 | 0.00 | 0.56 | 0.16 |
| 14373 | G0s2 | 0.12 | 0.01 | 0.55 | 0.29 |
