# Supplementary material for: STAT1-Dependent Signal Integration between IFNγ and TLR4 in Vascular Cells Reflect Pro-Atherogenic Responses in Human Atherosclerosis
Source: PLoS One. 2014 Dec 5;9(12):e113318. doi: 10.1371/journal.pone.0113318 (PMC4257532; doi:10.1371/journal.pone.0113318)
Supplement: Table S4 — Primer sequences used in experimental procedures. (DOCX) [file pone.0113318.s004.docx]

### Table S4. Primer sequences used in experimental procedures.

| **Gene Name** | **Forward** | **Reverse** |
| --- | --- | --- |
| Gapdh | TCGGTGTGAACGGATTTGGC | TTTGGCTCCACCCTTCAAGTG |
| Irf8 | GCAGGATGTGTGACCGGAAC | CCACCTCCTGATTGTAATCCT |
| Ccl5 (Rantes) | CGCACCTGCCTCACCATAT | CACTTCTTCTCTGGGTTGGC |
| Cxcl10 | TCATCCCTGCGAGCCTATCC | GGAGCCCTTTTAGACCTTTTT |
| Cxcl9 | CTGCCATGAAGTCCGCTGTTCT | TCCCCCTCTTTTGCTTTTTCTT |
| Ccl12 | AGCTACCACCATCAGTCCTCA | CAAGGATGAAGGTTTGAGACG |
| Ccrl2 | ACAGTACGACCTCCACAAGC | GGAACAGGCTGCGAAGGTAT |
| GAPDH _human | CAACTGCTTAGCACCCCTGG | CAGGTCAGGTCCACCACTGA |
| IRF8_human | GGGAGAATGAGGAGAAGAGCA | CCGCACTCCATCTCTGTAACT |
